# A new alvarezsaurid dinosaur (Theropoda, Alvarezsauria) from the Upper Cretaceous Baruungoyot Formation of Mongolia provides insights for bird-like sleeping behavior in non-avian dinosaurs

**DOI:** 10.1371/journal.pone.0293801

**Published:** 2023-11-15

**Authors:** Kohta Kubo, Yoshitsugu Kobayashi, Tsogtbaatar Chinzorig, Khishigjav Tsogtbaatar

**Affiliations:** 1 Department of Natural History and Planetary Sciences, Hokkaido University, Kita-ku, Sapporo, Hokkaido, Japan; 2 Hokkaido University Museum, Kita-ku, Sapporo, Hokkaido, Japan; 3 Department of Biological Sciences, North Carolina State University, Raleigh, NC, United States of America; 4 Institute of Paleontology, Mongolian Academy of Sciences, Ulaanbaatar, Mongolia; Universiteit Maastricht, NETHERLANDS

## Abstract

Alvarezsauria is a group of early-branching maniraptoran theropods that are distributed globally from the Late Jurassic to the latest Cretaceous. Despite recent increases in the fossil record of this group, the scarcity of complete specimens still restricts interpreting their detailed anatomy, ecology, and evolution. Here, we report a new taxon of derived alvarezsaur, *Jaculinykus yaruui* gen. et sp. nov., from the Late Cretaceous of Mongolia, which represents a nearly complete and articulated skeleton. Our phylogenetic analysis reveals that *Jaculinykus* belongs to the sub-clade of Alvarezsauridae, Parvicursorinae, and forms a mononphyletic group with *Mononykus* and *Shuvuuia*. Its well-preserved manus has only two fingers, composed of a hypertrophied digit I and greatly reduced digit II, which implies an intermediate condition between the tridactyl manus of *Shuvuuia* and monodactyl manus of *Linhenykus*. This highlights a previously unrecognized variation in specialization of alvarezsaurid manus. Notably, the preserved posture of the specimen exhibits a stereotypical avian-like sleeping position seen in the troodontids *Mei* and *Sinornithoides*. Evidence of this behavior in the alvarezsaur *Jaculinykus* suggests that stereotypically avian sleeping postures are a maniraptoran synapomorphy, providing more evidence of bird-like traits being distributed broadly among avian ancestors.

## Introduction

Alvarezsauria is a group of bizarre maniraptoran theropods whose latest-branching members possess remarkably avian homoplasies such as a lightly built, kinetic skull, a keeled sternum, a fused carpometacarpus, and a retroverted pubis and ischium, and underwent clade-specific body size miniaturization during their evolutionary history in their body size [1–6]. Alvarezsaurs with unusual features have received much attention in terms of functional morphology and evolution [6–20]. The derived members during the Late Cretaceous possess unique and shortened forelimbs in having a short, robust humerus with an enlarged deltopectoral crest, an ulna with a greatly enlarged olecranon process, and a hypertrophied manual digit I [1–3,21,22]. Their specialized forelimb resembles those of extant fossorial animals [10,23], which would be used for feeding on colonial insects or eggs [20,24]. The latest-branching members from Asia and North America are also characterized by slender hind limbs with their distal elements much more elongated than the proximal ones and an arctometatarsalian foot, implying their high locomotor efficiency and cursoriality [5,15,25,26]. A recent study further revealed visual and auditory specialization in alvarezsaurs, providing their behavioral insight into nocturnality [9].

**Fig 1:**
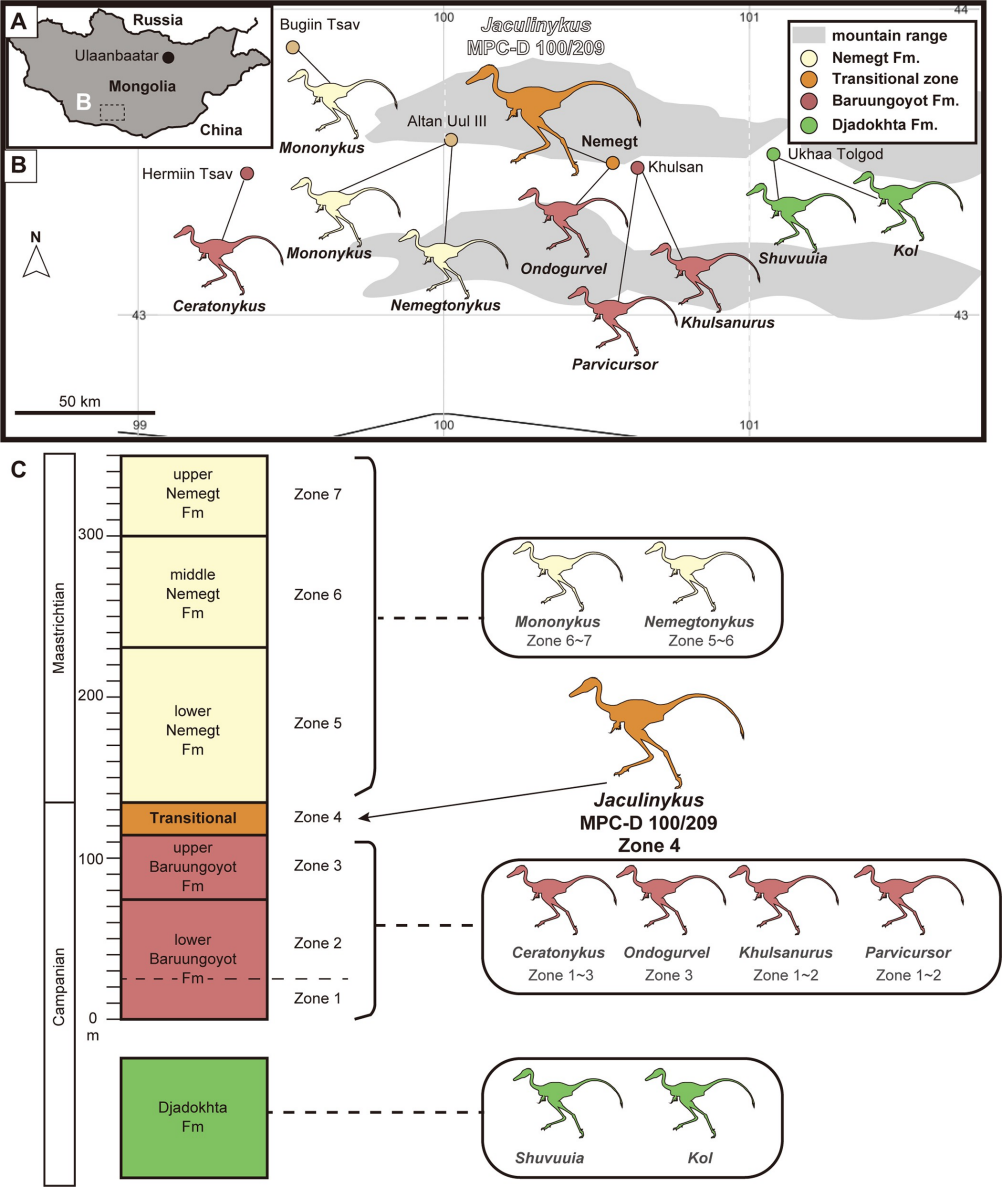
Geographic and stratigraphic occurrence of the Mongolian alvarezsaurids. (**A**) Plan view of Mongolia. (**B**) Location of *Jaculinykus yaruui* gen. et sp. nov. (MPC-D 100/209) and alvarezsaurid occurrences in the Nemegt Basin. The map was generated using Simplemappr (www.simplemappr.net) before modification and modified from Jerzykiewicz et al. [35]. (**C**) The stratigraphic horizon of *Jaculinykus yaruui* gen. et sp. nov. and other alvarezsaurids in the Nemegt, Baruungoyot, and Djadokhta formations. The composite stratigraphy was modified from Eberth [36] and Jerzykiewicz and Russell [37].

The fossil record of alvarezsaurs is globally widespread but known mostly from either Asia or South America. Especially, fossil remains of late-branching alvarezsaurids are abundant in the Nemegt Basin of Mongolia and eight genera have been documented so far (Fig 1). These include *Shuvuuia deserti* [3,22] and *Kol ghuva* [27] from the Djadokhta Formation, *Parvicursor remotus* [28,29], *Ceratonykus oculatus* [30], *Ondogurvel alfanovi* [31], and *Khulsanurus magnificus* [32] from the Baruungoyot Formation, and *Mononykus olecranus* [2,33] and *Nemegtonykus citus* [34] from the Nemegt Formation. Despite the recent increase in the fossil record of alvarezsaurids, a detailed anatomy of the members is still limited due in parts that most of the fossil remains are fragmentary. This often leads to difficulty in interpreting of their ecology and poorly resolved phylogenetic interrelationships [16].

**Fig 2:**
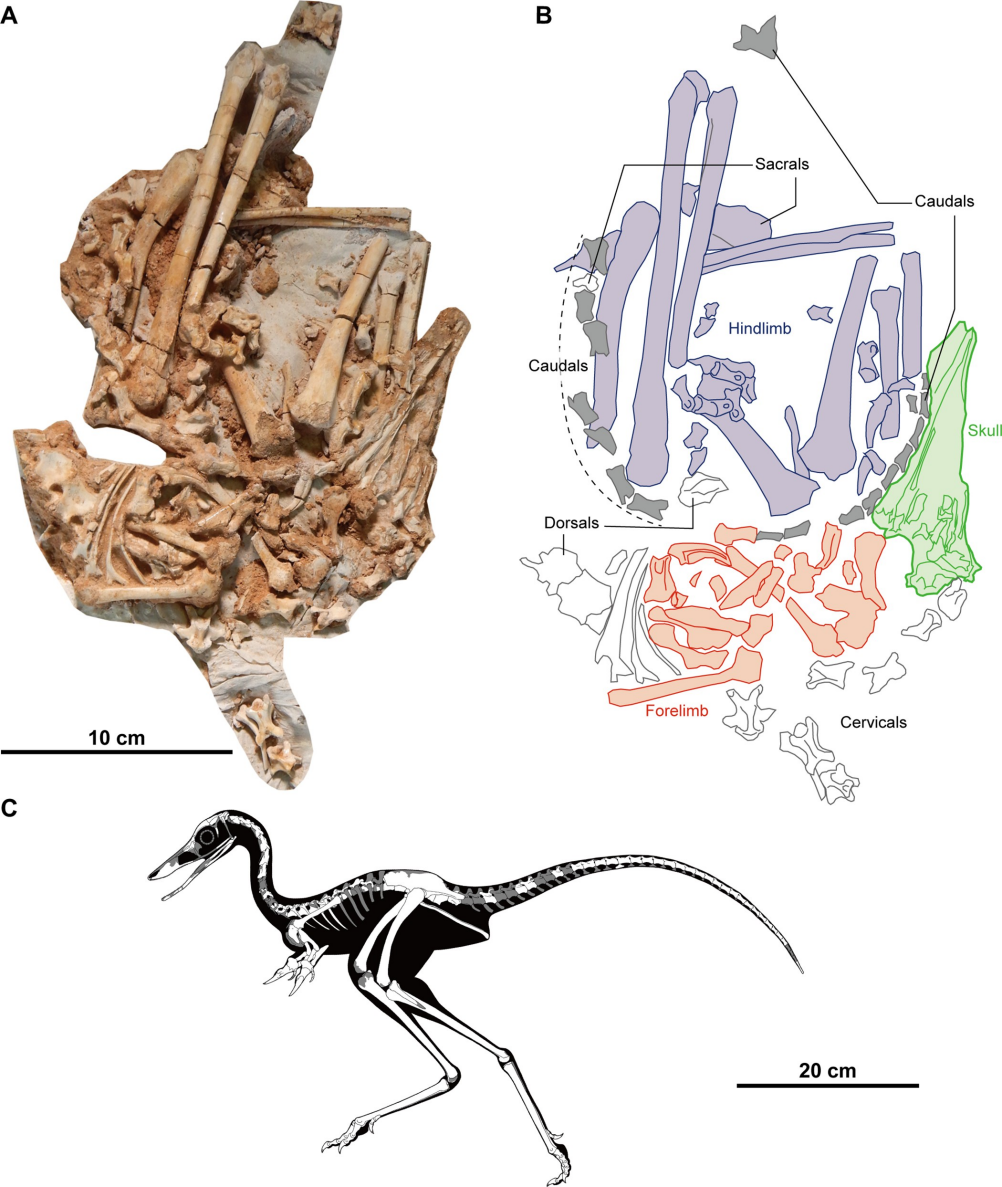
Holotype of *Jaculinykus yaruui* gen. et sp. nov. (MPC-D 100/209). (**A**) Photograph of the specimen. (**B**) Explanatory drawing of (A). Highlighted areas refer to the indication of the skeletal elements; skull in green, tail in grey, pectoral girdle and forelimbs in red, pelvis and hind limbs in purple. (**C**) Reconstruction of *Jaculinykus yaruui* gen. et sp. nov. Grey areas are missing parts.

Here we report the nearly complete and articulated alvarezsaurid skeleton, including the skull, from the Baruungoyot-Nemegt interfingering interval at the Nemegt locality of the Nemegt Basin in the Gobi Desert of Mongolia (Figs 1 and 2). This specimen displays a stereotypical avian-like sleeping position with the neck and tail arched as well as hind limbs folded under the pelvis (Fig 2A and 2B), which is nearly identical to that seen in the troodontids *Mei long* and *Sinornthoides youngi*[38–41]. The new taxon, *Jaculinykus yaruui* gen. et sp., represents the ninth genus of alvarezsaurids from the Nemegt Basin, and unveils not only the comprehensive anatomy of alvarezsaurids, but also provides definitive evidence of the appearance of avian-like behavior long before paravians.

## Materials and methods

The specimen was recovered from the upper section of the Baruungoyot (or Barun Goyot) Formation (Campanian?) [37] in Nemegt locality [35,36,42], Ömnögovi Province, Mongolia (Fig 1). Paleontological fieldwork was conducted under the necessary permits, issued by the Institute of Paleontology of the Mongolian Academy of Sciences (IP-MAS), Ulaanbaatar, Mongolia. The specimen is cataloged under the number MPC-D 100/209. Precise GPS coordinates of the site are accessioned with MPC-D 100/209 at the IP-MAS, where they are available upon request.

The phylogenetic position of the new specimen was assessed by incorporating it into a comprehensive data matrix for the Alvarezsauria [11,17,34]. The data set of Averianov and Sues [17] was modified by adding two new characters (see S1 Appendix). The matrix consists of 118 taxa with 596 characters, where 56 characters were treated as ordered following previous studies [11,34]. In this analysis, *Shishugounykus inexpectus* was removed prior to analysis because it is a wild card taxon. In the phylogenetic analysis using the software TNT v. 1.5 [43], the maximum number of trees was set to 99,999 in memory, and a traditional search was performed with 10,000 replicates of Wagner trees using random addition sequences, followed by the TBR branch swapping that held 10 trees per replicate. Supports for the clade were evaluated by the Bremer support values using the ‘Bremer.run’ script provided by TNT [43].

### Nomenclatural acts

The electronic edition of this article conforms to the requirements of the amended International Code of Zoological Nomenclature, and hence the new names contained herein are available under that Code from the electronic edition of this article. This published work and the nomenclatural acts it contains have been registered in ZooBank, the online registration system for the ICZN. The ZooBank LSIDs (Life Science Identifiers) can be resolved and the associated information viewed through any standard web browser by appending the LSID to the prefix http://zoobank.org/. The LSID for this publication is: urn:lsid:zoobank.org:pub:5E201C9F-3BF0-43D1-9D17-8C64293E8F99. The electronic edition of this work was published in a journal with an ISSN, has been archived, and is available from the following digital repositories: PubMed Central, LOCKSS.

### Institutional abbreviations

**HIII**, Henan Geological Museum, Zhengzhou, China; **IVPP,** Institute of Vertebrate Paleontology and Paleoanthropology, Beijing, China; **MPC-D (= GIN, IGM, MGI, MPD)**, Institute of Paleontology of Mongolian Academy of Sciences, Ulaanbaatar, Mongolia; **HGM**, Henan Geological Museum, Zhengzhou, China; **UCMP,** University of California Museum of Paleontology, USA; **XMDFEC,** Xixia Museum of Dinosaur Fossil Eggs of China; **ZIN PH,** Paleontological Collection, Zoological Institute, Russian Academy of Science, Saint Petersburg, Russia.

Specimens of *Shuvuuia deserti* were originally cataloged MGI, IGM or MPD [3,9,22], but we treated them as MPC-D for the unification of the catalog number.

## Results

### Systematic paleontology

Dinosauria Owen, 1842 [44]

Theropoda Marsh, 1882 [45]

Maniraptora sensu Gauthier, 1986 [46]

Alvarezsauria Bonaparte, 1991 [47]

Alvarezsauridae Bonaparte, 1991 [47]

Parvicursorinae Karhu and Rautian, 1996 [28]

*Jaculinykus yaruui* gen. et sp. nov.

LSID for the genus: urn:lsid:zoobank.org:act: b9b66dfe-9dfb-4f26-8526-807771bfc693

LSID for the species: urn:lsid:zoobank.org:act: 97410fda-1381-4e69-ae69-95ee9b5316eb

Etymology

*Jaculinykus* is from “Jaculus,” a tiny dragon from the Greek myth, and “onykus,” claw; *yaruui*, derived from Mongolian word, yaruu (яаруу): speedy (= hasty).

#### Holotype

The type specimen (MPC-D 100/209) is a nearly complete skeleton with a skull, missing some cranial elements (vomers, nasals, postorbitals, and supraoccipitals), eighth or ninth cervical vertebra, posterior dorsal vertebrae, seven anterior caudal vertebrae, sternum, furcula, right manual phalanx (II-2), right manual ungual and left fibula (Fig 2). It is housed in the Institute of Paleontology of Mongolian Academy of Sciences (IP-MAS), Ulaanbaatar, Mongolia.

#### Type locality and horizon

Nemegt locality [35,36,42], Ömnögovi Province, Mongolia (Fig 1). The specimen was collected from the upper section of the Baruungoyot (or Barun Goyot) Formation, suggested as the Campanian in age, (Fig 1). The stratigraphic horizon of *Jaculinykus* belongs to a part of “Big Red” in the transitional stratigraphic interval (Zone 4), [36,42].

#### Diagnosis

*Jaculinykus* differs from all other alvarezsaurs in having a dorsoventrally high narial opening of the premaxilla, medially curved parasagittal crest on the parietal, slender and nearly straight dentaries, triangular-shaped deltopectoral crest being separated from the humeral head by a notch, strong medial tab of metacarpal I, weakly developed proximodorsal process of phalanx I-1, robust medial condyle of the tibia relative to the fibular condyle, and sharply indented base of the ascending process of astragalus. *Jaculinykus* is also distinguished from other alvarezsaurs by the unique combination of the following characters: slender ischial shaft relative to the pubic shaft, an open popliteal fossa of the femur, and prominent external projection of the ectocondylar tuber of the femur. It differs from the hypothesized sister taxon *Shuvuuia deserti* in possessing the following additional features: the deltopectoral crest being separated from the humeral head by a notch; the absence of the third manual digit. It also differs from stratigraphically same or older (the Baruungoyot Formation) alvarezsaurid, *Ondogurvel alifanovi*, from the same locality (the Nemegt locality) in possessing the following features in the manus: the metacarpal II being not incorporated into the fused metacarpal element, a sharply truncated metacarpal III, and a weakly-developed proximodorsal process of phalanx I-1. It differs from *Parvicursor remotus* of the Baruungoyot Formation in possessing the following features: a straight posterior margin of the medial distal condyle of femur, ectocondylar tuber on the lateral distal condyle not extended posteriorly beyond the level of the medial condyle, and robust and rounded medial condyle of the proximal end of tibia. Differs from *Ceratonykus oculatus* of the Baruungoyot Formation in possessing the following features: the ratio of the frontal length to the transverse width in *Jaculinykus yaruui* being smaller (1.7) than that in *Ceratonykus oculatus* (4.0); a well-developed cnemial crest of the tibia. It differs from *Khulsanurus magnificus* of the Baruungoyot Formation in possessing the following features: the presence of pleurocoels and epipophyses on the cervicals, dorsoventrally thin and subcircular in cross-section transverse process of the anterior caudals, and a notch between the humeral head and deltopectoral crest. It differs from stratigraphically younger (the Nemegt Formation) alvarezsaurid *Mononykus olecranus* in possessing the following features: the femoral head being more robust in *Jaculinykus yaruui* than that in *Mononykus olecranus*, a popliteal fossa on the distal end of the femur opening distally, and an anteriorly oriented cnemial crest of the tibia. It differs from *Nemegtonykus citus* of the Nemegt Formation in possessing the following features: the scapula unfused to the coracoid, a strongly curved femoral shaft, and the presence of an ectocondylar tuber on the femoral distal condyle.

### Description

The three-dimensionally preserved skeleton of *Jaculinykus yaruui* is compressed dorsoventrally, but most elements of the skeleton are positioned nearly at the original position, despite some lateral displacement of some elements (Fig 2A and 2B). The anterior half of the skeleton is oriented ventral-side-up from the skull through the dorsal vertebrae, whereas the posterior half including pelvis and hind limbs oriented left-lateral-side-up. The hind limbs are folded on either side of the body as in *Albinykus bataar* [5]. The left forelimb is extended laterally and folded next to the body with the elbow, although the left manual elements are displaced. The neck curves posteriorly on the right side of the body, so that the skull lies on the right side of the body, above the right knee. Most of the tail lies on the left side of the body. The proximal end of the tail is directed to the left side of the body. The middle to distal parts of the tail are directed anteriorly and curve around the flexed hind limbs to the right, and then travel forward under the skull. This posture is nearly identical to the inferred sleeping posture of troodontids such as the type specimen of *Sinornithoides youngi*, IVPP V9612 [40,41] and *Mei long* [38,39].

The basic measurements of the specimen are provided in S1 Table.

#### Skull

**Fig 3:**
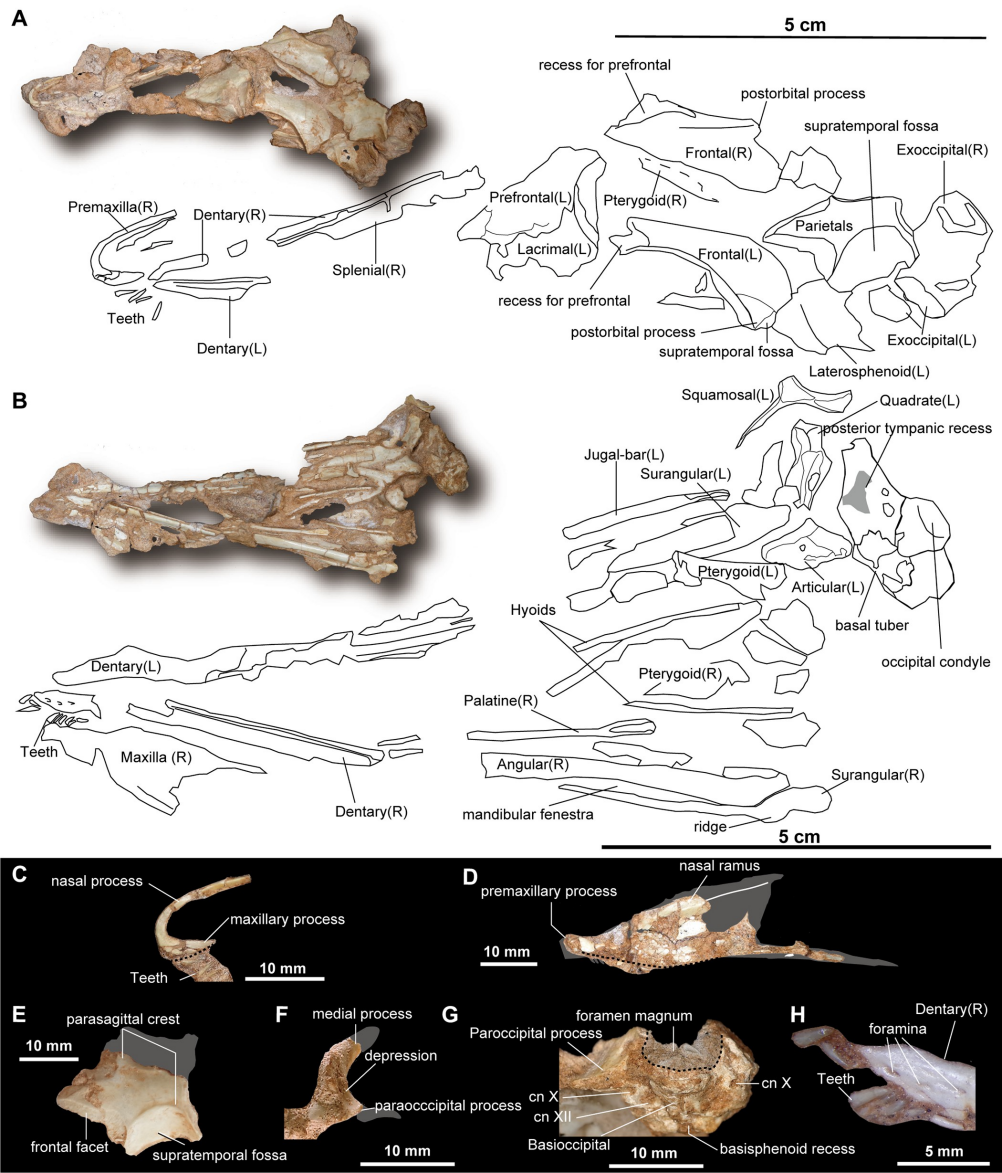
Skull elements of *Jaculinykus yaruui* gen. et sp. nov. (MPC-D 100/209). Skull in dorsal (**A**) and ventral (**B**) views. (**C)** Right premaxilla in medial view (a broken line shows a ventral margin of the premaxilla). (**D**) Right maxilla in lateral view (reversed) (a broken line shows a ventral margin of the maxilla). (**E**) Parietals in dorsal view. (**F**) Left squamosal in dorsal view. (**G**) Braincase elements in posterior view (a broken line and shadow area represent an outline of the foramen magnum). (**H**) Teeth close to the right dentary. Gray-shaded areas indicate missing parts.

The skull of *Jaculinykus yaruui* (Fig 3A and 3B) is dorsoventrally crushed. All cranial elements are displaced from their original positions to some degree.

The premaxilla is anteroposteriorly longer than high and has elongated nasal and short maxillary processes (Fig 3A and 3C). The nasal process forms the dorsal border of the elliptical external nares as in *Shuvuuia deserti* [1,3]. The anterodorsal margin of the nasal process is moderately curved unlike that of *Shuvuuia deserti*, which is straight. The horizontally oriented maxillary process forms a dorsoventrally wide narial opening in comparison to the narial opening of *Shuvuuia deserti*.

Although the right maxilla is severely broken into small pieces, it remains in the original position, allowing observation of some anatomical features. The maxilla has an elongated, roughly triangular outline in the lateral view (Fig 3D). The nasal ramus of the maxilla has a shallow sulcus along its dorsal rim as in *Shuvuuia deserti* (MPC-D 100/977, [1,3]). The ventral margin of the premaxillary process also slightly slants anterodorsally as in *Shuvuuia deserti* but unlike the non-alvarezsaurid alvarezsauroid *Haplocheirus sollers*, which has a straight ventral margin of the maxilla. Six maxillary teeth are aligned along the ventral margin of the premaxillary process.

The prefrontal is a large, rhomboidal cranial element with a sharp posterior apex in dorsal view as in *Shuvuuia deserti* [3] and *Ceratonykus oculatus* [30], (Fig 3A). The prefrontal overlaps the frontal ventromedially, which is unique to alvarezsaurids [1]. In addition, the anterolateral surface of the prefrontal lacks a concave notch for articulation with the lacrimal angle unlike non-alvarezsaurid alvarezsauroids such as *Haplocheirus sollers* [12].

The frontal forms the entire dorsal margin of the orbit (Fig 3A). It is slightly vaulted dorsally as in alvarezsaurids [1,30], but unlike a flattened skull roof of *Haplocheirus sollers* [12]. Dorsally, the frontal is anteroposteriorly long and becomes transversely narrow towards its anterior end (Fig 3A). Anteriorly, the frontal has a large recess constricting posteriorly for the articulation with the prefrontal. The contact between the frontals is straight without complex interdigitation, as in other alvarezsauroids [1,12]. A distinct orbital rim develops along the lateral rim of the frontal from the prefrontal facet to the postorbital process as in *Shuvuuia deserti* [1,3], troodontids [48], and some dromaeosaurids (e.g., *Mahakala omnogovae* [49]). The postorbital process of the frontal is anteroposteriorly short and laterally extensive, which is weaker than that in *Haplocheirus sollers*. There is a weak and small recess of the anterior margin of the supratemporal fossa on the postorbital process as in *Shuvuuia deserti*, but unlike early-branching alvarezsauroids in which the supratemporal fossa occupies a large portion of the frontal [8,11].

The left and right parietals are completely fused in *Jaculinykus yaruui* as in *Shuvuuia deserti* [1], (Fig 3A and 3E). Medially, the fused parietals are horizontally flat without a sagittal crest and forms the posterior part of the skull roof. Lateral to the distinct parasagittal crests, the lateral surface and posterior process of the parietal slant ventrolaterally with a shallow angle, forming the medial margin of the supratemporal fossa (Fig 3E). Parasagittal crests are present on the dorsal surface of the parietals, and are strongly curved, unlike the straight parasagittal crests of *Shuvuuia deserti* (MPC-D 100/977). These crests extend more medially in *Jaculinykus yaruui*, suggesting that the supratemporal fossa is larger than that of *Shuvuuia deserti*.

The squamosal possesses postorbital, medial, and paraoccipital processes (Fig 3F). The features of these three processes in *Jaculinykus yaruui* are ambiguated because each process is either missing distally or covered by other cranial elements. The squamosal lacks the ventral process, forming a triradiate element as in *Shuvuuia deserti* [1,3] and *Ceratonykus oculatus* [30]. Ventrally, the paraoccipital process has a shallow furrow articulating with the quadrate heads. The dorsal surface of the squamosal has a more distinct depression around the junction between the medial and paraoccipital processes than that of *Shuvuuia deserti* (MPC-D 100/977).

Lateral to the prefrontal, the main body of the left lacrimal is preserved in articulation with the prefrontal (Fig 3A). Although the maxillary and jugal processes of this element are heavily damaged, it forms an inverted ‘L’-shaped outline. The lacrimal lacks a prominent posterior process. Unlike *Shuvuuia deserti* (MPC-D 100/977), *Jaculinykus yaruui* does not bear a distinct sutural contact between the maxillary process and the prefrontal.

Although the maxillary process and anterior part of the jugal are missing, the preserved parts clearly show that the jugal is fused to the quadratojugal and together forms the rod-like jugal bar as in *Shuvuuia deserti* [1,3]. The jugal bar is circular in cross-section and weakly curved mediolaterally. Posteriorly, the jugal bar lacks the postorbital and squamosal processes (Fig 3B). The posterior end of the jugal bar has a shallowly concave facet for articulation with the quadrate.

The quadrate is tall and compressed anteroposteriorly. The quadrate is shallowly excavated posteriorly (Fig 3B) as seen in *Shuvuuia deserti* [1,3]. Although both tips of quadrate heads are missing, the lateral quadrate head extends along the lateral flange. Ventral to the base of the medial quadrate head, there is a distinct oval quadrate foramen at the mid-posterior surface of the quadrate as in *Shuvuuia deserti* (MPC-D 100/1001).

The exposed width of the foramen magnum relative to the occipital condyle (Fig 3G) suggests that its size is comparable to that of *Shuvuuia deserti* (MPC-D 100/1001, 100/1304), [1,3], which is known for its unusually large foramen magnum. The occipital condyle is transversely wider than dorsoventrally high, with an infracondylar recess in its ventral surface. Ventral to the occipital condyle, the basal tuber (Fig 3B) is well-developed and transversely wide. The subcondylar recess, which extends from the exoccipital, is more deeply excavated on the posterodorsal surface of the basal tubera than the exoccipital. The basal tubera are separated along the midline of the skull, continuing into the basisphenoid recess, as seen in *Haplocheirus sollers* and *Shuvuuia deserti* [12]. This gap between the basal tubera is proportionally narrower than that in *Shuvuuia deserti*.

The paroccipital process is short and slightly expanded distally (Fig 3G). In posterior view, the paraoccipital process extends laterally as in *Shuvuuia deserti* (MPC-D 100/1304), but unlike the paroccipital process in an unnamed alvarezsaurid (HGM L08-59) from China which is oriented lateroventrally [16]. Two foramina for the vagus (X) nerve are present on each side of the exoccipital and at the same level as the paroccipital process (Fig 3G). Medial to the foramen of the vagus (X) nerve, the hypoglossal (XII) foramen is present near the base of the occipital condyle. In ventral view, the anterior surface of the exoccipital bears a large posterior tympanic recess (Fig 3B).

The palate elements (pterygoid and palatine) are partially preserved. Although both anterior parts of the pterygoids are broken partially or covered by matrix and other cranial elements, the pterygoid is anteroposteriorly long and straplike as in *Shuvuuia deserti* (MPC-D 100/1001). Posteriorly, the pterygoid has a lateral ramus for articulation with the quadrate (Fig 3B). The posterior portion of the palatine is long and slender. The posterior end of the palatine is slightly expanded transversely compared to its shaft, forming a flat and thin articular facet for the pterygoid (Fig 3B).

The lateral curve of the dentary toward its posterior end is weak compared to *Shuvuuia deserti* (MPC-D 100/977 and 100/120), (Fig 3B). The ratio of the posterior width of the dentaries to its anteroposterior length is slightly smaller (9.5%) than that in *Shuvuuia deserti* (10.5%), (MPC-D 100/120, [22]). Anteriorly, the foramina are present on the lateral surface of the dentary and along the symphysis. The unfused symphysis is slightly inflected medially, forming a V-shaped symphysis in ventral view (Fig 3B and 3H). Posteriorly, there is a long alveolar groove along the dorsal border of the dentary, despite no observation of the teeth in the preserved alveolar groove because of a matrix. The splenial and dentary meet each other along a straight suture.

The surangular is anteroposteriorly long and dorsoventrally low (Fig 3B). The long anterior process composes more than half of the entire length of the surangular. It possesses a long groove on its lateral surface in the anterior half part and forms the dorsal border of the external mandibular fenestra (Fig 3B). The anterior process of the surangular does not extend anteriorly beyond the level of the anterior end of the angular as in *Shuvuuia deserti*. The surangular becomes low and transversely widens posteriorly towards a lateral ridge (Fig 3B). Posterior to the lateral ridge, the surangular overlaps the articular laterally and the angular dorsally.

The angular is weakly bowed ventrally and covers the entire length of the external mandibular fenestra ventrally (Fig 3B). The angular meets the prearticular posteriorly and the surangular ventrolaterally. Although most parts of the retroarticular process of the articular is missing, a subcircular basin is present in it. The posterior half of both hyoids is straight relative to those of *Shuvuuia deserti* (MPC-D 100/977) and has a ridge for the attachment of the lingual muscle.

Twenty-eight tiny teeth (Figs 3H and S1) are recovered. The teeth are not *in situ* but are preserved close to the premaxilla, maxilla, and dentary. Since all teeth are associated near the original position and are projecting the same orientation, these teeth likely retain the original relationships. The teeth are homodontous, ranging from 1 mm and 5 mm in size. Each tooth crown exhibits a subconical shape and lacks serrations as in *Mononykus olecranus* [2,33], *Shuvuuia deserti* [1], and some troodontids [48], but unlike early-branching alvarezsauroids (e.g., *Haplocheirus sollers*) which retain denticulate tooth crowns [12].

### Postcranial skeleton

#### Vertebral series

The cervical series is nearly complete in *Jaculinykus yaruui*, preserving nine out of twelve cervical vertebrae present in late-branching alvarezsaurids, including *Shuvuuia deserti* (MPC-D 100/120 and 100/977). The missing cervical elements are the atlas, the eighth and tenth cervical vertebra. In contrast to the amphi- to platycoelous centra in *Haplocheirus sollers* [50,51] and amphiplatyan condition in *Alvarezsaurus calvoi* [47], each cervical centrum is opisthocoelous as in late-branching alvarezsaurids [1]. The transition between the cervical and dorsal vertebrae is based on the following features: the position of the parapophyses; the shapes of the transverse process; the shape of the zygapophyses; rib length [52–54]. The first dorsal vertebra differs from the cervical vertebrae in possessing well-developed parapophyses extended laterally beyond the level of the prezygapophyses, a well-developed neural spine, and well-developed ribs.

**Fig 4:**
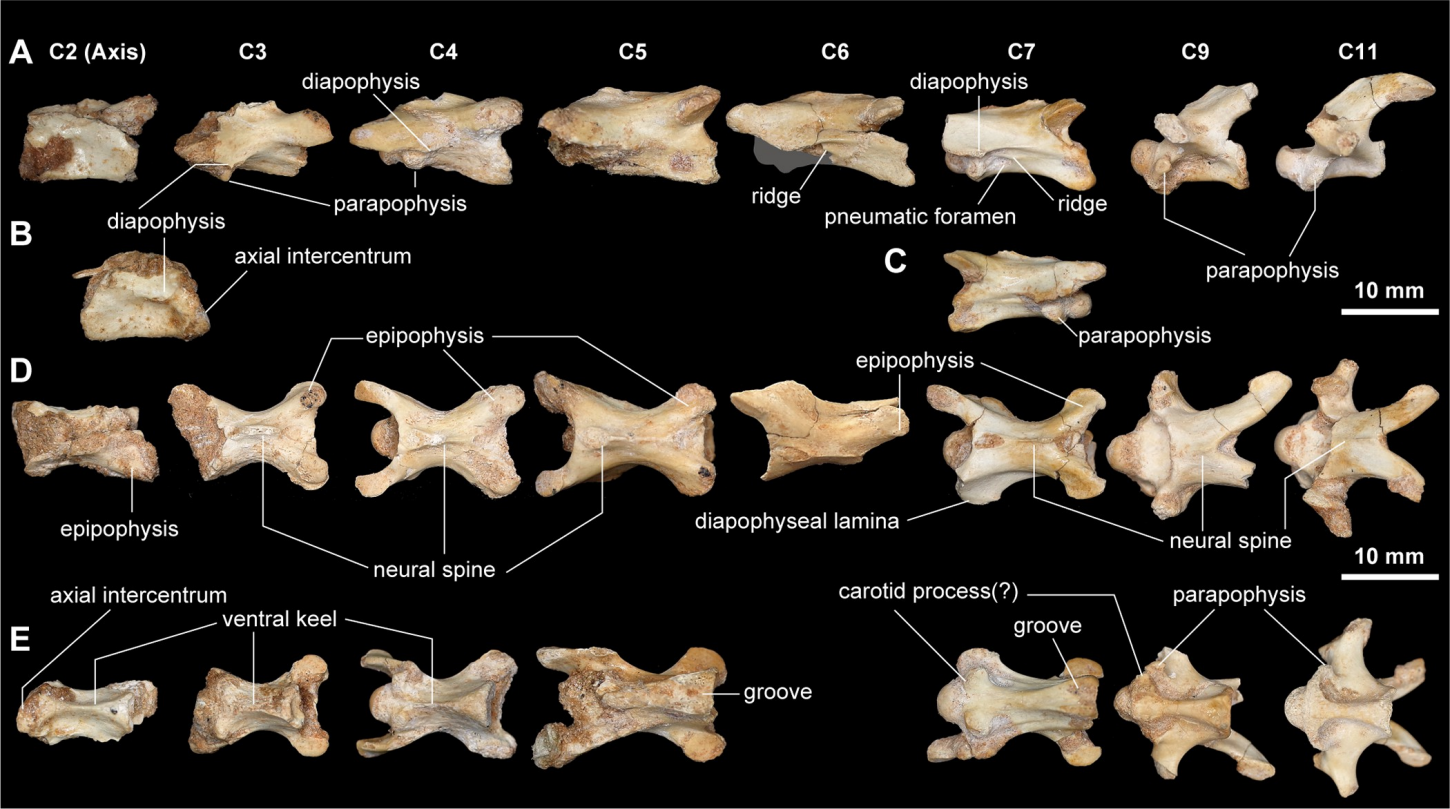
Cervical series of *Jaculinykus yaruui* gen. et sp. nov. (MPC-D 100/209). Cervical series in left lateral (**A**), dorsal (**D**), and ventral (**E**) views. (**B**) Axis in right lateral view. (**C**) Seventh cervical vertebra in right lateral view. The numbers indicate the position of cervical vertebrae. A gray area indicates missing part.

The cervical neural spines become dorsoventrally low and anteroposteriorly elongated posteriorly. Except for the axis (C2) in which the prezygapophysis is short, the prezygapophyses and postzygapophyses are well-developed anteroposteriorly and laterally, making the X-shaped outline in dorsal view (Fig 4D) as in other alvarezsaurids [20]. Epipophyses are present throughout all cervicals and are progressively reduced in size posteriorly. The small diapophysis is present posterodorsal to the parapophyses, which are located on the anterior end of the centrum. The anterior (C2–4) and mid (C5–7) cervical centra become anteroposteriorly longer posteriorly, the seventh cervical vertebra being the longest among the cervical vertebrae (Fig 4). Each centrum is strongly compressed mediolaterally, especially in the anterior cervical vertebrae (Fig 4C), and has a short ridge ventral to the diapophyseal lamina in the sixth and seventh cervical centra as in *Linhenykus monodactylus* [15]. Despite the presence of a subcircular pneumatic foramen posterior to the parapophysis in the seventh cervical vertebrae, the other cervical centra lack it as in *Shuvuuia deserti*, *Alvarezsaurus calvoi*, and *Linhenykus monodactylus* [1,15]. The axial intercentrum lacks a recess ventrally and is aligned with the ventral surface of the axial centrum (Fig 4B and 4E). In ventral view, the centra of the anterior and middle cervicals, except for the axis, are excavated anteriorly by a furrow bordered by prominences comparable to the carotid processes.

**Fig 5:**
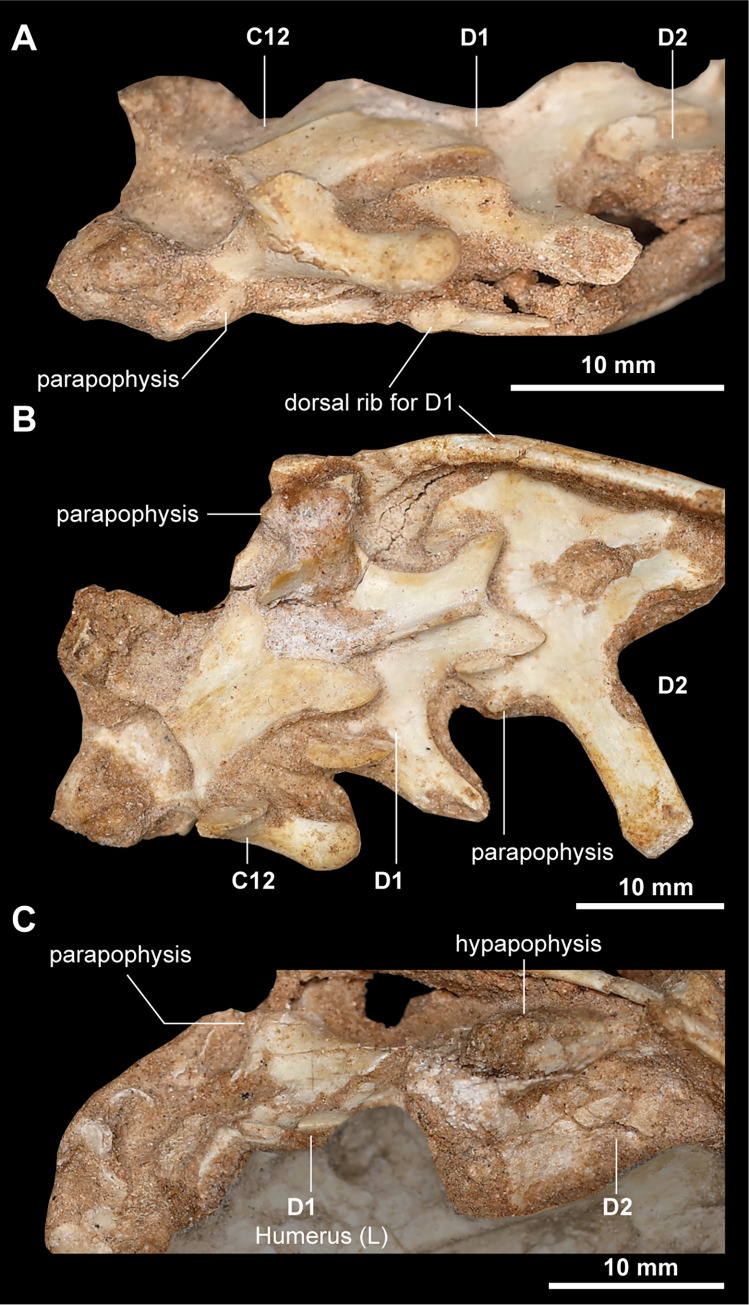
Posterior cervical vertebrae and anterior dorsal vertebra of *Jaculinykus yaruui* gen. et sp. nov. (MPC-D 100/209). Twelfth cervical and first–second dorsal vertebrae in lateral (**A**), dorsal (**B**), and ventral (**C**) views.

**Fig 6:**
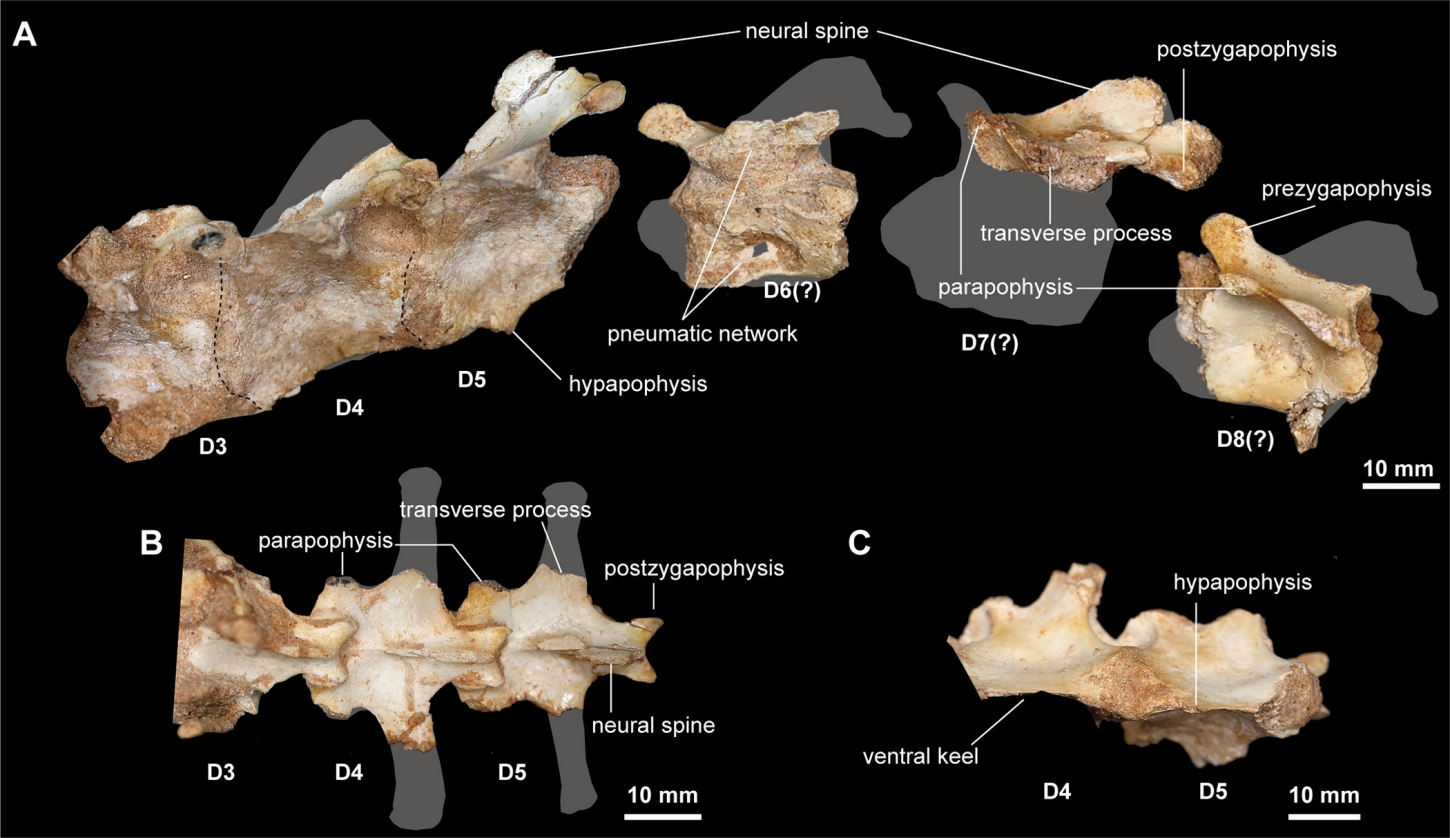
Dorsal vertebrae of *Jaculinykus yaruui* gen. et sp. nov. (MPC-D 100/209). (**A**) Third to fifth dorsal vertebrae (reversed) that are preserved in articulation and isolated posterior dorsal vertebrae in lateral views. Third to fifth dorsal vertebrae in dorsal (**B**) and ventral (**C**) views. The numbers indicate the position of dorsal vertebrae and gray areas indicate missing parts.

Posterior cervical vertebra (C8–12) are nearly equal in anteroposterior length. Unlike the anterior and mid cervicals (C2–7), the neural arches possess long transverse processes that extend posterolaterally beyond the level of those of other cervical centra (Figs 4D and 5B). The neural arches are dorsoventrally high and slant posterodorsally, whereas the neural spine is low as in the anterior and middle cervical vertebrae. The prezygapophyses and postzygapophyses face dorsomedially and ventrolaterally, respectively. The twelfth cervical vertebra further presents differences from the first dorsal vertebra in possessing proportionally large postzygapophyses and the round tip of the transverse processes (Fig 5B). Infrazygapophyseal laminae and pneumatic recesses are well-developed in the posterior cervical centra (C8–12). In ventral view, two prominences (carotid processes) are well-developed on the anterior end of the centrum of the posterior cervicals (C8–12), as in those of the anterior and middle cervicals (C2–7) (Fig 4E). The last cervical (C12) and anterior dorsal vertebrae (D1–5) are greatly compressed transversely (Figs 5C and 6).

Nine dorsal vertebrae are preserved (Figs 5, 6 and S2). Their neural arches overhang laterally and form a shelf-like lamina that joins the prezygapophysis, parapophysis, diapophysis, and postzygapophysis (Fig 6A). The neural spines of the anterior dorsal vertebrae (D1–5) slope somewhat posterodorsally. A hyposphene-hypantrum articulation is absent in all remaining dorsal vertebrae as in alvarezsaurids [1,15,33,55], in contrast with non-alvarezsaurid alvarezsauroids such as *Patagonykus puertai* [21] and *Haplocheirus sollers* [4]. The prezygapophyses and postzygapophyses of the dorsal vertebrae face medially and laterally, respectively (Fig 6B). The parapophyses are present lateral to the bases of the prezygapophyses unlike those of the posterior cervical vertebrae which lie below the neural arches. The transverse process is longer and extends more laterally than those of the posterior cervical vertebrae. All remaining dorsal centra are opisthocoelous and lack pleurocoels, but a pneumatic network is evident inside the neural arch and centrum of the broken sixth dorsal vertebra (Fig 6A). The centra of the anterior dorsal vertebrae of *Jaculinykus yaruui* are dorsoventrally high and strongly compressed transversely, forming a ventral keel, whereas the transverse compression is less substantial in the posterior dorsals as in *Mononykus olecranus* [33]. The transverse compression and the ventral keel of the anterior dorsals are much more developed than those in *Shuvuuia deserti* [3]. The ventral keel of the anterior dorsal vertebrae (D1–5) extends anteriorly into a prominent hypapophysis (Figs 5C and 6). On the other hand, the ventral keels of *Mononykus olecranus* develops only in the first dorsal vertebra [33]. Each posterior articular facet of the anterior dorsals slants posterodorsally.

**Fig 7:**
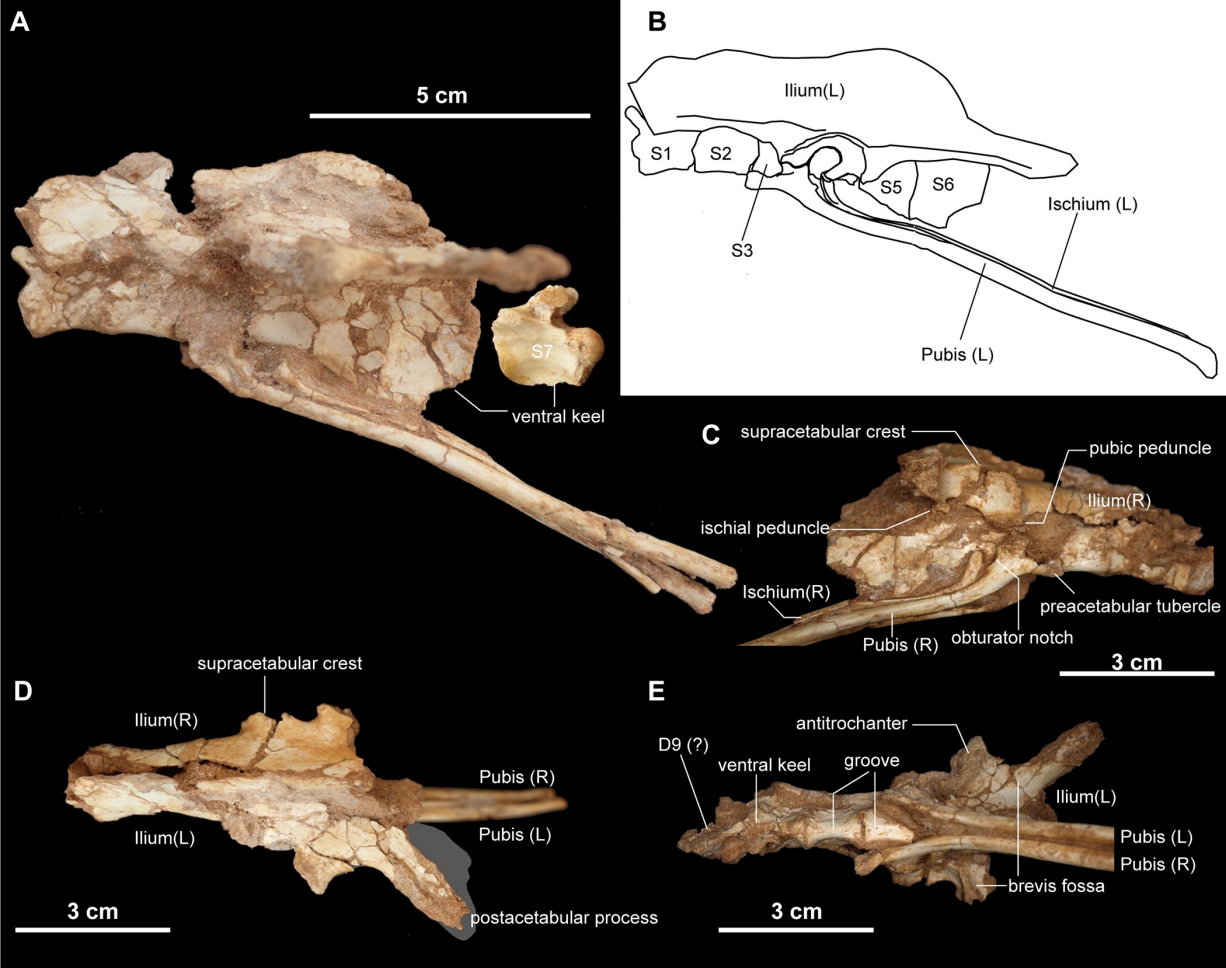
Pelvic girdle and sacral vertebrae of *Jaculinykus yaruui* gen. et sp. nov. (MPC-D 100/209). Pelvic girdle and sacral vertebrae in left lateral (**A**), right ventrolateral (**C**), dorsal (**D**), and ventral (**E**) views. **(B)**, Reconstructed pelvic girdle and sacral vertebrae. Gray area indicates missing part.

There are seven sacral vertebrae (Fig 7) as in alvarezsaurids [1,20,25], in contrast with *Haplocheirus sollers* that has five sacral vertebrae [4]. The centra of first and second, as well as the fifth and sixth sacrals, are fused to one another, respectively (Fig 7A and 7B). The first sacral centrum is strongly compressed transversely, forming a ventral keel in contrast with the second and third centra, which have a flat or shallowly grooved ventral surface. The extreme transverse compression in the fifth to seventh sacrals resembles the other alvarezsaurids [1,25] and *Patagonykus puertai* [21]. The ventral margin of the fifth and sixth sacral centra slant posteroventrally. The neural arch of the seventh sacral does not exceed posteriorly beyond the posterior margin of its centrum. The articular condyle of the first sacral is unknown because of the intervertebral articulation with the last dorsal vertebra, but the seventh sacral centrum is procoelous.

**Fig 8:**
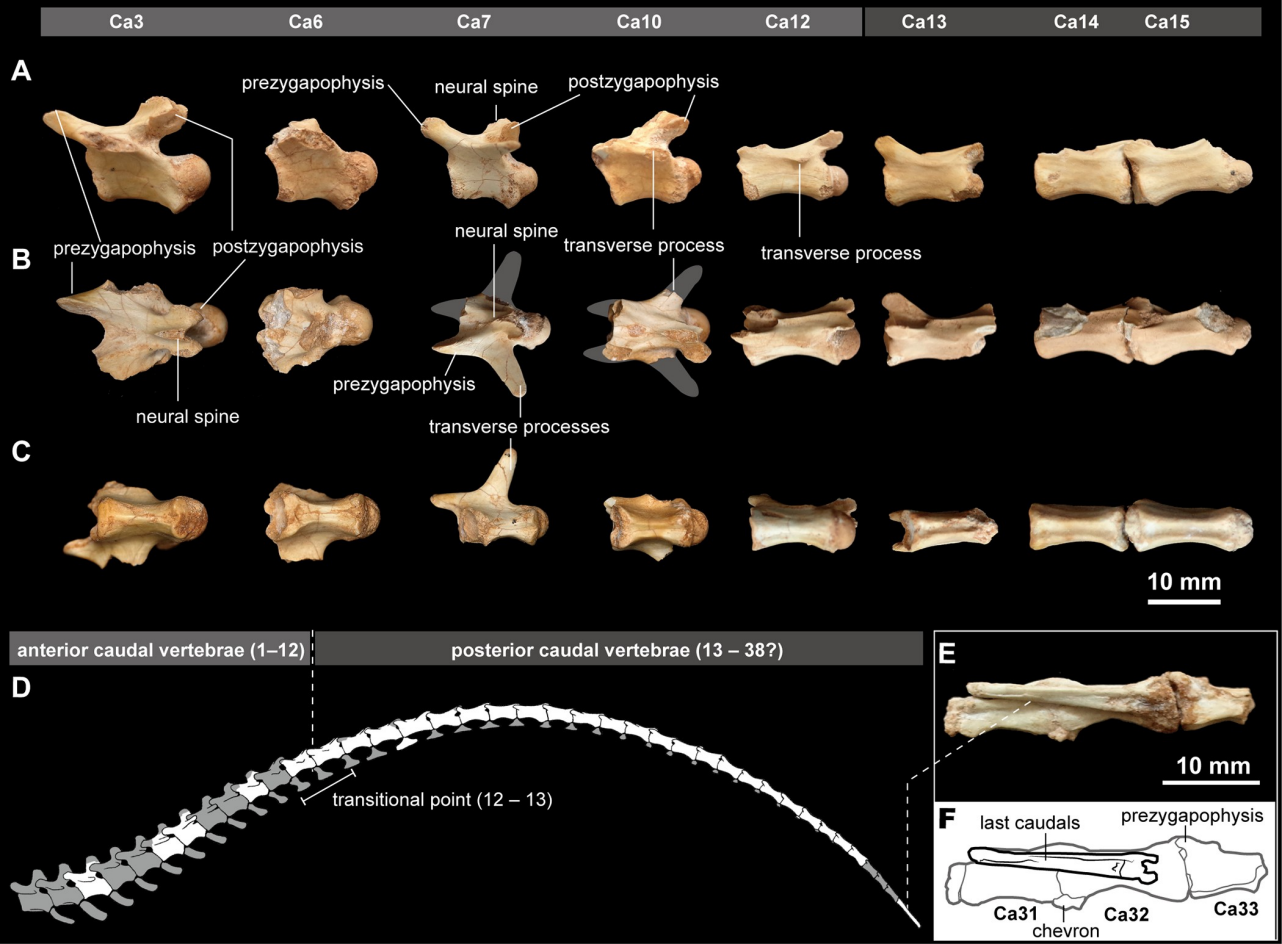
Caudal series of *Jaculinykus yaruui* gen. et sp. nov. (MPC-D 100/209). Caudal vertebrae in lateral (**A**), dorsal (**B**), and ventral (**C**) views. (**D**) Reconstructed tail. (**E–F**) Last caudal elements. The numbers in (A)–(D) and (F) indicate the position of caudal vertebrae. A gray area indicates missing part.

Nearly all caudal vertebrae are preserved. Eight anterior and middle caudal vertebrae as well as the last caudal are collected in isolation, and nineteen caudal vertebrae were preserved in articulation (Figs 8 and S3). The procoelous caudal centra become dorsoventrally lower posteriorly. Each caudal centrum has a longitudinal midline sulcus on the ventral surface, except for the third caudal vertebra, where the narrow ventral surface is flat. The neural spines of the anterior caudals are dorsoventrally high and located on the posterior half of the neural arches. In the anterior caudals, the transverse processes are long and inclined posteriorly in dorsal view (Fig 8B), and reduced to a midline ridge towards the twelfth to thirteenth caudal vertebrae (transitional point), (Fig 8A), as in other alvarezsaurs [13]. Posterior to a transitional point, the neural spines and transverse processes are greatly reduced to a low ridge and located on the midline of the centrum. The prezygapophyses are anterodorsally extended beyond the anterior level of the centrum and reduce size posteriorly. The postzygapophyses of the anterior dorsals are considerably shorter than the prezygapophyses (Fig 8A). In contrast, they are the same in size posterior to the transitional point and extend posterodorsally beyond the posterior level of the centrum (Fig 8E and 8F). The rod-like last caudal element is more than 1.5 times as long in anteroposterior length as other posterior caudal vertebrae (Fig 8E and 8F), which likely consisted of more than two fused centra as in other Maniraptoriformes [56–58].

#### Pectoral girdle

**Fig 9:**
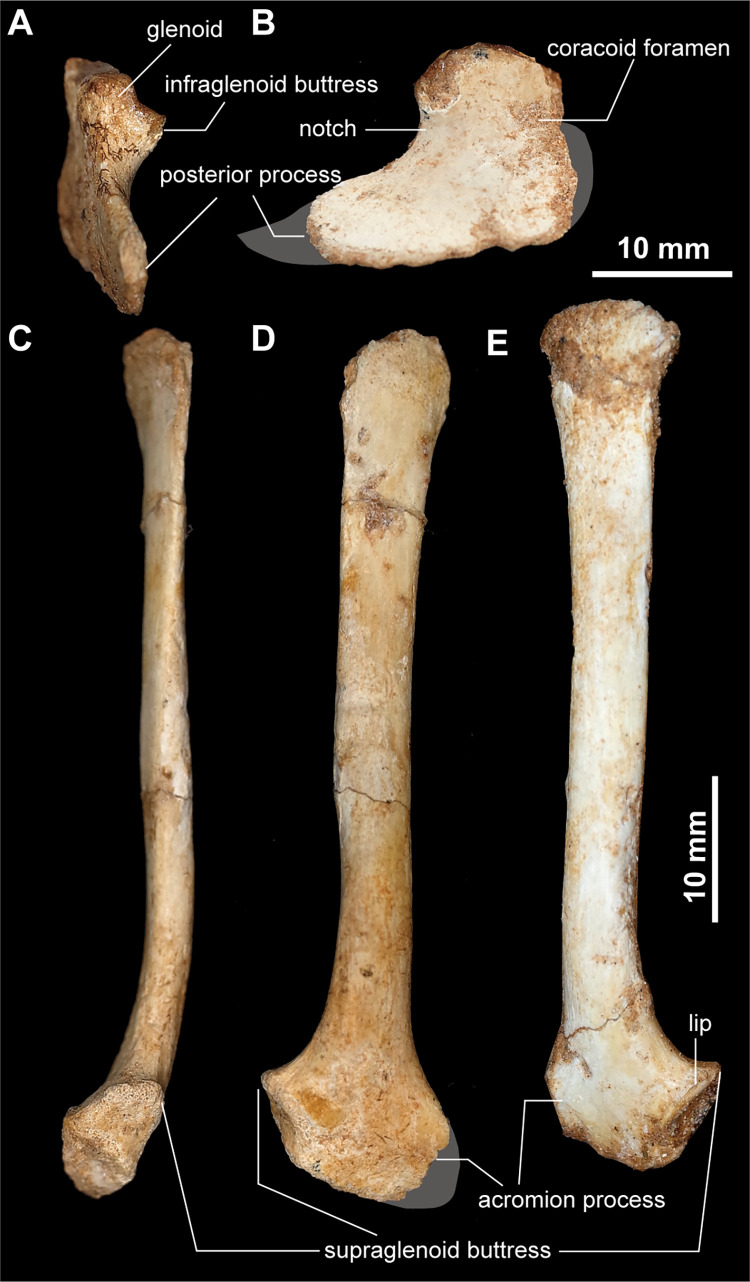
Pectoral girdle of *Jaculinykus yaruui* gen. et sp. nov. (MPC-D 100/209). Right coracoid in ventral (**A**) and lateral (**B**) views. Right scapula in posterior (**C**) and lateral (**D**) views and left scapula in lateral view (**E**). Gray areas indicate missing parts.

The scapula and coracoid are unfused unlike the scapulocoracoids of *Linhenykus monodactylus* [7,15] and *Nemegtonykus citus* [34], (Fig 9). The crescentic coracoid has a long posterior process and glenoid facet, which faces posterolaterally. The infraglenoid buttress (glenoid lip) is well-developed and oriented laterally (Fig 9A). In lateral view, a notch to the infraglenoid buttress in *Jaculinykus yaruui* is weaker than in *Shuvuuia deserti* and *Mononykus olecranus*. Whereas a pyramidal biceps tubercle is present ventral to the glenoid in early-branching alvarezsauroids [4,11], it is absent in *Jaculinykus yaruui* as in alvarezsaurids [1,15,33,34]. The coracoid shaft is flat and thin relative to the region articulated with the scapula, unlike the Patagonian taxa (e.g., *Patagonykus puertai* and *Bonapartenykus ultimus*), which possess a prominent longitudinal ridge [21,59]. The subcircular coracoid foramen is present ventral to the scapular articulation.

The scapular blade is long and straight, and its dorsal and ventral margins are subparallel (Fig 9B and 9C). The proximal end of the scapula abruptly expands dorsoventrally. As in alvarezsaurids, the scapular blade is medially curved proximally in ventral view [1,33,34], (Fig 9C). The acromion process of *Jaculinykus yaruui* extends anteriorly beyond the anterior margin of the scapular blade as in alvarezsaurids [1,34] and *Haplocheirus sollers* [4] but unlike the straight acromial margin of *Xiyunykus pengi* and *Bannykus wulatensis* [11]. The glenoid process is short relative to the acromion process and extends posteriorly nearly perpendicular to the scapular blade (Fig 9A and 9B). The supraglenoid buttress is associated with a low lip on the lateral surface of the scapula (Fig 9C and 9E).

#### Forelimb

**Fig 10:**
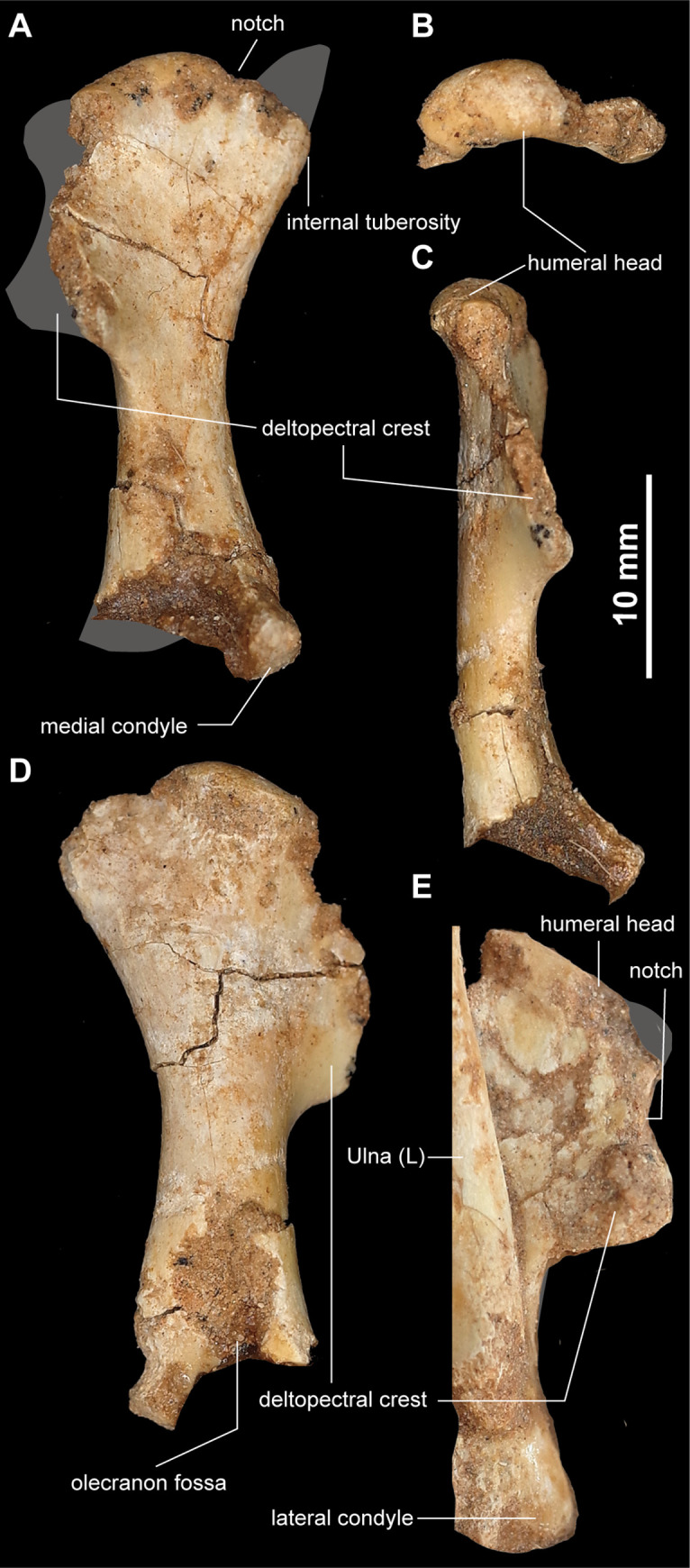
Humeri of *Jaculinykus yaruui* gen. et sp. nov. (MPC-D 100/209). Right humerus in anterior (**A**), proximal (**B**), lateral (**C**), and posterior (**D**) views and left humerus in anterior view (**E**). Gray areas indicate missing parts.

The humerus is proximodistally short and straight, unlike the slender and sigmoidal humerus of *Haplocheirus sollers* [4], (Fig 10). The proximal and distal ends are aligned in the same plane. The large humeral head is anteriorly concave and posteriorly convex as in *Mononykus olecranus* and *Shuvuuia deserti* [1,33]. Although most of the internal tuberosity is missing, the preserved proximal region suggests a similar morphology to other alvarezsaurids, where it is separated from the humeral head by a notch (Fig 10A and 10E). The deltopectoral crest is strongly developed and separated from the humeral head by a notch as in *Mononykus olecranus*, but unlike that in *Shuvuuia deserti* and *Khulsanurus magnificus*, which is continuous with the humeral head [1,3,32]. At the same time, the anterodorsally expanded deltopectoral crest resembles that of *Shuvuuia deserti* but differs from the pillar-like shape of the deltopectoral crest in *Mononykus olecranus*. The distal end of the humerus has two distal condyles as in other alvarezsaurids. In anterior view, the medial condyle is sub-circular and anteriorly expanded. The deep olecranon fossa is developed on the posterior surface, separating the medial and lateral condyles (Fig 10D).

**Fig 11:**
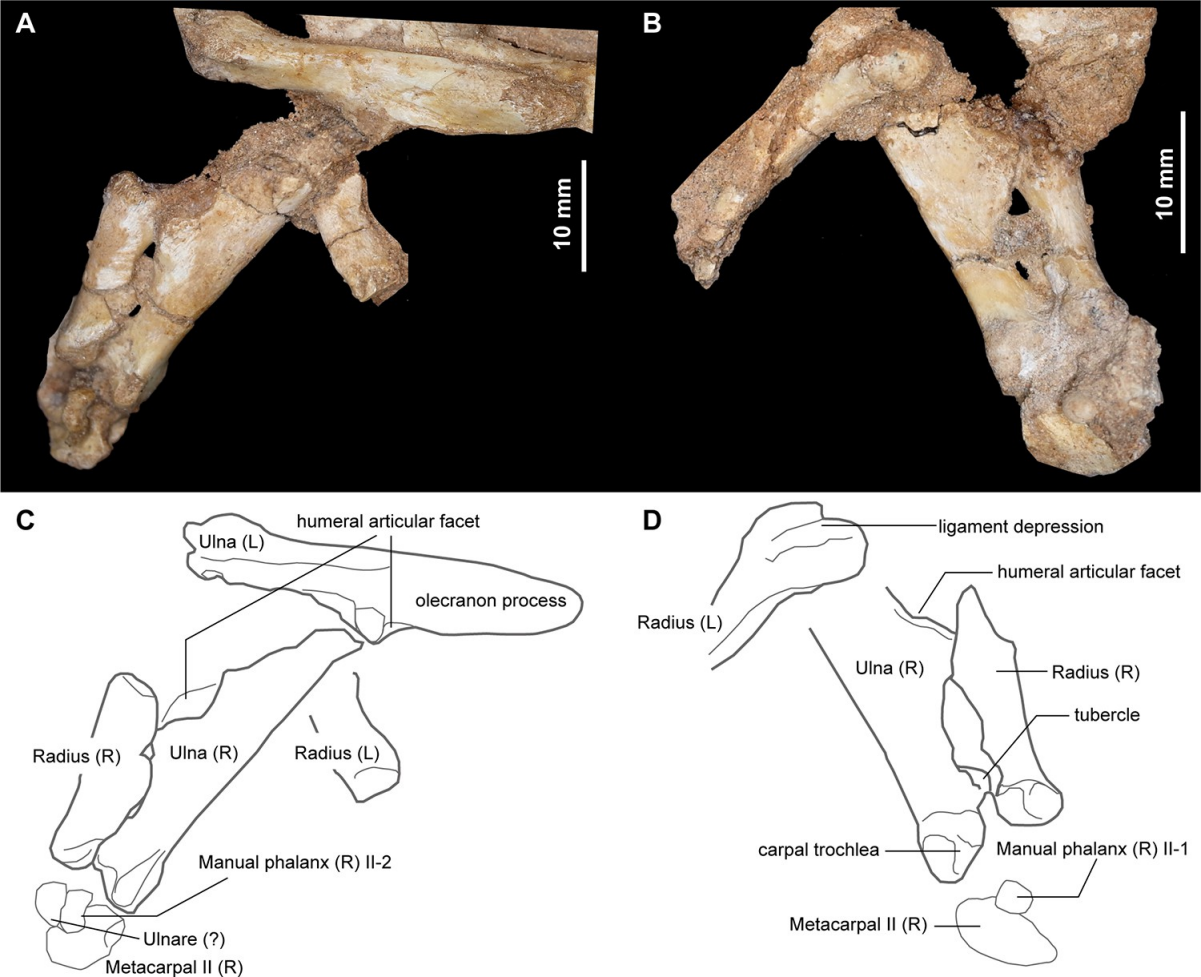
Articulated ulnae and radii of *Jaculinykus yaruui* gen. et sp. nov. (MPC-D 100/209). Photographs (**A**, **B**) and explanatory drawings (**C**, **D**) of the ulnae and radii in ventral (**A, C**) and dorsal (**B, D**) views.

The right ulna and radius are preserved in articulation (Fig 11). As in other alvarezsaurids, the olecranon process is extremely long and occupies nearly half the proximodistal length of the ulna [1,24]. The hypertrophied olecranon process is spur-shaped, as in *Mononykus olecranus* [33], but unlike those in *Albertonykus borealis* and *Patagonykus puertai*, which are a subtriangular [24]. The ulnar shaft is straight and compressed dorsoventrally (Fig 11A and 11B). The humeral articulation of the ulna is oriented proximomedially and continuous with the proximal end of the radius, forming a crescentic articular facet for the humerus. Distally, a prominent tubercle is present on the medial margin of the ulna in dorsal view (Fig 11B), as in *Albertonykus borealis* [24], but unlike that in *Mononykus olecranus*, which bears a small ridge in the ulnar shaft. The distal end of the ulna is subtriangular and has a shallowly grooved carpal trochlea. The radial shaft is also robust and short proximodistally (Fig 11). The distal end of the radius is considerably expanded mediolaterally and has a deep ligamental depression ventrally, as in other alvarezsaurids [1]. This depression is articulated with the tubercle on the medial margin of the ulna.

**Fig 12:**
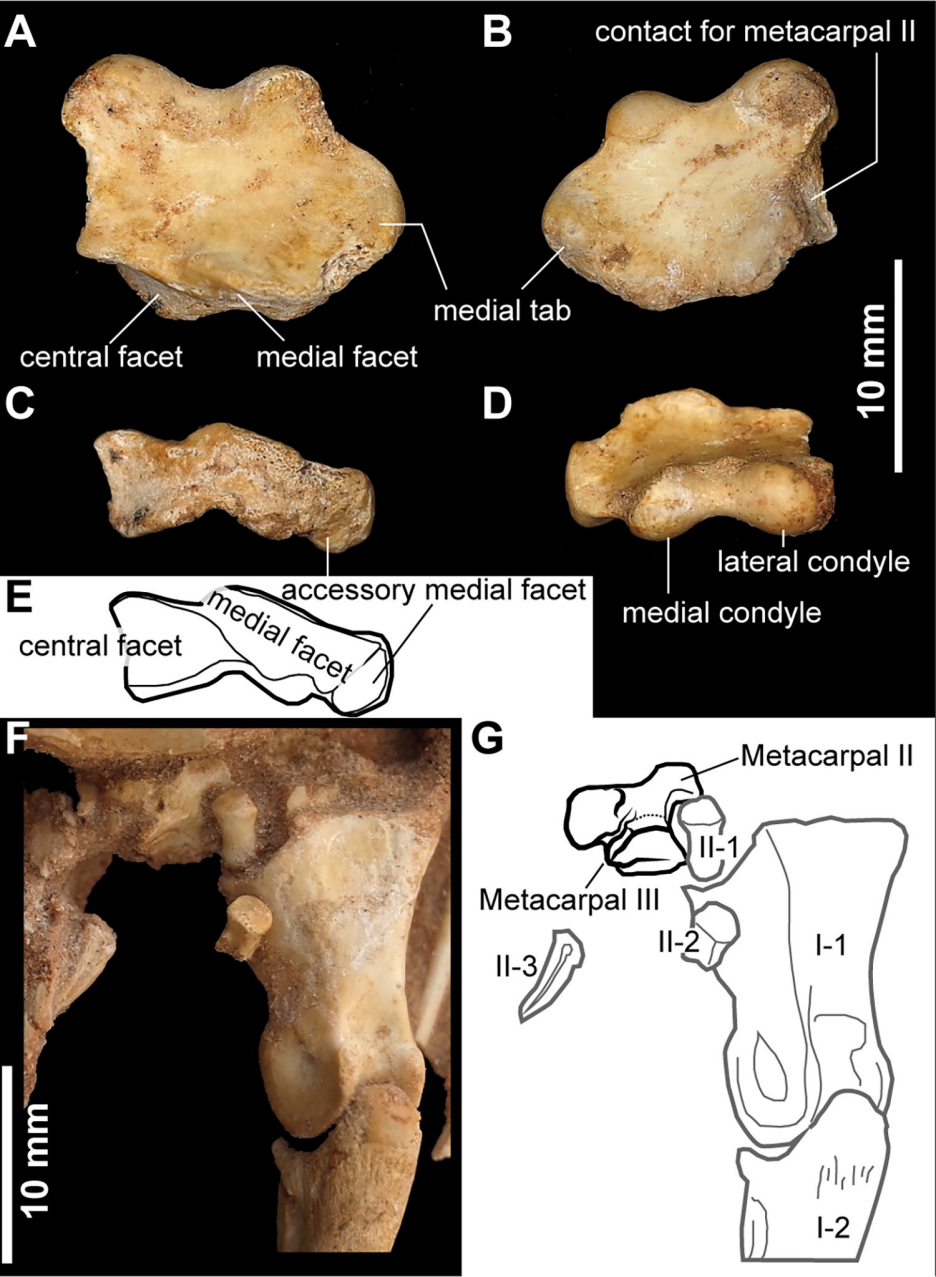
Manual elements of *Jaculinykus yaruui* gen. et sp. nov. (MPC-D 100/209). Left carpometacarpal element (metacarpal I) in dorsal (**A**), ventral (**B**), proximal (**C, E**) and distal (**D**) views. Left manus in oblique view (**F, G**).

As in other alvarezsaurids, the distal carpals are completely fused to metacarpal I, forming the main carpometacarpal element (metacarpal I) [1,15], (Fig 12A). The main carpometacarpal element is proximodistally short, transversely broad, and dorsoventrally flat. In proximal view, there are three articular facets: central, medial, and accessory medial facets, respectively (Fig 12B and 12C). This study follows the terminology of the proximal end of the carpometacarpus in Chiappe et al. [1], and Xu et al. [15]. The accessory medial facet is short relative to the medial and central facets and faces proximomedially. The medial facet is a transversely grooved trochlea, which corresponds to the ancestral semilunate carpal. This groove is shallow and extends slightly onto the dorsal surface of the main carpometacarpal element. Lateral to the medial facet, the central facet is a large fossa bordered by convex dorsal and straight ventral margins as in *Mononykus olecranus*.

The medial tab of metacarpal I is more distally positioned than the early-branching alvarezsauroids, such as *Haplocheirus sollers* and *Bannykus wulatensis*, and is more strongly developed than those of *Mononykus olecranus*, *Shuvuuia deserti*, and *Linhenykus monodactylus* (Fig 12A and 12B). The degree of its development is comparable to early-branching alvarezsauroids *Haplocheirus sollers* and *Bannykus wulatensis*. The lateral surface of metacarpal I is curved laterally and bears a groove for articulation with metacarpal II, as in other alvarezsaurids [1,15]. The distal end of metacarpal I bears two articular condyles without collateral ligamental fossae. The lateral condyle is larger than the medial one.

Metacarpal II is not incorporated into the main carpometacarpal element as in *Linhenykus monodactylus* and *Shuvuuia deserti* (Fig 12F), but unlike the carpometacarpus of *Mononykus olecranus* [33] and *Ondogurvel alifanovi* [31], which is fused to metacarpals II–III. Metacarpal II is much smaller and slenderer than metacarpal I. The distal articular condyles of metacarpal II are well developed as in *Mononykus olecranus* and *Shuvuuia deserti*, but unlike *Linhenykus monodactylus*, in which the distal end of metacarpal II is sharply truncated [15]. The metacarpal III is much smaller than that of the metacarpal II, which is comparable to or even greater than that in *Mononykus olecranus*. The distal end of the metacarpal III is also greatly reduced or truncated as in that of the metacarpal II in *Linhenykus monodactylus*. These features, as well as presence of the digits I–II completely preserved, suggest the loss of the digit III in *Jaculinykus yaruui*.

**Fig 13:**
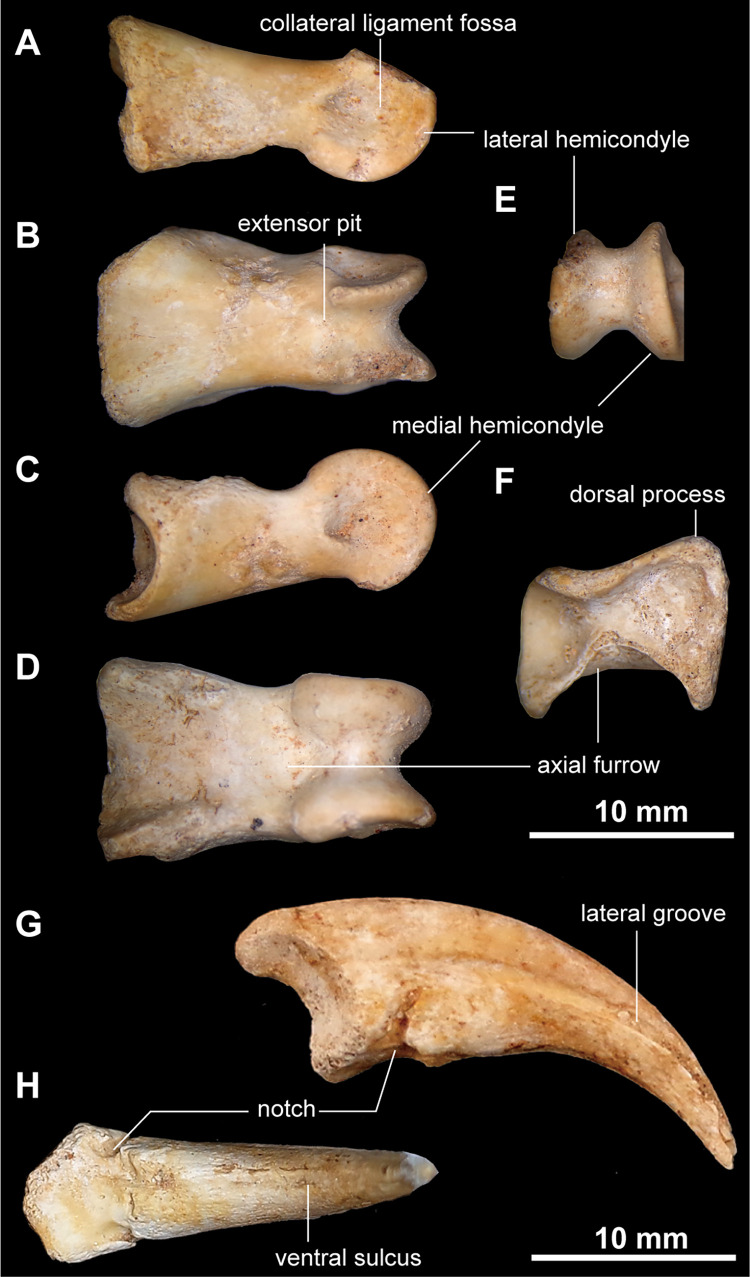
Right phalanx I-1 and left ungual of the digit I of *Jaculinykus yaruui* gen. et sp. nov. (MPC-D 100/209). Right phalanx I-1 in lateral (**A**), dorsal (**B**), medial (**C**), ventral (**D**), distal (**E**), and proximal (**F**) views. Left ungual of the digit I in medial (**G**) and ventral (**H**) views.

Phalanx I-1 is the largest phalanx in the manus and is mediolaterally wider than dorsoventrally (Fig 13A–13F). The proximodorsal process is developed on the lateral corner of the proximal end of phalanx I-1, displaying an asymmetrical shape of the proximal articulation that is seen in alvarezsaurids [1,15], but unlike a symmetrical or weak asymmetrical shape of *Dzharaonyx eski* [17,60], (Fig 13F). The proximodorsal process is less developed than *Mononykus olecranus* and *Linhenykus monodactylus* [1,15]. The shaft of phalanx I-1 is concave with a deep axial furrow ventrally, which is an alvarezsauroid synapomorphy [4,15]. The dorsal surface of phalanx I-1 is transversely narrow dorsally and slightly concave proximodorsally and distally (Fig 13E and 13F). The distal articulation of phalanx I-1 is strongly ginglymoid and bears robust collateral ligament fossae. Dorsally, a narrow intercondylar groove separates lateral and medial condyles of the ginglymus and extends proximally to an extensor pit. Phalanges of digit II are straight and much smaller than phalanx I-1 (Fig 12F). In contrast to phalanx II-1, phalanx II-2 bears a ginglymoid articulation on its distal end.

The ungual of digit I is robust and strongly curved in lateral view (Fig 13G and 13H), unlike the ungual of digit II being gracile and straight (Fig 12F). The lateral and medial sheath grooves are well developed on both ungual phalanges. The grooves on the ungual of digit I curve ventrally at its proximal end and continue to the ventral notch as in other alvarezsaurids [24]. Ventrally, the shallow sulcus runs along the midline of the ungual of digit I (Fig 13H). Whereas non-alvarezsaurid alvarezsaurs possess a prominent flexor tubercle on their unguals [4,8,14,61], *Jaculinykus yaruui* lacks it as in other alvarezsaurids [1,15,24]. This is ontogenetically invariable [18,19].

#### Pelvic girdle

The ilium is dorsoventrally low, and its anteroposterior length is about four times as long as the iliac height above the center of the acetabulum (Fig 7A and 7B). As in other alvarezsaurids, the postacetabular process is anteroposteriorly longer and dorsoventrally lower than the preacetabular process. The iliac blades abut to the neural spines of the sacrum along all of the dorsal edges except the posterior part of the postacetabular process, where they diverge laterally (Fig 7D). The supracetabular crest overhangs laterally, especially along the anterior half part of the acetabulum, and terminates anterior to the ischial peduncle. Ventrally, the preacetabular process curves medially and lacks the cuppedicus fossa (Fig 7E). The brevis fossa on the postacetabular process is large and tapers towards the posterior end as in other alvarezsaurids [1,25]. Although the pubic and ischial peduncles are poorly preserved on both sides, they are deflected medially relative to the iliac blade as in *Xixianykus zhangi* [25]. The pubic peduncle is transversely compressed and more prominent than the ischial peduncle (Fig 7C). Lateral to the ischial peduncle, the antitrochanter is strongly expanded laterally and forms the posterior and anterior margins of the acetabulum and brevis fossa (Fig 7C and 7D).

Similar to other alvarezsaurids, the pubis and ischium extend posteroventrally and abut each other along their entire lengths [1,25], (Fig 7A and 7B). The proximal end of the pubis is compressed transversely and is inclined lateroventrally. The preacetabular tubercle is elongated relative to those of *Xixianykus zhangi* [25] and *Trierarchuncus prairiensis* [18,62], (Fig 7C). The obturator notch is bordered ventrally by a weak ridge along the posterior margin of the pubic shaft. The gracile pubic shafts are mostly straight in lateral view and abuts its counterpart throughout the entire length. A pubic apron is absent as in other alvarezsaurids [1], but unlike the early-branching alvarezsauroid, *Haplocheirus sollers*, where it is developed on the three-quarters of the length of the pubis [50].

The ischium is more slender than the pubis and rod-like (Fig 7A–7C). The proximal end of the ischium extends posterodorsally. The ischial shaft has a sub-oval in cross-section and is less compressed transversely than the pubic shaft as in *Ondogurvel alifanovi* [31], but unlike those of *Parvicursor remotus*, *Xixianykus zhangi* and *Shuvuuia deserti* (MPC-D 100/99).

#### Hind limb

**Fig 14:**
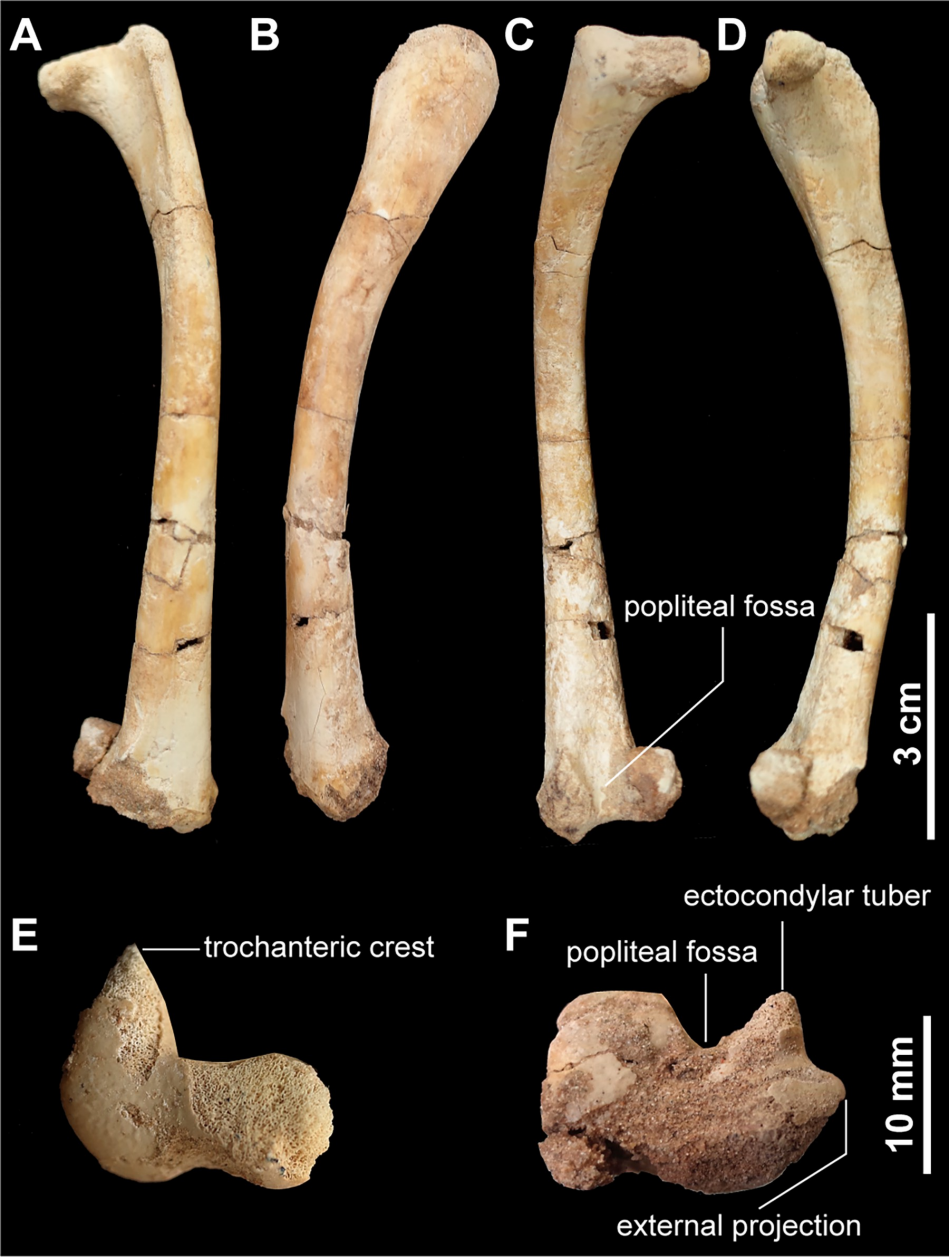
Femora of *Jaculinykus yaruui* gen. et sp. nov. (MPC-D 100/209). Left femur in anterior (**A**), lateral (**B**), posterior (**C**), medial (**D**), and proximal (**E**) views. Right femur in distal views (**F**). Scale bar are 3 cm for (A) to (D) and 1 cm for (E) to (F).

The slender femur is nearly as long as the metatarsus and is bowed anteroposteriorly, as in other alvarezsaurids [1]. In addition, the femoral shaft is more strongly bowed mediolaterally than in other alvarezsaurs (Fig 14A and 14C). Proximally, the trochanteric crest (fused great and lesser trochanters) is anteriorly projecting from the femoral head, forming an L-shape outline in proximal view (Fig 14E). The stout and straight trochanteric crest is similar to those of *Mononykus olecranus* and *Parvicursor remotus* but differs from other alvarezsaurids which are medially curved. The femoral head is separated from the femoral shaft by a slight neck on its posterior margin. The femoral head is stout relative to those of *Mononykus olecranus*, *Xixianykus zhangi*, and *Linhenykus monodactylus*. The fourth trochanter is absent on the posterior surface of the femur as in some alvarezsaurids [1,15,28], (Fig 14C). Distally, the ectocondylar tuber on the lateral condyle differs from other alvarezsaurids in being a sharp ridge and does not extend posteriorly to the level of the medial condyle. In distal view, the ectocondylar tuber is also separated from the medial condyle by the popliteal fossa (Fig 14F). This condition is also seen in other alvarezsaurids and *Patagonykus puertai* [1], but not in *Mononykus olecranus* or *Xixianykus zhangi* which are partially or fully closed distally [15,25,33]. The lateral condyle also forms a strong external projection on its lateral surface comparable to the structure in *Mononykus olecranus* and *Parvicursor remotus*.

**Fig 15:**
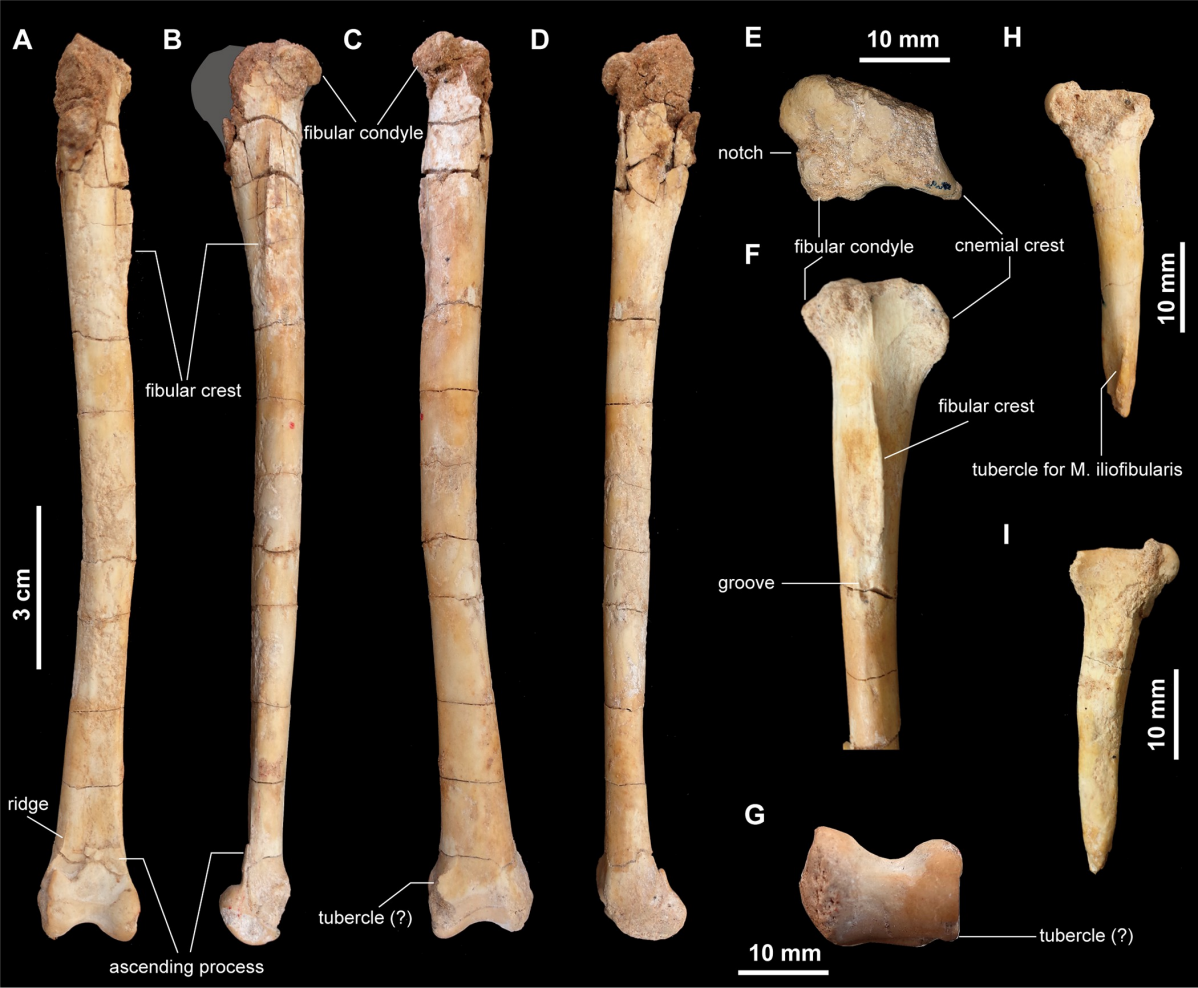
Tibiotarsi and right fibula of *Jaculinykus yaruui* gen. et sp. nov. (MPC-D 100/209). Left tibiotarsus in anterior (**A**), lateral (**B)**, posterior (**C**), and medial (**D**) views. Proximal part of the right tibia in proximal (**E**) and dorsal (**F**) views. Distal end of the left tibiotarsus in distal view (**G**). Right fibula in lateral (**H**) and medial (**I**) views. Gray area indicates missing part.

As in *Patagonykus puertai* [1] and other alvarezsaurids, the tibia is fused to the calcaneum and astragalus, forming a tibiotarsus (Fig 15A). It is the longest element of the hind limb and slightly bowed laterally. The rounded cnemial crest is well developed along the anterolateral margin of the proximal part of the tibia (Fig 15E and 15F). In proximal view, the cnemial crest is separated from the fibular condyle by a broad lateral indentation as in *Xixianykus zhangi* [25] and *Nemegtonykus citus* [34], (Fig 15B). The posterior margin of the tibia is divided into fibular and medial condyles by a shallow notch. The rounded medial condyle of *Jaculinykus yaruui* differs from other alvarezsaurids in being robust relative to the fibular condyle. Posterior to the fibular crest along the proximal fourth of the tibiotarsus, there is a longitudinal groove extending distally. Distal to this groove, the shaft of the tibiotarsus is anteroposteriorly compressed and sub-oval in cross-section. There is a small tubercle on the posterolateral surface of the distal end of the tibiotarsus (Fig 15C and 15G). Although poorly preserved, the small tubercle may be homologous to that in *Nemegtonykus citus* [34]. The base of the ascending process of the astragalus is sharply indented medially, being unique among alvarezsaurids.

The fibula (Fig 15H and 15I) is strongly reduced, compared with the tibia, not reaching the calcaneum as in other alvarezsaurids [1]. The proximal end of the fibula is compressed mediolaterally and projects posteriorly. The shaft is straight, unlike that in *Xixianykus zhangi*, which is curved anteriorly [25]. The shaft also tapers distally and has a prominent tubercle for the *M*. *iliofibularis* on the lateral surface of its distal part.

**Fig 16:**
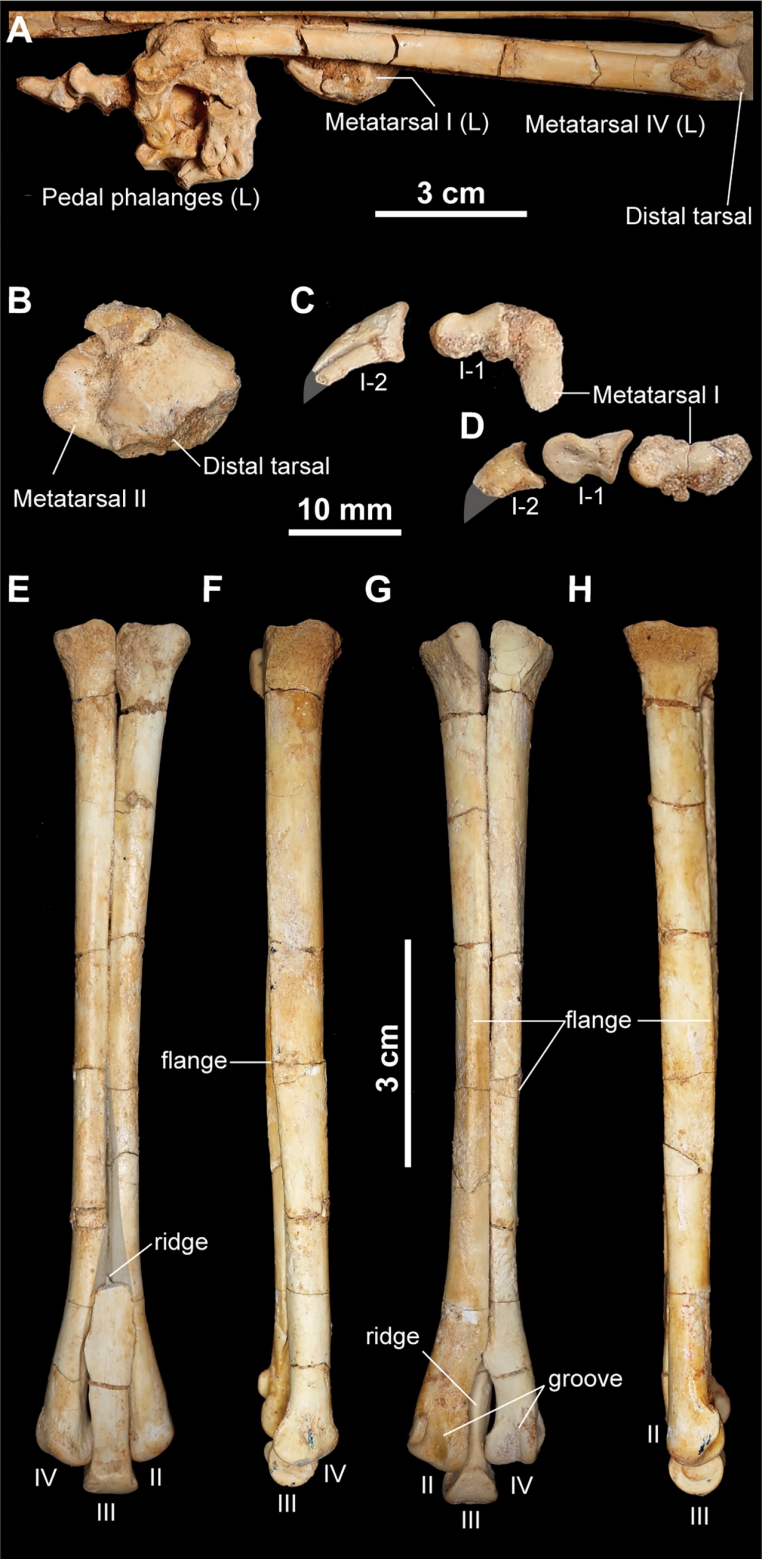
Distal tarsals and metatarsus of *Jaculinykus yaruui* gen. et sp. nov. (MPC-D 100/209). Left foot in lateral view (**A**). Left distal tarsals and metatarsal II in proximal view (**B**). Left metatarsal I and digit I in lateral view (**C**). Right metatarsal I and digit I in medial view (**D**). Right metatarsus in anterior (**E**), lateral (**F**), posterior (**G**), and medial (**H**) views. Gray areas indicate missing parts.

The left distal tarsals are preserved in articulation with metatarsals II and IV (Fig 16A and 16B). The flat and disk-shaped distal tarsals are not co-ossified unlike those in *Xixianykus zhangi* [25], *Albinykus baatar* [5], and *Nemegtonykus citus* [34]. Metatarsus shows an extreme arctometatarsalian condition as in other alvarezsaurids [1,5]. The small metatarsal I tapers proximally and possesses a weakly developed ginglymus (Fig 16C and 16D). Metatarsals II and IV are sub-equal in length and have a sharply longitudinal flange for the *M*. *gastrocnemius* along posterior margins (Fig 16E–16H). The shaft of both metatarsals II and IV is transversely compressed and contact each other by an entire area except for the distal end. Similar to other alvarezsaurids, the distal articulations of metatarsals II and IV lack ginglymus but possess deeply grooved articular facets on their posterior surfaces (Fig 16G). Metatarsal III tapers proximally and is wedged in cross-section with a sharp posterior ridge as in other alvarezsaurids [1,24], (Fig 16E). Distally, metatarsal III has a symmetrical articulation lacking an intercondylar groove.

**Fig 17:**
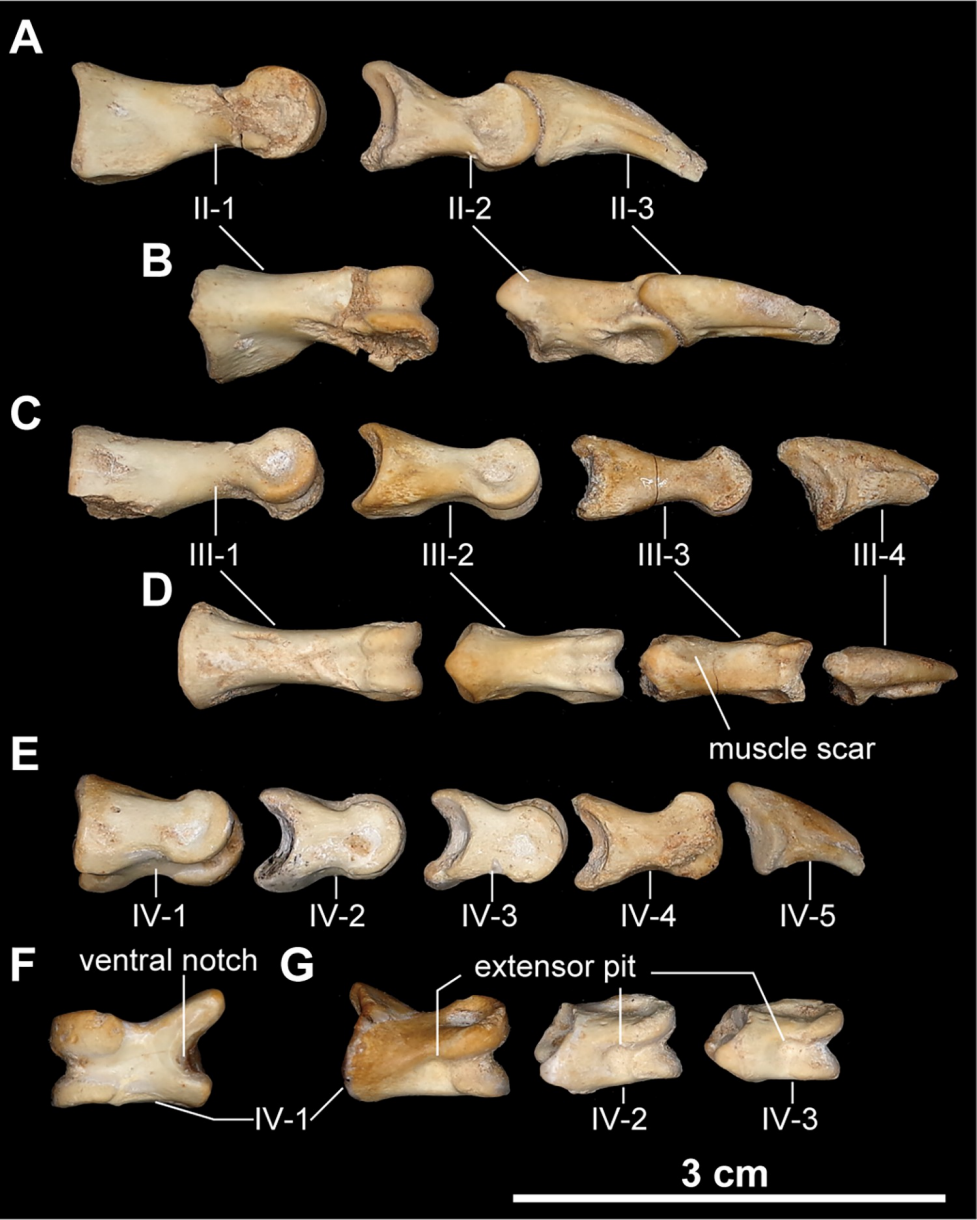
Right pedal digits of *Jaculinykus yaruui* gen. et sp. nov. (MPC-D 100/209). Digit II in lateral (**A**) and dorsal (**B**) view. Digit III in lateral (**C**) and dorsal (**D**) views. Digit IV in lateral (**E**), dorsal (**F**), and ventral (**G**) views.

All the pedal phalanges are preserved in articulation, exhibiting the phalangeal formula 2-3-4-5-0 (Figs 16A, 16C, 16D and 17). The digit III is the longest, and the digit I is the shortest. Each non-ungual pedal phalanx has a well-developed ginglymoid articulation. As in other alvarezsaurids, there is an unusual muscle scar on the proximodorsal surface of phalanx III-3 [24], (Fig 17D). The extensor ridges on the phalanges of the digit IV are well-developed with a deep extensor pit relative to other digits, which is common among alvarezsaurids [5]. The proximal end of phalanx IV-1 has a ventral notch on its ventral surface, dividing proximomedial and proximolateral processes as in alvarezsaurids [15,24], (Fig 17F). Each ungual of all digits lacks a distinct flexor tubercle and possesses an L-shaped lateral groove, as in other alvarezsaurids.

#### Phylogenetic analysis

**Fig 18:**
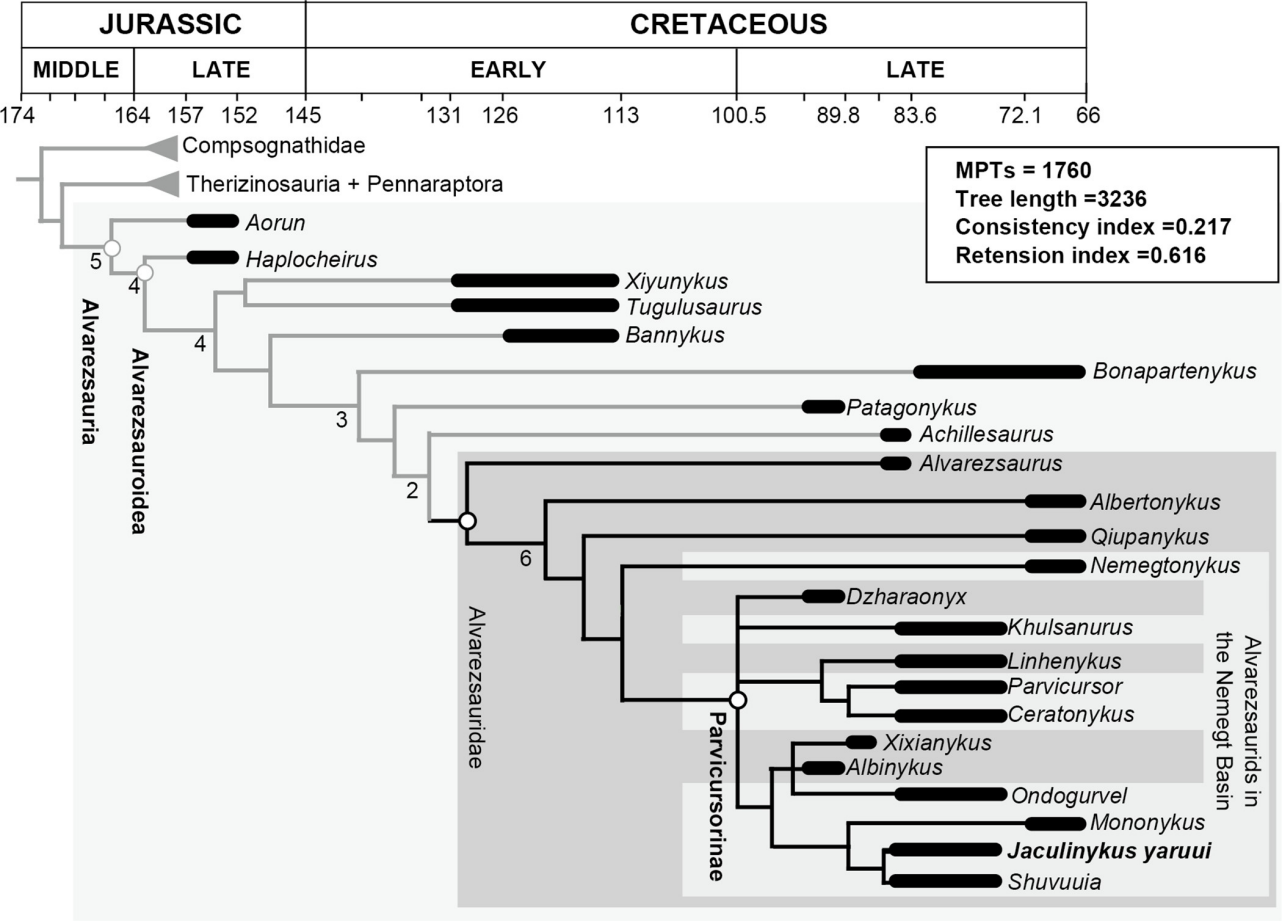
Consensus tree of the 1730 most parsimonious trees in this analysis. A grey shadow represents alvarezsaurids. The numbers at nodes represent Bremer decay values. Bremer values below 1 are not shown.

Our phylogenetic analysis produced 1730 most parsimonious trees with a tree length of 3236 (Fig 18). The strict consensus of the most parsimonious trees recovers *Jaculinykus yaruui* as a member of Alvarezsauridae, supported by 557 (1), fibular facet on astragalus is reduced and facing laterally or absent. It also is placed in Parvicursorinae, a monophyletic clade supported by 286 (1) size of neural canal of dorsal vertebrae is large and subequal to posterior articular facet, 287 (2) posterior dorsal vertebra is much longer than high, 335 (2) prezygapophyses of posterior caudal vertebrae are strongly reduced, 345 (1) proximal end of chevron of anterior caudals elongate anteroposteriorly, flattened and plate-like, 370 (1) presence of notch on posterior margin of scapular blade immediately dorsal to glenoid lip, and 468 (1) pubic peduncle is anteroposteriorly elongated and narrow.

*Alvarezsaurus*, *Albertonykus*, *Qiupanykus*, and *Nemegtonykus* occur as successive sister taxa to the clade of Parvicursorinae. *Parvicursor* and *Ceratonykus*, two taxa from the Baruungoyot Formation, form a monophyly that is sister to *Linhenykus*. The clade including *Jaculinykus* occurs in a polytomy with the clade of *Linhenykus* + (*Parvicursor* + *Ceratonykus*), *Khulusanurus*, and *Dzharaonykus*. The clade *Mononykus* + (*Jaculinykus* + *Shuvuuia*) is sister to an unresolved clade including *Albinykus*. The monophyly of *Jaculinykus* and *Shuvuuia* is supported by two synapomorphies: 265 (0) anterior cervical centra length is less than twice transverse centrum width; 266 (2) anterior articular facet of anterior cervicals is kidney-shaped with neural canal emarginating dorsal aspect. *Jaculinykus* has six autapomorphies: 263 (2) ventral surfaces of anterior cervicals with ventral depression; 415 (1) proximomedial tab projects far proximally; 527 (1) flange-like or medially extensive femoral medial epicondyle; 528 (0) popliteal fossa opens distally; 595 (1) the ischial shaft is slender compared to the pubic shaft.

## Discussion

### Taxonomic remarks and diversity of alvarezsaurids in the Nemegt Basin

*Jaculinykus* represents one of the most complete alvarezsaur skeletons reported so far. Although no histological analysis is performed, the complete closure of the neurocentral suture in the cervical, dorsal, and caudal vertebrae, fusion of sacral vertebrae, and co-ossification between proximal tarsals and tibia suggest that the individual was at least a late ontogenetic stage or close to maturity [6,63–65]. However, although the distal carpals are fused with the base of the metacarpal I, the metacarpal II is not incorporated into the fused metacarpal element unlike those of *Mononykus* [2,33], *Linhenykus* [7,15], and *Ondogurvel* [31]. Given the ontogenetic stage of *Jaculinykus*, these differences in the fusion of the metacarpals among late-branching alvarezsaurids likely demonstrate interspecific variation rather than ontogenetic variation. Thus, the feature in the fusion of metacarpals of *Jaculinykus* may represent its autapomorphy.

In addition to the autapomorphies recovered from the phylogenetic analyses, *Jaculinykus* is unique among alvarezsaurids in the extraordinary development of the medial tab of metacarpal I that is comparable to early-branching alvarezsauroids *Haplocheirus* and *Bannykus* (Fig 12A). It further differs among alvarezsaurids in the weakly developed proximodorsal process of manual phalanx I-1 (Fig 13F). In addition, *Jaculinykus* is unique among alvarezsaurids in: the subtriangular deltopectoral crest that is separated from the humeral head by a notch, unlike the pillar-like deltopectoral crest of *Mononykus* [3,33] and the unseparated deltopectoral crest of the other alvarezsaurids (Fig 10B); the large medial condyle of the tibia compared to the fibular condyle, whereas other alvarezsaurids have a smaller medial condyle than the fibular condyle (Fig 15B); and the deeply indented base of ascending process. These unique characters suggest *Jaculinykus* as a new genus, the ninth alvarezsaurid from the Nemegt Basin (Fig 1B), emphasizing the high diversity of the group in this area.

*Jaculinykus* is an addition to a remarkable diversity of late-branching alvarezsaurids in the Nemegt Basin bearing the Djadokhta, Baruungoyot, and Nemegt formations [2,3,22,27–32,34]. The Djadokhta and Baruungoyot formations, where the former represents mostly aeolian deposits [35,37,66] and the latter both aeolian and alluvial deposits [35,36,67], have been interpreted as drier environments than the Nemegt Formation dominated by fluvial and alluvial plain deposits [35,36]. The alvarezsaurid materials are abundant in the former two formations but scarce in the Nemegt Formation, previously implying that they preferred dry habitats rather than wet habitats. The phylogenetic interrelationships of late-branching alvarezsaurids, proposed in the present study, demonstrate two clusters that lived in arid to semi-arid environments, such as the clade of *Jaculinykus* and *Shuvuuia* as well as the monophyly including *Linhenykus* from the Wulansuhai Formation in Inner Mongolia characterized by aeolian deposits [68], (Fig 18). However, their inferred habitats are not clustered significantly by whether they lived in mesic or arid environments. Indeed, multiple alvarezsaurid specimens including *Nemegtonykus* from the Nemegt Formation also show not a definitively lower alvarezsaurid diversity and abundance in the Nemegt Formation than in the other two formations [34]. These suggest that alvarezsaurids in the Nemegt Basin were successfully adapted for both mesic and arid environments, rather than that they were specialists adapted for arid environments. Furthermore, overlapping vertebrate faunal compositions in the Djadokhta, Baruungoyot, and Nemegt formations, which consist of non-avian dinosaurs [35,69,70], birds [35], and mammals [35,71], suggest that the stratigraphic relationships between three formations in the Nemegt Basin perhaps represent a lateral transition order during a short and coeval time rather than a chronostratigraphic order [35,71]. Therefore, late-branching alvarezsaurids represent the characteristic dinosaur groups in the Nemegt Basin bearing both arid and wet environments and likely diversified during a relatively short time span of the Late Cretaceous in this area.

### Evolution and specialization of alvarezsaur hands

**Fig 19:**
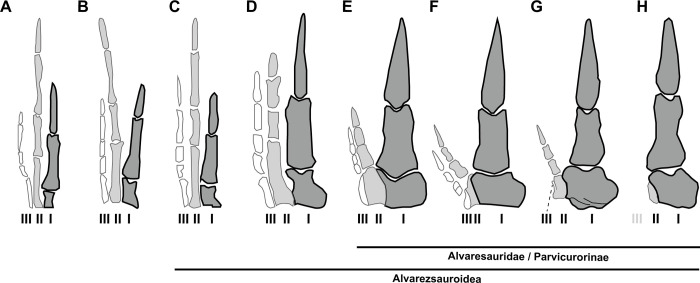
Comparisons of the metacarpals and manual digits of alvarezsaurs in dorsal view. **(A)**, *Aorun zhaoi*; **(B),** *Shishugounykus inexpectus*; **(C),** *Haplocheirus sollers*; **(D),** *Bannykus wulatensis*; **(E)**, *Dzharaonyx eski*; **(F)**, *Shuvuuia deserti*; **(G)**, *Jaculinykus yaruui*; **(H)**, and *Linhenykus monodactylus* (not to scale). (A)–(F) and (H) were modified from [8,60]. Manual digits I–III are shown in grey, light grey, and white respectively.

The manus of late-branching alvarezsaurids (parvicursorines) is the most strikingly specialized part of their skeleton. Evolutionary sequence of alvarezsaurs documents that digital reduction and enlargement of the thumb have occurred in a stepwise manner [11], which suggests a functional shift from grasping to digging (Fig 19). The Jurassic taxa possess the manus retaining grasping function, as in typical theropods [4,8]. Subsequently, during the Early Cretaceous, the modified manus with a hypertrophied digit I and shortened lateral digits has appeared in non-alvarezsaurid alvarezsauroid, *Bannykus*. Finally, the late-branching alvarezsaurids have acquired a highly specialized manus with only one functional digit (digit I) and extremely reduced lateral digits at least since the early Late Cretaceous (Turonian), [17,60]. Notably, *Jaculinykus* has only two fingers, although the manual elements of both sides are completely preserved. The metacarpal III of *Jaculinykus* also presents only its truncated articular facet, which is nearly identical to the metacarpal II of *Lihenykus* [7,15], (Fig 19G and 19H). Due to the above features, the manus of *Jaculinykus* possibly possesses only a single lateral manual digit, suggesting an intermediate condition between *Shuvuuia* with two lateral manual digits and *Linhenykus* without them. This illustrates another example of an extreme digital reduction within alvarezsaurids, as well as confirms their variations in vestigial lateral digits proposed previously [7].

The metacarpal I of *Jaculinykus*, by contrast, exhibits a greatly developed medial tab, which is comparable to those in early-branching alvarezsaurs, rather than late-branching alvarezsaurids. The dorsal process of the manual phalanx I-1 is also less developed in *Jaculinykus* than in *Mononykus*, *Shuvuuia*, and *Linhenykus*, suggesting a primitive condition among late-branching alvarezsaurids [60]. Thus, the manus of *Jaculinykus* was less derived in the above features but more derived with respect to the loss of phalanges on digit III. Indeed, this mixed condition of primitive and derived features are also reported in the manus of *Linhenykus* [7] and early-braching alvarezsaurs such as *Aorun* and *Shishugounykus* [8]. This highlights a more complex evolutionary history in alvarezsaur hand involving mosaic evolution on small scales than previously thought.

### Behavioral implications of tuck-in sleeping posture in *Jaculinykus*

**Fig 20:**
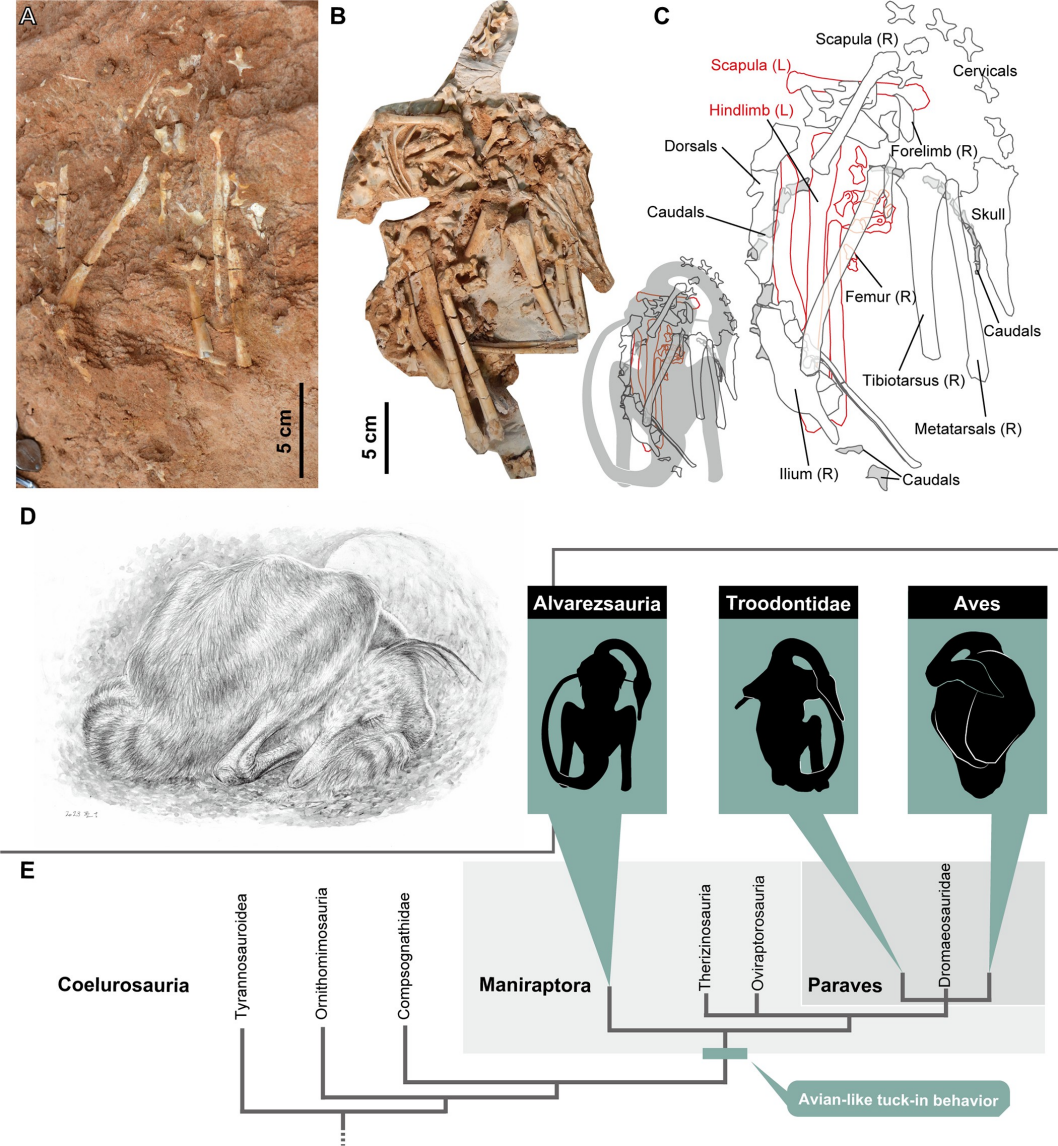
Evolution of avian-like sleeping posture in theropod dinosaurs. Skeletal disposition of *Jaculinykus yaruui* gen. et sp. nov. (MPC-D 100/209) in dorsal (photograph courtesy of Tomonori Tanaka) (**A**) and ventral (reversed) (**B**) views. **(C)**, Interpretive line drawing of skeletal disposition. **(D)**, Life restoration of sleeping posture of *Jaculinykus yaruui* (Artwork courtesy of Seiji Yamamoto). **(E),** Simplified coelurosaurian phylogeny represents presence for evidence of avian ‘tuck-in’ posture.

The posture of *Jaculinykus* shows the following features: the hind limbs folded on either side of the body; the left forelimb folded next to the body with the elbow; the neck curved posteriorly on the right side of the body; the tail positioned on the left side and curled around the flexed hind limbs to the right (Figs 2A, 2B and 20A–20C). Despite displacements of both forelimb elements of *Jaculinykus*, these bones are tucked underneath the body as in the hind limb. This posture obviously differs from the opisthotonic posture commonly seen in theropod dinosaurs [72], in which the body lies on one side with both neck and tail arched dorsally. The specimen was dorsoventrally crushed during or after burial, and this seems to have resulted in twisted displacement of the posterior half of the body and lateral displacement of forelimb elements and caudal vertebrae. However, the articulation of most skeletal elements, and positions of all the bones without significant deviations from their original positions, suggest relatively little effect of decay or transport prior to burial. Given this condition, we infer that the position of the skeleton reflects a sleeping posture prior to death or burial (Fig 20A–20C). This posture is identical to the ‘tuck-in’ sleeping posture seen in troodontids [38–41,73] and potentially oviraptorids [74–76].

*Jaculinykus* newly demonstrates the evolution of avian behavior or perhaps physiological states in non-avian dinosaurs (Fig 20E). Although the crouched posture is also preserved in *Albinykus* and perhaps *Haplocheirus* among alvarezsaurs which represents folded hind limbs under the pelvis [4,5], the former is only preserved in the pelvis and hind limbs, and the latter shows its neck and tail unfolded. The position of *Shuvuuia* (MPC-D 100/120) can be more similar to that of *Jaculinykus* than the two others, where its neck curves posteriorly on the left side and its hind limbs are partially folded, but the specimen is incomplete [22]. Thus, the posture of *Jaculinykus* displays the first clear record of the avian ‘tuck-in’ behavior among alvarezsaurs. The sleeping behavior specific to modern birds, tucking their heads between one of their forelimbs and their torso [77], is usually considered to be associated with heat conservation [78–80]. Whereas the crouched postures, similar to birds and some mammals, are present in theropods [4,5,38,39,74,75,81–83] but also in basal sauropodomorphs [84] and ornithischians [85], the definitive evidence of the avian tuck-in sleeping posture had been so far restricted to few troodontids, *Mei* and *Sinornithoides* [38,39]. The avian-like sleeping posture in an alvarezsaur dinosaur, *Jaculinykus*, confirms that this avian-like behavior was already present in maniraptorans prior to paravians (Fig 20D). Alternatively, because small vertebrates with high surface-to-volume ratios tend to show specific behaviors for heat conservation, the presence of this avian-like behavior in alvarezsaurs and paravians is likely associated with the miniaturization of body size that underwent only in both lineages independently [5,6,49]. From the phylogenetic position of the fossil, this discovery further supports the hypothesis that alvarezsaur feathers may be more complex structures with a rachis seen in ornithomimosaurs and other maniraptorans for thermoregulation, display, and reproduction [86,87] than simple filament structures previously thought [88]. In either case, these behavioral and physiological implications suggest that the evolutionary processes leading to avian features had proceeded in non-avian dinosaurs, especially maniraptorans, prior to the origin of powered flight.

## Supporting information

S1 Fig Additional images of the teeth of *Jaculinykus yaruui* gen. et sp. nov. (MPC-D 100/209). Teeth associated with matrix (**A, B**) and isolated teeth (**C, D**). Scale bars are 5 mm for (A) and (B), and 1 mm for (C) and (D).
(TIF)

S2 Fig Additional images of dorsal vertebral series of *Jaculinykus yaruui* gen. et sp. nov. (MPC-D 100/209). Dorsal vertebrae in dorsal (**A**) and lateral (**B**) views. Eighth dorsal vertebra is reversed. The numbers indicate the position of dorsal vertebrae. Abbreviations: S, sacral vertebrae. Scale bar is 3 cm.
(TIF)

S3 Fig Additional images of caudal vertebral series of *Jaculinykus yaruui* gen. et sp. nov. (MPC-D 100/209). Middle to posterior caudal vertebrae in lateral view. Scale bar is 5 cm.
(TIF)

S4 Fig Strict consensus of 1,730 most parsimonious trees of 118 taxa with 596 characters (tree length = 3266; consistency index = 0.217; retention index = 0.616). Numbers at each node indicate Bremer support values.
(TIF)

S1 Table Measurements of *Jaculinykus yaruui* gen. et sp. nov. (MPC-D 100/209). Table 1: Cranial elements. Table 2: Vertebral series. Table 3: Pectoral and forelimb elements. Table 4: Pelvic and hind limb elements.
(XLSX)

S1 Appendix Character description and data matrix of Alvarezsauroidea and outgroups used in this study (modified from Averianov and Sues [17]). (DOCX)
