# Supplementary figures and images for: A new alvarezsaurid dinosaur (Theropoda, Alvarezsauria) from the Upper Cretaceous Baruungoyot Formation of Mongolia provides insights for bird-like sleeping behavior in non-avian dinosaurs
Source: PLoS One. 2023 Nov 15;18(11):e0293801. doi: 10.1371/journal.pone.0293801 (PMC10651048; doi:10.1371/journal.pone.0293801)

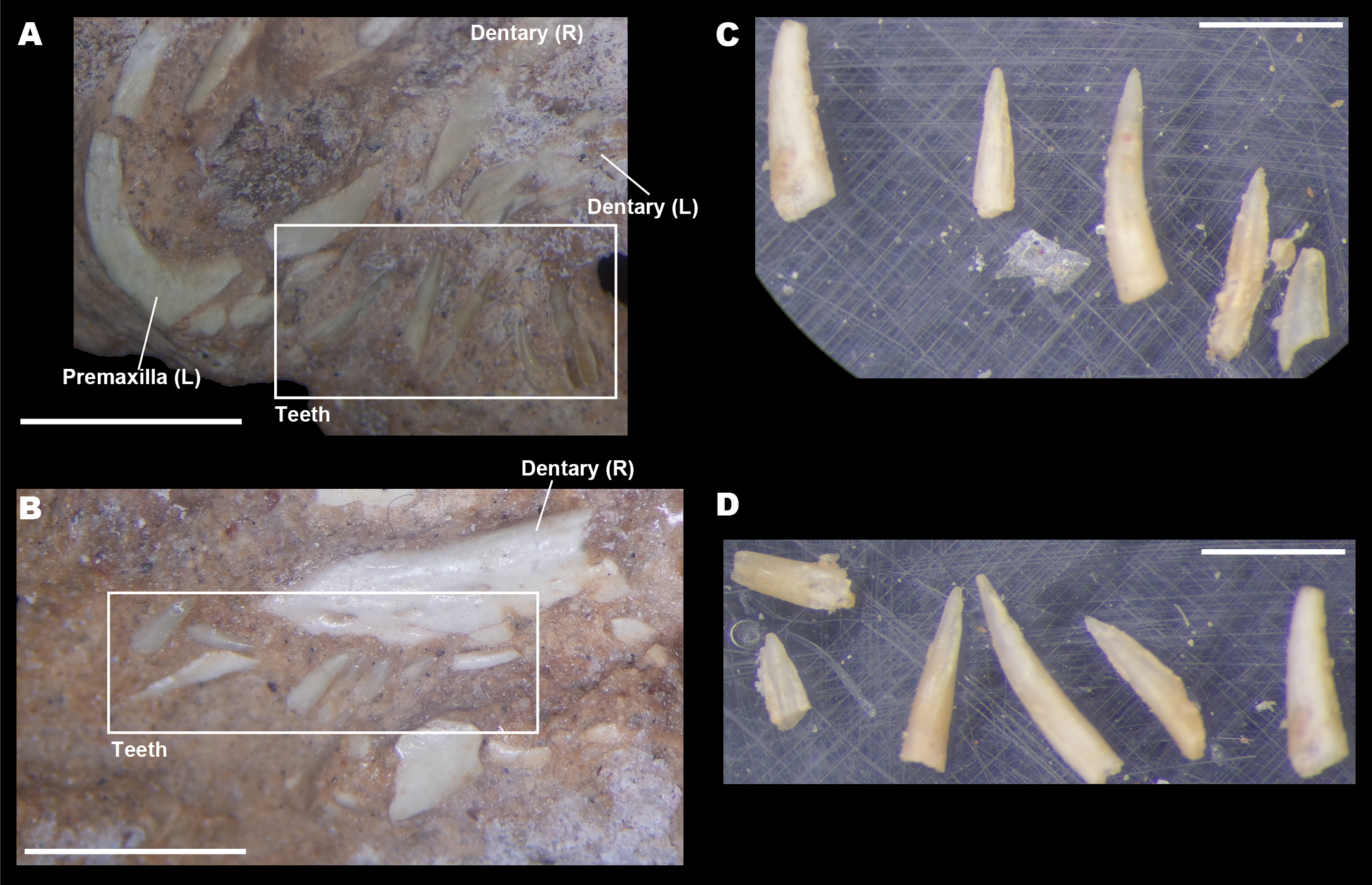

Supplement: S1 Fig — Teeth associated with matrix (A, B) and isolated teeth (C, D). Scale bars are 5 mm for (A) and (B), and 1 mm for (C) and (D). (TIF) [file pone.0293801.s001.tif]

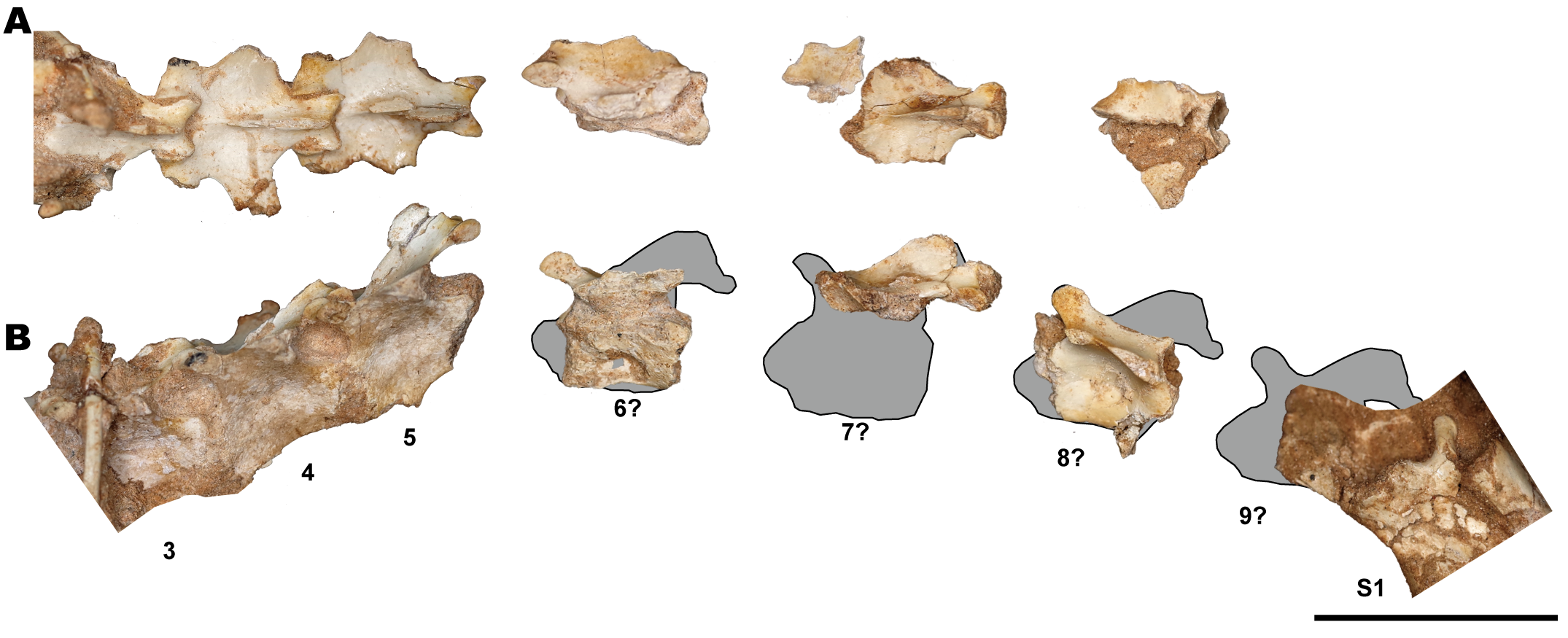

Supplement: S2 Fig — Dorsal vertebrae in dorsal (A) and lateral (B) views. Eighth dorsal vertebra is reversed. The numbers indicate the position of dorsal vertebrae. Abbreviations: S, sacral vertebrae. Scale bar is 3 cm. (TIF) [file pone.0293801.s002.tif]

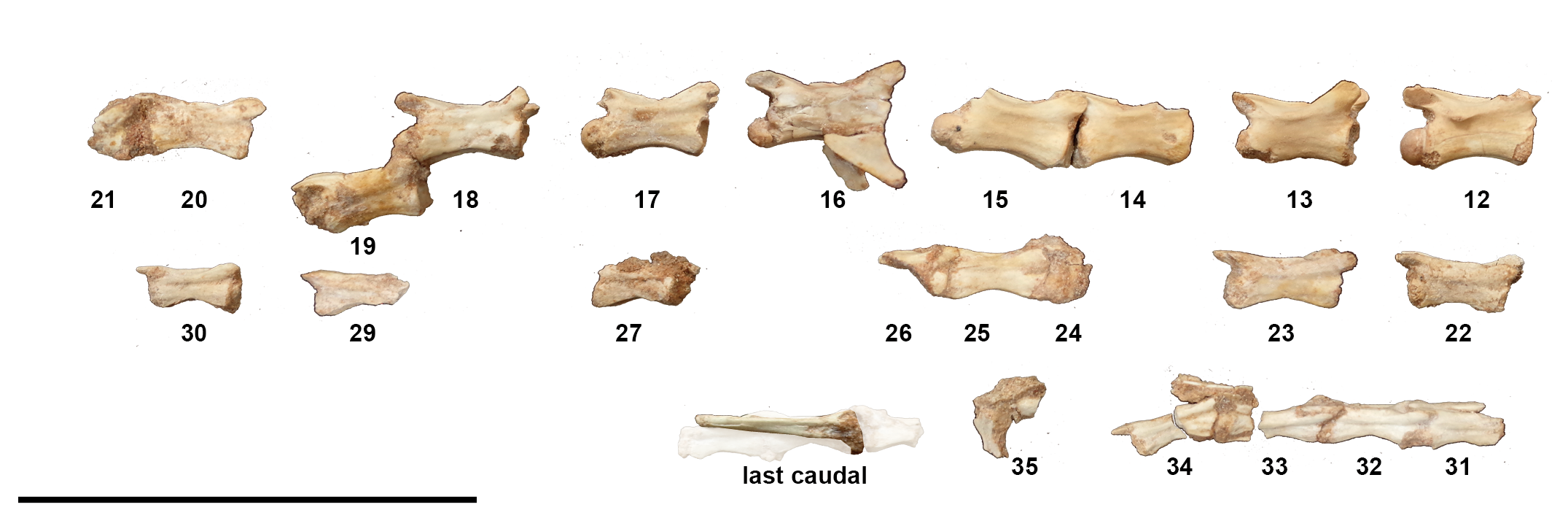

Supplement: S3 Fig — Middle to posterior caudal vertebrae in lateral view. Scale bar is 5 cm. (TIF) [file pone.0293801.s003.tif]

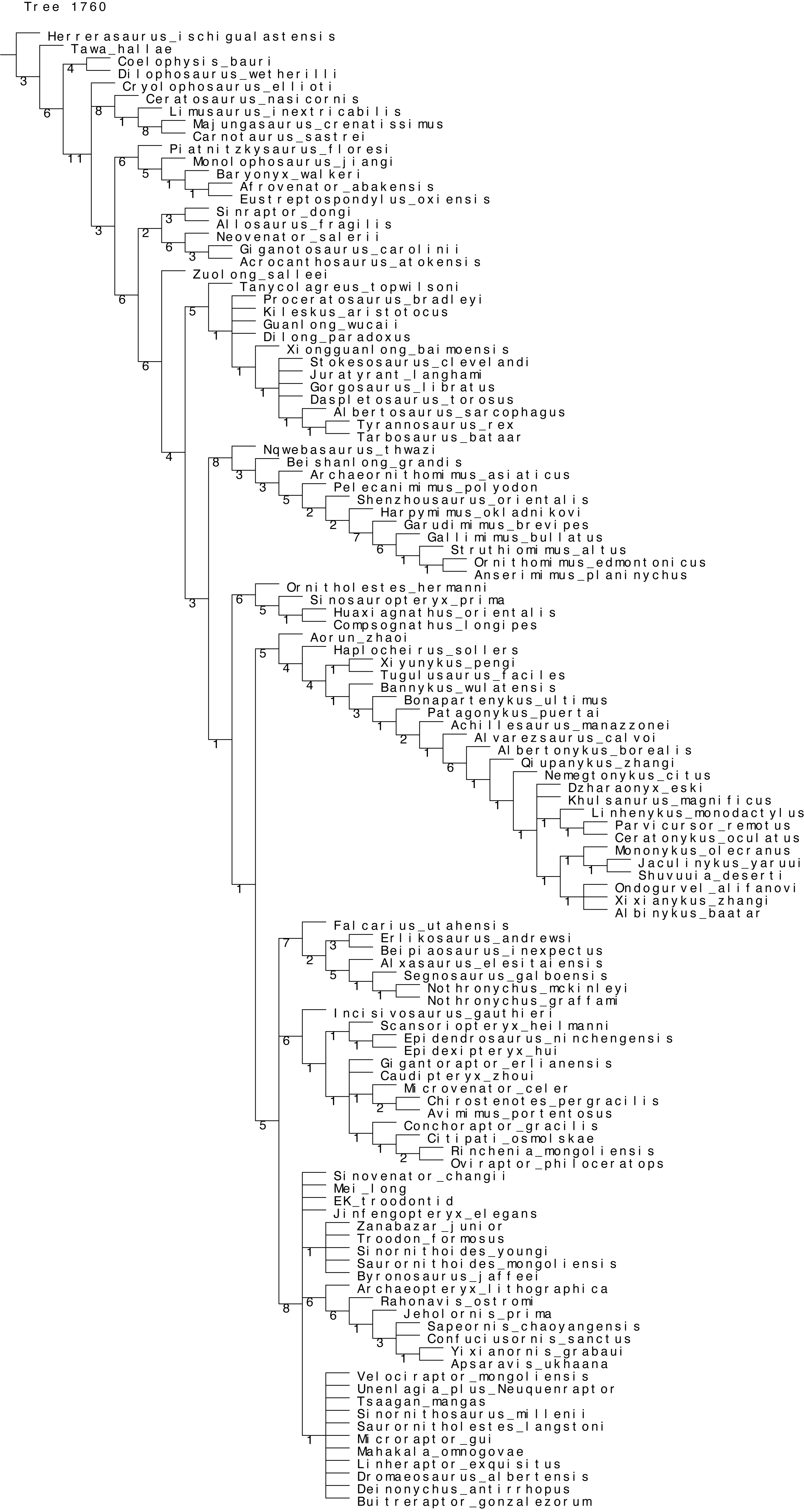

Supplement: S4 Fig — Numbers at each node indicate Bremer support values. (TIF) [file pone.0293801.s004.tif]
