# Supplementary material for: A new alvarezsaurid dinosaur (Theropoda, Alvarezsauria) from the Upper Cretaceous Baruungoyot Formation of Mongolia provides insights for bird-like sleeping behavior in non-avian dinosaurs
Source: PLoS One. 2023 Nov 15;18(11):e0293801. doi: 10.1371/journal.pone.0293801 (PMC10651048; doi:10.1371/journal.pone.0293801)
Supplement: S1 Appendix — (DOCX) [file pone.0293801.s006.docx]

**S1 Appendix**

Character and Character state descriptions

56 characters (Ch. 47, 74, 83, 99, 118, 124, 131, 151, 180, 184, 222, 228, 229, 235, 238, 239, 265, 266, 274, 275, 282, 283, 287, 311, 312, 321, 329, 332, 334, 335, 348, 351, 358, 380, 381, 384, 386, 389, 395, 429, 430, 435, 437, 442, 444, 446, 467, 469, 480, 506,507, 520, 522, 559, 580, and 587) were treated as ordered (additive).

Two new characters are added.


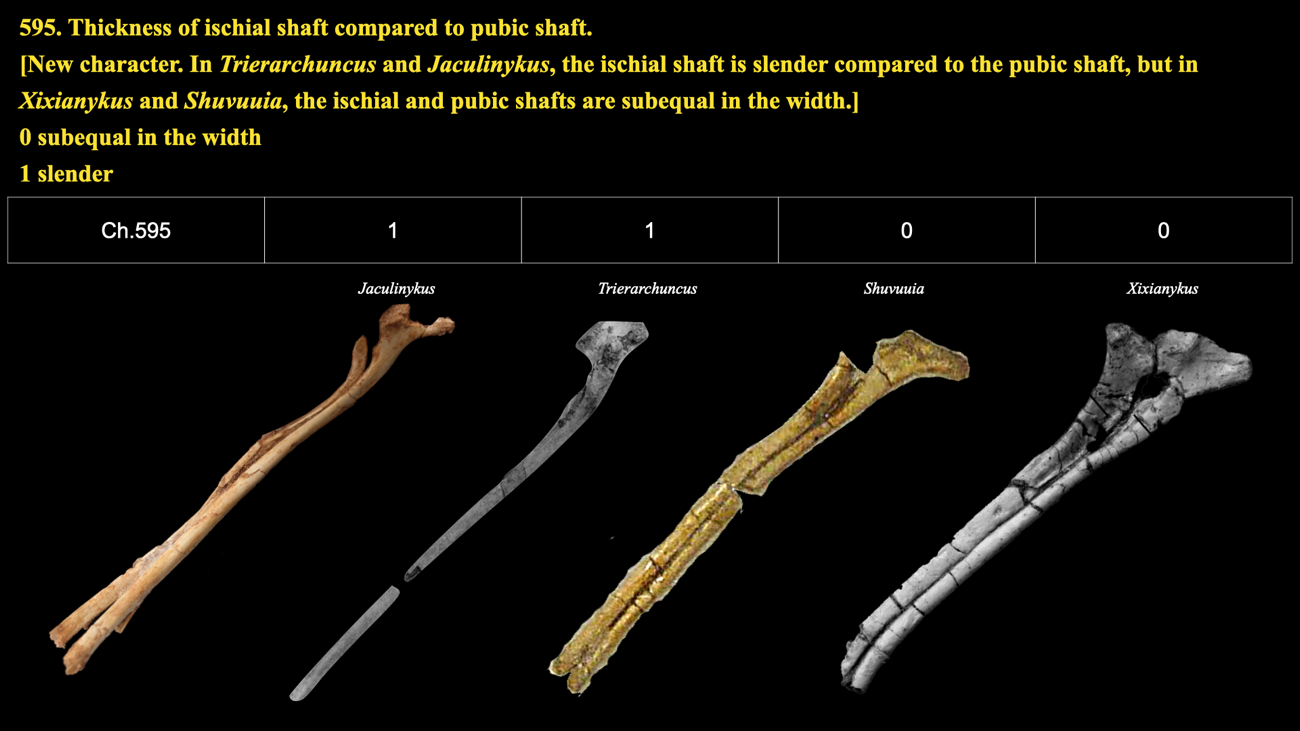


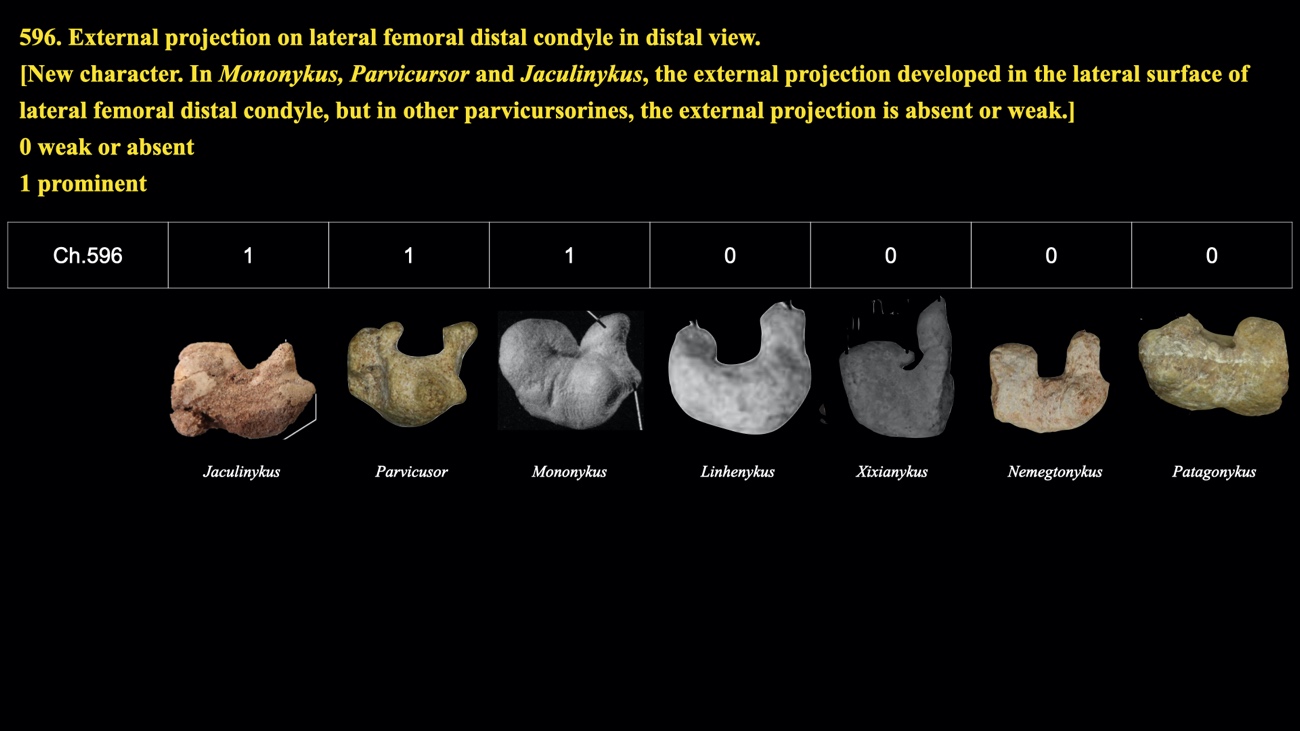


**Data matrix**

Herrerasaurus_ischigualastensis ??000000?00??100???????0010?00000000000????0?00000001?2010000000000?0??01100??00100000????00?0?00?00??00?1????01???11??000?0?0000?0?????0???000????01?1?0?0?0?000????0???????????0???1?000?0?0011???????010??000??????????1010?000?010000000???0?000??00?0000100?0????0010?001000?000000000000000000000100?0?01?1??0?000?00???200000000?000?0000?????????00??????????0??0????00?1000010?001110?10000100??0100?0?00001?0??01000?0?0120000000110000?11100000???0000?0?0??0????00?000001001????00??01010???00000?0101????00?1?1?0???010000?2000000000000000?0000????0?0??100????0010000000?000?0?0?00?0000?000?01000???

Dilophosaurus_wetherilli ??001020?00??0?0????1?????0?010?0010011{0 1}0{0 1}1??0?0?1001?2010001000010?0??1111???0010?0?0???00010?01?0????111????000?011????????0000?0???0?1???000???1?1?0?0?0?10000??101?????0???????1?1?1?0????0???0?10?001010?01??????????1010?000?1000000000??1?10???00?0110110?1101?0011?0011?0??0?000110000200???10?10000????1100?030?00???0?0000020?000?01000?0???10?0???????????0??2000000100???00?0??100?100001000?0100?0??010?01?00?000?1?0020100000110000?1?11000?????11000????0????1?00?0001101????100?00?00???100?00?100????10?????0???11000001000000000?0000??00110?000?0??100????001000000??0?0?10??0??0000?0?2001000???

Coelophysis_bauri ??000011??0??000???????0?00?0101001?1?10???0?00001001????0000000000?0??11100??00100??0???000?0?01?00??0??0?????0???????0?????0000?0???001?????0?????1???0?0?1000?????????????????0?001?000?0?0011???10?100???000??????????1010?010?020000000?????000??00?1??0100?11000?02??0010?0?100??00{0 1}00002001??000????0????00?0?03??00???100000020?000100?01?0??????00??????????0???????001100?000?0?01?0?10000100??0100????000??1?000000?1?0000?000?0?1000??111100?0???001000?0000????100000001101???000?000?00???0001101100????10?1?0?0???1100?0??0?0000000?00101?0?110???0??0?001????001000000??0???10???0?0?0????1001000???

Tawa_hallae ??0001201000??00????01001?11000?00000?1????0?000?0011?200?001000010?0??12100???1100??0???000?0?00?0?0???11010000??0????????????0?020?00?1???000???????000??1??0??????1?????????????10??000?00000?00?100?00???0????0????????00??00002000?0000?0000?000100?0???????1100?00??00?1?????0???0?????0?????????1????1?????0????????0???????0??00????0?0?00????????????????????????????1?10000100?00100110000?????????????0??1????000???10??2?0000?011100001110?000000000000?0???0???0?00??00000100?000?0?110????????10?????????????????????001??2000000??100????????????????????????0001000000000?010??00?00???00?1?????????

Carnotaurus_sastrei ??10000000001000???0??00?010000100010001???1?1001110??200000000?000?10101000???10?1100000001?1101100101000010000??00????0????1000??2??0??????0?00????00?1001??0?0????????????????1?101100??00?01000?100?0100?0??0?11101000101000??01000000?0?0000000???0?000010101101?10?00??11102?1011100?000100?0010?1000010011?110040??11?0??????00????0?????0000???0?????0???00??0000?000?01000011010?0221?0110?2000000?000111000????????????1?0???01?????????????????????11000?0000??????00?00??00??0?000000100????1000000100?000?0?00000???000?10000?0111011??011?00?110?0????????????????????????????????????????????????????

Ceratosaurus_nasicornis ??10100010001100???00?00?0100000001000010001111010101101?1000000001110?11100??0010010000000010?011101010010100010?00?0?00?0??1000?02??0?1???000???0???1?1???10?00???0????????????1?????000????0???0?10?00100?0?0??????100?100??000?11000?0000??0?0?0??00?0000111?11011000000011??0?0011101?000100?00???10000000110110040?01?0?1?000?01000?0?01000010??10000??????????00020000?0100?0?1000?????????????????10001100101????0100??10000010001011100??1??????2????1100??0000????1?00000010010000000000?000101000100100000010?00000?0?11000000000011011000111011110?0?0000?1000???001000011?????????????00?0???????0?0???

Limusaurus_inextricabilis ??1000101001110101?00?000?11?0?1?01?000????00000?000??01??000000000?10012100??00120??0???00000?00?1????0000?0?020?00??????01?000?????????????????????????????????????????????????0?20??00??01?1110??0?1000???0?10?0??0????11??????1???????????????????????000??0?1101?1?2?0000???01?0???0??????????????????00???1????0??????????10000?000???020000000?0??1101000??0?10002000000110000100001?10011011?000?00000?000000?????0000??010001000101{1 2 3}100?00011??02???000000?000110001000?1001001001000000100???0?0000?00???000?0?00?00?1???00?010?0?0?????00011????????????0??10??????1???????001?0???00??0??00???2?00000???

Majungasaurus_crenatissimus ??10100010001000????100000100001000100011100?1011010112010000000010?101?1010??010?1100000011?11011111011000100010?00?0000001?1000012??001?00000001??1?????0???0?0?????0??????????0?10110???0?00?????10?0?100?0100001001000101000000110000000000000000100?0000111010?1?10000?011?02000111010000100100101?0000001010110030???1?001100002000000110000000?100100??????????000?00000???000?0?00022100100120000??????????0?????????????1?0???01???11????????????????010010000010001000?0001001000000100????????????0???0?????????0???????????000000??00100011101101010000111100010000100010??0??0???0000000?0??0?0000011??

Cryolophosaurus_ellioti ???????????????????????????????1??1?1??000??0??0????0?2?????0?00001011??1100??001000?????00000?01?00??10?10??00????10?0?0???????0???????????????0???10101?????000??????????0?????0???????????????????????0?0?110??0???????????????0{1 2}??0?0000?????????????????????10?1{0 1}????00?????0?????0?????0?00?00???10010??0?1?10?0????????00???0????????00?0?0????0?00??????????????????????????????????????0?????????10000?????????????????????????????????????????????????00?????????????0?0??1??10?????000????????00????????00????10??????01001?010??011?0??0000??????????0??????????001100000???????????????????????????????

Monolophosaurus_jiangi ??001000100101?10000?00?01110001000?0000??001111000100201100000001111??11100??0010000000?000110011000?0011000001??010????????0000?????0?1???00??????1010100?100000???????????????0?0011110??0000?00?100?0100?1????0100211?1010000002000?00000000000?010100??1101?10?1?100?01011?00010?100101001000000011?000000?10010030?00?0?????0?02????0?????0?????????????????????????????????????????????????????????????????????????????????????????????????????????????0100100000100100?0?01000010000000000???0101000100100?00110?100?0??????????????????????????????????????????????????????????????????????????????????????

Piatnitzkysaurus_floresi ?????0?????????1??????????1000????110?00100?1??????????????????????????????????????0???????????????0????????????????????????????0?????0?1???0?????10??1?1?0?10?0010?010??????????00????0?10????????????0??????????????????????????0?1?01?00?0??0?0????0100111110?10?1010?1?0011????10??001?10010???????11000????1?00??30??0???0???0?02????0??????????????????????????0??21000?0?0????00?0??1?0?10?00???0??100????0????????????????????????????????????????????0100??0????????????010000??????????1?00????0000?010?????11?1???0???110010?0100010?0100??0??10111???0?0????0??????????????????????????01?0?????????????

Baryonyx_walkeri ??0101201001110??????10?0?11?0?000??00100000101????0??2??????0??0?101??10100??0010011???10?000??1????11???????020?010??????????0000000??1?10010???0?10101000110000?0?10?00?10??0?????11110??0100?00?10010??1111000???0101?103000000?20000010102101000100?1101111?10?1010?001011?00010??01121001100??000110000??0100100?????????0??0002???????????00???01??0?101010???0000????00?00?0?10000?2010?02010001012100110101??????????????????????????0000????0100?????1?0??????????1000???0000100????00?0?????0????0?0100000?100?0000?0?010110??????1000100??????????????0?0100000?00??????????????????????????????????????

Eustreptospondylus_oxiensis ??0??020100?1100????00000?1100??0001001000?0???????1??????????000?1?11011000??0011011?00000010?010?0????010?00000?00?????????0??0?????0?1?00010??01010001?0111?100???10?00?10000??0??1?110??0????0101001?????????????0?????010000002000?0000000101000100?0110111010?11100101011?00?10??00100002000??000110000??01000?030?00?0?0?{0 1}00002???00?00????????????????????????????????1?00?0?100?0?200010001?0000??????????????????????????????????????????????????????10???0??0???01000?000000110000000010000001010000100010?0?01?000?010100100000001000100010000011100000001100000000100011??00??????00000100000?001000???

Afrovenator_abakensis ?????0???????1?101001??0??11010100110?00000?1???????0?201100??0?001010011100??00?????????00010?01??0????010000001?010????????0?0??????????????????????????????????????????????????????????????????????????????????????????????????02000100000???????0100?0110110?10?1?0001?1?11???010??0?{0 1}?10?10???????1?00???????0????????????0???0??0?0?0?100???????11?00??????????????????????????????????0??0?01???????00?????????1???011??1??00???????121000?2???010000000100100??0????00?00010000100?0000000?0?0?01???000100010011010000??0?0?1100010001000100????0?0??????001????00?0000100?111??1?0?110????01?????2???0?????

Allosaurus_fragilis ??0010{0 1}01001010?0000?0000?1000010?110?0100100001?1?011?1?000000000101???{0 1}100???111010000?01000?010?0???01?0100010101120?00010??0001001001?00?000010?00101001100100?1?10??0?0??000000010000??0000?00?10000110?11?000100101010200000021001000000000000010100000111010?1000000101100001001011010000100000010000001010010030?00000000000020000001001000???1?0100??????1000002100001010000000000100000000100000000000000010100001110100000100010121000021110000000001001010001000100000100001000000000101000?1010000100000011010000?00010110001100110010001000101110000?00010000000110001110?1?00110000001010?02001000???

Neovenator_salerii ??00?01010000101000010000?1100010011000101000101110????????????????????????????????????????????????0???????????????????????1?????????????????????????????????????????????????????????1?100??000??00???00??????????????????102100000?100100000000010?010100001111011111100001011?00010??00200000010??001101101??1100100?0?10???????000100000100010?10??100100?????????0002100101?10?0?1010?????????????????????????????????????????????????????????????????????0110100??010001000??000001000??110010100101010000100000011000000?00010120001100110010101010111110010001101001????100?????01??????000?01?10002001000???

Sinraptor_dongi ??001000000000?10000000?010?000110110000?0000001000000200000000100101??11210???110011000?010010011000?00010000010?011300000100001000010?1?00000??10?00101000100100???10??0?0??0?00?1011000??0000?010100?01110010000??010101010000002100?00000000000?010100110111010?10100001011?001100101101001010001001100000101?010030?0000?????0002?00?0??1?1001???1??1??1?0000???0????????1010?00100?0???????0??????????????????????????????0????11001012100?????1000000100100101001???000?0??1000010000000001100??11010000100000011010000?00010?1000110011001000101010111?000000?00?0000001000011000?00110000001010?0100?000???

Acrocanthosaurus_atokensis ???010100?0??101?0??10?0?10??0?1?0110001001000?0?0?0112011??0?00001010??1110???11?0??0???01101111?00??10110??01001?1120?0??1?0??0?????0?1?0?0?????1??0101?0?10000????10??????????0?????200??0000??10???0?101011???0??0?0??101??000021001?00?0?0??1?0???1000?1??1?111110??001?11??0?10????2?1???01??????10?1?0??0??01?0?1??????????0?02??????10?1?0????1?010??????????0???1000?10100?010?0?0100000??10????02??00?101??0?0??011101?000?10001002200002??100?0???00???10??????????????10??????????10?1010??0?0{0 1}0??0????00?11?10?00???01012?1001?011?0??00100?101?????0?0?????0??0??100????001?000?00??0?1010??2?01000???

Giganotosaurus_carolinii ???0?00????????0????10????10???1?001000100??01?1?0????2???????????1010??1110????1?0??????01101111?01??1???????1?0??10{1 2}??????????1????????????0????0???101????????????1?????0?????1?????201??0?0???10???0???10?11????????????1???000200?0?10?0?0??0?0???110001??1?111110??{0 1}?1?11??0?10??0?2?0???0???????10010????1?01??????0???0?000?01??????000???0???1000???????????0??????????10??010?0?????????????????????????????????????????????????????????????????????0???10000?110?1??0?0?000?11?????10?1010??010100?0???????11?1???????01012?100??01??0?????0??10111???0?0??1?00????1?????????????????????????????????????

Ornitholestes_hermanni ??00?0000?01?00101011100??10000?01101000000000?0?001110001100000010?10011100??00101?0100?10000?000201000?001000000011{2 3}0????100000???0?0?????0?01001111101?100??????????????????????0010?10??0000?00?100?00?0?11100011010001012011102101?001000000?010100?????????10?1?101200011??111100?11?1001??0001001??100001010110?0???10?0?10?0020000?10?0??????????1?1?????????????????????????????0?10?11000010001?0??0?0?0?0?????0??????????????0??????????????????????1?0101??0100000?0?0010011000000?10????010?01000010?000001?10000?0????????????10?0?10?????????????????00??0?????????????001??????0?100?0?0?0?0010?0???

Zuolong_salleei ??00??00?00?010101001?0?0?0?0000001110000000??????00???????0??010?0?0??1?10111011????00??00000?0?1100?00?100000001011300??0????0??????????????????????????????????????????????????????????????????????????????????????????10100001020?0000100???????010100???????10?0?101100011??0?100???00?001???????????????????????40?001?000??000000??0?00????????????????????????????????1?10???1???0?10001???0100???????00?000?????????????????????????????????????0???00100??????????00?0???0???100?00000011?0010112?00???????????????????1?011000100001001000001010111?0???011??000????????????010????100010101000????0?????

Albertosaurus_sarcophagus ??1????0000011??0????10?1???0??0??0100000101110?11??002011001100011111011201??000?12000010111100112010101?00101010010300000100000000000?1?00010?01??1000?00110000100?10??00???0000?001??00??0?00?00?10000101011?011100???010??00?10?00??0?0000000?0001010???011?0???????0000011?00??????0?0?00?????????1??000??0?00?0041?0?00???10??0??00??0??0?00?0????0??0000001??000020000???00?0??0000?11?001????????00??0?00??01000000010??0???????????3200?0????0000????????10101?100000000?20???1000000?0010??0001???00010000000102000101?1101100110000100100000001?1110011001?1000100?2000110?001?0011000001111000?0?1000???

Daspletosaurus_torosus ??100020100111110100??00?10?00000101?000???11101??????2??0001100011111011101100011?01001?000?0?011201010110?1?101001030???01?0000????????????0?00????????????????100?10??0?10?0000???1?000?0000??00?1000010101????????211?10100001020000000000000000010100??????????????0???0????0???????????????????????????????????0????????????????????????????????1?111?????????????2??????????????????????01??????????????????????????????????????????????0?????????????0????????????????????20???????????????????????????????????????????????01?0002???1?0?10?????01?111???2??????????????????????1????????????1????????0?0???

Dilong_paradoxus 0?100020000110?101001100?10?00000011100????010210100012011001100000?11011100??0010001100000000?001200?0001?00010??011??0?????0000010010?1?0000000?????1?1??1??0001??0????????????0?0?11000?00000?00?0?0000?101111111101010101000010200000000000100000100?0???????10?1?100000010??0010???0?01001?????????????????1????0??????????????0?0??????2?000?0??1??????????????0????????1?10?0000?01?1???10001????????????????????????1??1??10?110010022?0??21????00000??1?0??1??010000??0??20000100?0?0???11??????????????0??????0??0????11?01?0001?00??0?10??0?001?111??010???10???0002000111??01???????0?00?0??0?2???000???

Gorgosaurus_libratus ??101020100011?101001100110?000000010000???1110?11101?001100110001111?11110????0??1210000001?0?00?20101011??10?0?????3?0000100000000??0?1?0000000?????????????000????????????????0?00??000?00000?00?1000010101110?0??0????10100001020000000000000000000?????????0???????0?0??????00??110??0??0000000?0?????0000?1????0???????????????????????????000??????????????10??????????1??????0?????????????????????????????????????????????????02???32?????????????????100101011100????0?12100?100?000?00101?0001???00?10010?0010????????????????????????????00???????????0?0?10???????????????01000110??????1????????0?0???

Guanlong_wucaii ??10100000011111010011001111000000111000010010110001012011000100000?11011100??0010001000?00000?0012010001101101200011300?10??000000000011000000001101000100011000100010?1001000000?001?000?00000?010100?00?10111??1??0????101000010200{0 1}1001000?000000100?1??????010?10101000010?1001000010010010000000010010001110010030?0010?00100000000?0??2000000?????11??????????00020000?1?10000000?0110011000010001110001000001010000010?10010011001002100002121000????00100101010100000000021001100100001010110001000000100000000?100010011?01100011001100100000101111100?2000?1?00100011001111001?00110000001010002101000???

Kileskus_aristotocus ??10??00?00?0?11000011000?0?00??011110000100??????01????????????????????????????????????????????????????????????????????????????????????????????????????????????????????????????????????????????????????01?10?01???????????011000102100000000???????010??0????????????????????????????????????????????????????????????????????????????????????????????????????????????????????????????????????????????????????????????????????????000?????????????????????????????????????????????????????????????????????????????????????????????????????????????????????????????????????????????????????00??????001?1?????????????

Proceratosaurus_bradleyi ??10??000000011101001100111000?001110000???0??1???010?001100010000?????01?0????01???????????00??0??????0110?0000???112?0??0??0?00011010?1??011????????????????0??????????????????0?001?000?00001000?100000?10111100??0???01010000102101?0010000000010100?0??????????????????????????????????????????????????????????????????????????????????????????????????????????????????????????????????????????????????????????????????????????????????????????????????????????????????????????????????????????????????????????????????????????????????????????????????????????????????????????????????????????????????????????

Juratyrant_langhami ?????????????????????????????????????????????????????????????????????????????????????????????????????????????????????????????????????????????????????????????????????????????????????????????????????????????????????????????????????????????????????????????????10?1?100?00011?00???0?0010000100000??1?0???0010?00??030?001000???000????00???????0???1???????????????????????????????????????????????????????????????????????????????????????????????????????0100110??1100?0100??21001100?00000011?1000?00001010001000001?0?101????????0????11001010101010110001??0????????????????????????????????????????????????

Tanycolagreus_topwilsoni ??1??000?00?00????????????????????????????1?0000???0???????0??00000?101122011000100??0???00?11001?????????????0000010?????????????????????????????????????????????????????????????????????????????????????????1000????101??01??0?10??0??000?0????????????011???1??0???1????????????????0?0?000100??????100110??0?001?0?0??????????00?0???????20?00????1??????????????000200000111000000000110111000110001110000000101?11000010?100000110000022100020?100000000???????????????????????????????????11110101???00??????????????????1010110001100010010000010101110001000110001000200011110010001000000010?0002001000???

Tarbosaurus_bataar ??0???00000011010000?100110?000000??0000????110010100120110011000?111101???100001??01000?0?1010?112?111011001010??01031000010000?000??0?1?10010001?000001001??0000???????0??????0??0011?00??0000?00?100001?101110111001010101000010200??0?0000000?00010??????????????????????????????????????0???????????????????????0?????????????????????????????????????????????????????????0????0??????????0?????????????????????????????0???1?0???02???32?0???????????????????????????????????????????????????????????????????????????????????????????????????????????????????????????????????????01?????1??????1??????000?0???

Tyrannosaurus_rex ??10?0000000110101011100110?00000001000001011101101000201100110001111101110110?111101000?011?1001120111011001010100103?000010000001001001?10?0000100000010010?0?010?010??0?100000000011000?00000?00?10000101011?011100101010100001020000000000000000010100000111010?11?00000011?0001011001000000000000010000000010010031?00000?0100000100000020000000?100110??????10100020000010100000000001100010012??00010001001001?0?000010?101000??????032000021??00000?00010010101110000000?12000110000001001011000101000010000000102100101011012001210010001000100010111?0010?0??0?0100020001101001?0011100001?110002000000???

Stokesosaurus_clevelandi ??????????????????????????????????????????????????????????????????????????????????????????????????????????????????????????????????????????????????????????????????????????????????????????????????????????????????????????????????????????????????????????????????????????????????????????????????????????????????????????????????????????????????????????????????????????????????????????????????????????????????????????????????????????????????????????????010010101110010100??21001100100010????????????????????????????????????????????????????????????????????????????????????????????????????????????????????

Xiongguanlong_baimoensis ??10?000??0?1?1?0????100110??0??011?000001?0102001??0?0011001100010?1?1??100???0111??0?00001???0?120111011??1000000103?001?1?0000000???1100000???0??10001000110000????????????????????????????????????????????????????????101??01102100000000???????0100?0000111010?1?10000001000001001000000010?100100100110?10100100????????????????????????????????????????????????????????????????????????????????????????????????????????????????????????????????????????0100??1??1100?000001210??10000000??????????????????????????????????1100100011001100100????????????????????????????????????????????????????????????????

Compsognathus_longipes ??00?011000????1010?1?00??1101000???0000???000000000??00001000000?0?0??111?0????1?1101???000?0?001000?0?0??????0???????????????0????????1????????????????010??0????????????????????0011000??0?00?00?0?000100??0?000??1210?101000100210110010000000000100?0??0??1010?1?0?0?00010??0?1???0?10?0?200?00???????10??0010?103?????0??0000?0?000?01000010000?1?1000??????1??00020000?1110???000000?0??1???1?00??01???000000????0?01011???00?1000?1?22???12??10000011001??0?1000???0???0??0?0???????00?001111???00100001000100010100?0??1??????000??1????1???00?0??????00?0??????0?0001000?10?001?0011000?00?0?0?02001000???

Huaxiagnathus_orientalis ??0???000001?0?10100??00??11000???11??0????0000?1000?????110?0000?0?0???1?0????1???1?????????0??0??0????0??????????????????????0?????????????????????????????????????????????????0?001?000?00?00?00?0?0???????????????????10??0??00?0?1?00100000000?0100?0??????0????????????11??10????0??0?0?100??????????10???01??10????????????0????00?0?120?1000??1?0000??????10000020000?1110?00000000100???????????00????00?00?0100?001???00?0?10?0?1?22???12111?000000?01?00?10001010???0??0?0??02??000?1011?????????000100000001010000??1??????0?????????????0????????????0???0????00?2000110?001?0011?0??00?0???020??000???

Sinosauropteryx_prima 000000000001?001??0?0?????0?0000????0?0????0000???????????100????00?0???1100????11????????0??0?00??????00??00012?0?10??????????0??????????????????????0???????0??????????????????0?001?000?00?0??0??0?0?0?????????????????10100?1002101?0?1000000?000100?0??????010?1?1?0?10?10??11????0?20???100?00???????1000?01???03?????????000?0?000??1120?10000?1?1110?????????00020000?1110000100001200?00?00?????01?0??00000?1100?0111110?1??11?010?22???121110100010001000?1000???0???0??0?001020?000?1011?????1???000100000011?100?0??1??01?0001??0??0?1????0?01??10?00?0???0??0?00?20001101001?001100??00?0??0020??000???

Nqwebasaurus_thwazi ????????2??????1000??1011?0?00??00?1???????0??????????????????????0?10?1?2011101100201001??0????010?0?0???????0?00?10????????????????????????????010?00?101???0000????????????????????????????????????????????????????????????????03?11?21211?????????00?0???????10?1??01200?0??11111???100100??????????????????????????????????????????????????0???????0????????????1002100001?10000000??0100?100?0100?10??0?00000010?000000101001001000101221001111001010110???????????????????????????????????1???0?0????00??????????????????1??0??1?????000001000001011111?0010?0010?0?0002001110?001?0011000000001000?0101010??

Anserimimus_planinychus ???????????????????????????????????????????????????????????????????????????????????????????????????????????????????????????????0??????????????????????????????0???????????????????????????????????????????????????????????1???????????????????????????????????????????????????????????????????????????????0????????????????????????????????????????????????0?????????111200000????0100000?012???????2???????????????10000?1010?01?1001001?002210??111110012210????10100?100?11?0???????1010000?0010????00???00?1000100010??000????????????????????????????????????????????????????????001?1????00?010110???0??1?????

Archaeornithomimus_asiaticus ??????????????????????????????????????????????????????????????????????????????????????????????????????????????????????????????????????????????????????????????????????????????????????????????????????0?????????????????????????????????????????????????????????00????20220011000121100000000020000000010000000000010031?1010?0?1000020000000?00??0?????0????????????101210000111001?1000?00200100012000010000000000????001010?11?100110110022??011???00011210010?0?0000100010000100000100010??00101100011?01001010100010000010010100100011011000100000101111100010?0????100002000110100101???100010011000?0??1?1???

Beishanlong_grandis ????????????????????????????????????????????????????????????????????????????????????????????????????????????????????????????????????????????????????????????????????????????????????????????????????????????????????????????????????????????????????????????????????????????????????1????????????????????????????????????????????????????????????????????1???????????1112100001?00010000?0002????000100000000????000??????????????????????????????????000002?0?????????????????????????????????????????????????????1????????????10?0??00021?0110?10001010???1100010?0?1010100?1000110???1?001?????00??10????001011??

Gallimimus_bullatus ??0???00210000000101?101110?0?000???000????00000000?11001?110?000?0?0??1???111011200010000?0?0??000?0?001?010112??00???01?010?10?0?110??1?0000100010?0001001??00011??110?0?100010??10?0??10?1010?00?0?0?01?10?1?1000?0???011??????1??????????2??????????????10??010???202210010??12?????0?0?????????00?1??000??0??0?0040?1??0???100?0??00??0??0?01?0????0??0?????????011200000??10?1??0000?02?0?0???2??0000??0000??1?0??001010??10????????0?2????0??2?00011010?1?010100?100011?0??0????1010000?0010??01001??000100010001010000?110?????00?1001?0????00?00??????00???00?0?0100??0001???00101???100?01?1?010?0001010??

Garudimimus_brevipes ??0000002?000001001?0101110?00000111000????0?0000000??01?0110000010?0??111011101120?0100?000?0?00?000?00?00?00?001?113?01?01001000?11?0????00010001?000010?01100?11?????????0???00????0001001?10?00?000?00?1001?1000?0?1??11??????1??????????2????????????000??1?????????????0????2???000001001000001001???000000001?040?10100?0??0000???00?0??0??0???????????????????????????????????????????????????????????????????????????????????????????????????????????010011100010011100?10010010010001001001000101020??????????????????10101100011001000110010001011100000?0100?010002000110?00100011100000?1101020000011??

Harpymimus_okladnikovi ??00000021000?010?0??1011?0?000?0?11100????000000000????????0???????0??1110111?112010?00?000?0?00?000?00100100?????????????????0?????????????????????????????????????????????????0?0010001001011000?0000010100??0000000?0011??????1?????????11?0??0????????????1010????0??0000???02?0???0001?02??000?00????00??110?11040?1?10???1000020000?0000001????0?0110??????????11??????1?10?1?100?0?12?0?000120000010000000011?10000010?10010011001102210002111000111100100101001????11?0??0??00100?000000????010????00???????????????????0101?00???0?????????????????????10????????0001000110?00101???100000?0?000?0001011??

Ornithomimus_edmontonicus ??0?????210000???11???01????0?020?????????????00???011?0111100000?0?0??????1111112?0010000???0??00?????010010101??011??????????0?0?11???1?00?????0???000?0?0??0?0???1110?0?10?010??10?0??10?1010?00?00??01?10?1?111??0???011??????1??????????2??????????????10??010??1??22100???102?????00??00???0??1??1??0?0??0??0?0040?1?00???100?0??0???0??0?01?00???0??0?????????11121000???00?10?0000?02?0?????2?????0??0000??1?0?0001??0??1?????????0?22???0??2?00012210???010100?10001100???????1010000?0010??010????00010??10001010000?110?????00?1001?0?10?0???0??????00???0??0?0100??0001???001?????100?01?1?0?0?0?01000??

Pelecanimimus_polyodon ??00000021000101000111011?0?00010011100????00000?00000001111?000??0?0??1120111011202?1?0?000?0?00???????????????0?010?00???1??10????????1?00?01????????????1???01????1?????10????0??00?001000?1??00?0?00010100??1?0??0????1030001103201?102110201010??10?0???101010?1?????00010?1???1?0000?1??????00?????????????????0??????????????????????????00?110??????000001???1????????1?10?10000?0??200?0001?000?0100?0000011??00?1010?01?10?110110?221000112100011210?????????????????????????????????????????????????????????????0????????????????????????????????????????????????????????????????????????????????????????

Shenzhousaurus_orientalis ??00000021000001000??1011?0?000?0011000????0000010001?????1???????0?0??1120111?1????0?00?0?0?????00?0?0???0?01????0????0???1???0??????????????????????????????0??????????????????0?001?001000010?00?0?000??1??1?0????0?????1??????1???1?????11001001??00?0?????????????????0???????????0?00???200?000??????00???0001?041?1??0??????000?0000?0??0???0??0?01???????????????????????????????????????????????????????????????????????????01??10?2210???1210001011001000?100010101000???1000100?00010010010100?1?010100000000010000????1????001100??00100????????????????????????????????????????????????????????????????

Struthiomimus_altus ??0???0021000??1?10?110?110?0000011?000?????0000100?11???1110?00010?0??11?01111?120001000100?0??000?0?0010010112?1011??01?0???1??0????0?1000?????0??0000??0???0?010??110?0?100010?000?0001001011000?00??01010?1?1010?0???011??????1??????????2??????????????10??010??????2100???002?????000??????0?????1??000??0??0?0040?1?00???100?0??00??0??1?01?0????0?10?????????111210000??10010?000000200100012000001000000001?0?0001010?01010111011002210001?2?000112100100101???10001100?10????1010000000100100001?0000100010001010000?110?01?00011001?0010?0?000???1100010?0?10?0100?200011??00101???100?01011010?0001010??

Achillesaurus_manazzonei ???????????????????????????????????????????????????????????????????????????????????????????????????????????????????????????????????????????????????????????????????????????????????????????????????????????????????????????????????????????????????????????????????????????????????????????????????????????????????????0?2?0??00??1121???10???????????0?0?????????????????????????????????????????????????????????????????????????????????????????????????????010???????0???01102?0????111????0??????????????????????????????????????211???0?????????????????????10????????100{1 2}001110?00??????0??0?0?010????????????

Albertonykus_borealis ??????????????????????????????????????????????????????????????????????????????????????????????????????????????????????????????????????????????????????????????????????????????????????????????????????????????????????????????????????????????????????????????????????????????????????????????????????????????????????????0??????????????????????????????????????????????????????????????????????????????02101011??1????????????????????????????1?????1?011?12???????????????????????????????????????????????????????????????????????????????????????000?111111001????????????????????????????????02?01????????0????

Albinykus_baatar ??????????????????????????????????????????????????????????????????????????????????????????????????????????????????????????????????????????????????????????????????????????????????????????????????????????????????????????????????????????????????????????????????????????????????????????????????????????????????????????????????????????????????????????????????????????????????????????????????????????????????????????????????????????????????????????????01????????????1??12?0???????1?00??????????????????????????????????1????????????????????????????????01???10010?11?11111??11100011000002?110002010000???

Alvarezsaurus_calvoi ?????????????????????????????????????????????????????????????????????????????????????????????????????????????????????????????????????????????????????????????????????????????????????????????????????????????????????????????????????????????????????????????????1????00?0?0?0?00121011000?1??????????????????????????30020????????12100?0?1002???????????1???????????000?00001?0010??000?0???????????????????????????????????????????????????????????1?011?1101000?10020??0110021002001101?00???????????????????????????????????00?11?1??001????????????????????20????????1011??11111001??????00?00?01110??10000???

Aorun_zhaoi ??00??00?10?110101001100??0?00010011100????00000?0000?2000100000000?0??1110110?1100??0???100?0?00??????010??0?0000?11????????0?0?????????????????????????????????????????????????0?10??010?00000?00?0?0?01?0?1??000??111?01010001002001?0010001100000100?0???????1101?101001?11010110000?00?00?00000000??0?000??00????????????????0010???????????????????????????????????????????????????????????????????1??0??00???1?10000110?10110111?0?00221001212101000110?????????????????????????????????????11???????????????????????????????????????????????00000???1000020?0??????000?100110?00100011?00000?01000?001000???

Bannykus_wulatensis ????????????????????????????????????????????????????????????????????????????????????????????????????????????????????????????????????????1?01???????????????00?0001????????????????????1?????????????????01011????????????????????????????????????????????????????1{0 1}01??111010100?1?111????1????????????????????????????0??0?1????001??00???1?00?0100??0100????????????111100001?0?0010001012000002100101102110110?0?1?11000111110111011111002201112???01000111010???????????0010010????11000000?????????????????????????????????11000100010000000111000101111110020110??00000010?111?10010????1000000010002?01??????

Bonapartenykus_ultimus ???????????????????????????????????????????????????????????????????????????????????????????????????????????????????????????????????????????????????????????????????????????????????????????????????????????????????????????????????????????????????????????????????????????????????????0?0??00?0???????100110101101??????????????????????????????????????????????????1110?11101?01001?001??????????????????????????????????????????????????????????????????????1????????110????????????????????1111??0?1??????????????????????????????12????????????????????????????????????????????????????????????????????????????

Ceratonykus_oculatus ??????????????????????????????????????????????????????????11?01?1?0????1110111?1120????0?100?0?00??????100??0?????10???????????0?021????0??001?????????????00?0000??1??????????????10????????????0??0?0??????????????????????????????????????????????????????????0?????0???10??0?221??1???????????????????????????????????????????01?1??????????????????????1011?????1?00?0000??????????????0????10????????????????????????1???????????????0???11?2?????????????????????????????????????????????1???????????????????????????????1???????????0???????????????????????????????11211?110??010?????00102??1001??????????

Haplocheirus_sollers ??00??0011011111010111001110000100111000?0?0000100001?01?1100000000?0??1000111?112010000?10000?00??00??010011000??010?00110100000021?0001?0001000010?0111??00?00110??????1?110??00?1011110?0000100100?0001111010001??02100101000100120110010001101000100?0??????00????101?00?10?10111?101001001??0100001??1??0??1?101030?00?0?0?{0 1}?00??00100?00?00000??1?01???????????110200000111?100000101100110110110100?00?1100001?1000000101011011101100220111212101010001010???????11??01002?000001001??000011010100??01001100000110110?0?01??0110001000000?1010001011110?00?011010??000?20???1?10010?????00?00?010002001000???

Linhenykus_monodactylus ?????????????????????????????????????????????????????????????????????????????????????????????????????????????????????????????????????????????????????????????????????????????????????????????????????????????????????????????????????????????????????????????????00?1??0??110????1210????021212????????????00???0?10?0?{0 1}?20?10??10?121?00???0020????????1???1011?0??????????????????????????????????????????????????1?10101111010111111?210032111?2???11011?120??????????????????????????????????????????????????????????????????0??1?1???0010???11??0????0111???11?????????112??0?1?1?01?00110?0002??1000?????0???0

Mononykus_olecranus ?????????????????????????????????????????????????????????????????????????????????????????????????????????????????????????????????????????????????????1100?????????????1001011??0??1???????????????????????????????????????????????????1?212?1??0??????10?0???????0????1010?11????1211?101021012001000000??10100???1010???2??1?????0121?0?1??????????????????101100???1000?00001?11001100000230000101111?0021000111011?10101111010110101111002211112??111011?120?0?0????????????????1?00111??00?31???????1???00?0?0000????????0??10011112?20000010111000011011110011?11???1011??1101101001?0011000102?11000?010100??1

Parvicursor_remotus ??????????????????????????????????????????????????????????????????????????????????????????????????????????????????????????????????????????????????????????????????????????????????????????????????????????????????????????????????????????????????????????????????????????????????????????2?21200?00???0?0?0100?0010?????2??1?????0121???10?0??????????0????????????????????????????????????????????????????????????????????????????????????????????????????????0???????0???????2?01?001111?00?3?????1??????0000?0000????1?00???1?011112?200100001110000111111100111001?0101112110110100100???000102?11001?011000?01

Patagonykus_puertai ??????????????????????????????????????????????????????????????????????????????????????????????????????????????????????????????????????????????????????????????0????????????????????1???????????????????????????????????????????????????????????????????????????????????????????????????0?00?001?0?01???10000?01???1???30?2011000??012100?002?????????????????????0???1000?1110????????001????0??011?1111002010011???????00?10??101?????????1??11112?????011?11010???????110???????00100111????02?11011???01001???????????????0?0??????1112000100011100001111111??10111??000010{1 2}001100110??????1????000??????010?0??0

Shuvuuia_deserti ??000000110000010101?101102100000?11000????00000000011000?11001?1?100??12201110112020000?10000?001000?0100000000??10???01?1200000021110101??01001011111?00100?0010???11001011110001100?001000001000?0?0000?0?010011110??001????0??03201?212110202?10??10?0??10????0?10100211110?1121011?102?01???000???0??10000?001?1050?20?1?0010012100110100200101????1110101100???1000?0000111?001000000230000101?11?002????110?11?101011110?011010112??02211112???1101121201000?10020??0111021012001111000?31????1??11?00000?0000????22000?010011112?200100101???0??1???101?001??????101112110110100100011000102?11000?011100?0?

Tugulusaurus_faciles ???????????????????????????????????????????????????????????????????????????????????????????????????????????????????????????????????????????????????????????????????????????????????????????????????????????????????????????????????????????????????????????????????????????????????????????????????????????????????????????????????0??00??????0?????????????????????????????????????????????????????????????????????????00?1111101????????????011?2???0?000?01??????????????????????????????????????????????????????????????????10100000???0000001010001011111???20????????001010?1101??????????????????????????????

Xixianykus_zhangi ???????????????????????????????????????????????????????????????????????????????????????????????????????????????????????????????????????????????????????????????????????????????????????????????????????????????????????????????????????????????????????????????????????????????????????0??2?01??0??????0?0100???00100050?2211?10???101????????????????????????????????????????????????????????????????????????????????????????????????????????????????????????01000??0020??0110??10110110111???31????1???0100000?0000????220?10010010112?20010010111000011111110011???100101111111110?1110????0?00?2??10?0????????00

Xiyunykus_pengi ????????????????????????????????????????????????????????????????????????????????????1010?????????????????????????????20???0?????00201101100111011110?00110001000011?????1?0110?0??1??????????????010??00010110100100?????????????????????????02???0????????????1?1{0 1}010001101010001?1?11000100010?0?11001001???1???1100????????????001000?000000??????????????????????111100000110?10010010110010020001011?21101?????????????????????????????????????????????????????????????????????????????????????????????????????????????????1????????????0000101000101111110020110??000?0??1?????1001?001100?0000010????0?0?0???

Qiupanykus_zhangi ?????????????????????????????????????????????????????????????????????????????????????????????????????????????????????????????????????????????????????????????????????????????????????????????????????????????????????????????????????????????????????????????????0????0???0?10???1????????????????????????????????????4002??????{0 1}?012100?0100?0???????????????????????????????????????????????????????????????????????????????????????????????????????????????0?????????0???001????2100111????0?1????1??????00??????????????????1?011112?20010000111?0001101110001?100???1?101?1???1010010????000102?11000????0?0???

Nemegtonykus_citus ?????????????????????????????????????????????????????????????????????????????????????????????????????????????????????????????????????????????????????????????????????????????????????????????????????????????????????????????????????????????????????????????????????????????????????????02?00100?00???0?01??0????00???01??????0??012100{0 1}1000{1 2}0????0??0?01???????????1000?00001110000?010?0???????????????????????????????????????????????????????????????????0100?????20??00000210210010110???3????????????????????????????????1?0111????0000??011100001111111001110?10???111111101011010????0001?????000????0?0???

Ondogurvel_alifanovi ???????????????????????????????????????????????????????????????????????????????????????????????????????????????????????????????????????????????????????????????????????????????????????????????????????????????????????????????????????????????????????????????????????????????????????????????????????????????????????0??????????????????????????????????????????????????????????????????????????????????????????????1?10111???01?0101121?0?2111?2?????????????????????0??????????????10??????3?0???1???0100000?0000???????????1?????12?2001????????000?1??11100?100010????1??????????1100??1??0??????0?????0????0?

Khulsanurus_magnificus ?????????????????????????????????????????????????????????????????????????????????????????????????????????????????????????????????????????????????????????????????????????????????????????????????????????????????????????????????????????????????????????????????0?????0????0??0?{1 2}2???????????????????????????????????????????????01?????00?0??0?????????????????????1000?000?1?1100?00?000?30???101?????????????????????????????????????????????????????????????????????????????????????????????11?101?0???????????????????????????????????????????????????????????????????????????????????????????????????????????

Dzharaonyx_eski ???????????????????????????????????????????????????????????????????????????????????????????????????????????????????????????????????????????????????????????????????????????????????????????????????????????????????????????????????????????????????????????????????????????????????????0?02?01200?00???0??????????10???????????????1??0????1??2????????????????????????????????????????????2????0101111100?????11??1??????111?0101111??????0?2111?2???1?01???{1 2}?????????????????????????????????31???????????????????????????????????????????????????????????????????????????????????????1??????0?1?2????????1?0?0???

Jaculinykus_yaruui ??000000110?00????????0??????????????0??????????????11000?11001?1???????????????120?0000?????????1000?01?0000000??10???01?12??????????010?100????011111?00100??????????????????????100?00100000?000?0?00?0?0?010011??0??0?1???????0??01?212?10202?10??10?01110?1110?10200211110?1121011??0?10??00000?000??1?000?00??1050120????010012100?1010020?????????????????????1000?0000111?0010000002300001?111110021000111011?101011111101111??????03211112???1101121201000?10020??0111021012001111000?310???1???1?00000?0000????22000?010011112?20010100111000011011110011111100101012110110100100011000102?11000?011100?11

Alxasaurus_elesitaiensis ???????????????????????????????????????0?????????????????????????????????????????????????????????????????????????????????????????????????????????????????????????????????????????????1?00110101??10?101?0?????????????????????????????00110?102110001010?0?????????????????0???????????0?00?001001??0011??000??01001004??1??0??????????01??00?2?01??????0000??????????0????????????0?????0?1000100111001101????00??0?100010000?00?0??1100101220??02???00100100???110100?110101?0???????0???000?20???????1?????011110?1?1?220?0????????111?00???0??0?10???????????????1?????????????????00?010??00?00???0?0?001?0????

Beipiaosaurus_inexpectus ??????????????????????????????????????????????????????????????????????????????????????????????????????????????????????????????????????????????????????????????0??????????????????0???1?00???1?1??10?101??100??????????????11??????0220001100132010001010?0???????????????????????1?????0????????0????????????????????0?????????????????????????????0???????????????001002000001?10?0?000?0?100??????????10????????0????00100?????0?0??????0?22??????1?00?0000?01011110011??????1????????????????????????0???????????????0???????1??????00???0?0?0??0????0?????010????????10?0??0??????001???11??????????????????0???

Erlikosaurus_andrewsi ??100000100110000??0?101110?00001101000????0?00????11????1000001010?0???1100???110?10000010000?00100??0000010000?0??0???1012100001???01?0????00?00?1000000000???00?????????10??000?1010?011?1011010?101000?0?11?0000?0100011??????0220001?0013111?001010????????????????????????????????????????????????????????????????????????????1??????????????????????????????????????????????????????10?????????????????????????????????????????????????????????????????????????????????????????????????????????????????????????????????????????????????????????????????????????????????????????0????????0??0??????????0??????

Falcarius_utahensis ??????????????????????0???????????010??001?0???????????????????????????????????????100000?0?00?00??0??????????1?10010???????????????1?00??00?00??1?01000?000??0?011?110?10011010??0??1?00100000??00?1010??????????????????????????0?20001{0 1}00101110001110?0???????1101?212100010?11211001120100200100110100101010?0010041?101000?1000120010011000010???101111?????010110020001?1?100001001?11001?00100001111001100?000110010010?1010001100110220000211100100000010111100111010101?00000011010??11010010101010001121023001022000?010?01101011000000100000101111100010?11??010000100011010001001100??001000???001000???

Nothronychus_graffami ??????????????????????????????????????????????????????????????????????????????????????????????????????????????????????????????????????????????????????????????????????????????????????????????????????????????????????????????????????????????????????????????????????212?????????2??????20?00?001??111?????????10???04??10?0?0?2010??10?0??0?000100?????00???????10100?2????00???0?0100000110?1?1111001??100?100000?????????0?????0????????22?????????000000001011120011??1???1?0??100???1100?10101000111??2?0121?0?11102?10???0??112111100??00?000?0010110???111??1101?0??0?2000?1??0001010?0000001?0110?0?0??0???

Segnosaurus_galboensis ??????????????????????????????????????????????????????????????????????????????????????????????????????????????????????????????????????????????????????????????1????????????????????0010?????1?1??1??101??0?0??1?000??0???0????????0???001?0?10111?00101???????????????????????????2??????????????????????????????????????1??0????????????????????????????????????????2??????????10?????1???11?0?0??1100110??????????????????????????????????22????????00???????1011110011102010000101000?01000?201010????01000011110?1?102?101??0??????????????0?0??1??0???????11???11?????0??10000????001010??00000??01102001??????

Nothronychus_mckinleyi ?????????????????????????????????????????????????????????????????????????????????????????????????????????????????????????????????????????????????0?1??00?0?0??1??????1??1??10?10??0???????????????????????????????????????????????0???00110?101?1???1010?0???????110??2??010011?11?????112010??001???11100100??010??00????????????100????0????????????????????????10??????????1?0??0?110?0?1101?011110?1101000100??0????????????0?????????0??????????????0000????1????????????0???????????????12???????????????12110?111021100?00???????????0????????0??0??????11??111?1?10?????????????????0??0????????????????????

Avimimus_portentosus ??????????1?1?????????????????????????????????????????????11??1?????????????????????0000?????0??01??????0?00?000???11????????????1?1?0??1?10?00??01??120?0?0??0?00??110?????????0??00????11?????????11??10?0??0?00???????111??????????????2??2??????????????110?111010012000110?11211100100100100000111100000?0000011060?1010?0?2?0000???00??????????????????????????200200000110010000100011001001010000100100000001?111????0?????1??????0??2????????????????0100??1???1000011101010001111?00?00100100011?00011110230010?0000?011011210110100000101000001001100011?11100010112000110111100?11000102?110102010100???

Caudipteryx_zhoui 100000??0?111?0?0000????????0?0?0?11??????????00???01?????000?01?0??????1?0101?10??10000010??0?00??0???00????000??0????????????1??????????????????????????????0??????????????????0?201?001101111100?110?1??0????0?0?????0?10???1101??????12??2????01???0????????0?0?????1??00??????????0?{0 1}0????00????????????????????04??1??0???2100??11????0??00?0???1?011?0?00??11000???????1?00?001?0?0?????1??10?00??0000?0??0001?110?0000??0?1?111?010?22???021?100?000000100101001???0?1?10?0?000?????00?????????01???2011110??00102?0?0??100?111111?0?????10??00?????????011???10????01200011?1001??0????01?1?0???1?0??000???

Chirostenotes_pergracilis ??1????????1?00??????1101????????????????????????????????????????????????????????????????????????????????????????????????????????1?0100???01?1???0???120?011??0?00?1?110??????1????10?0?01111111100?100?10?0??0??00??0????????????1??????????2???????????????????110??211??0?????12?????1????????0??10?1??1???????????52?10????0??1?0?????????????0??????????????????20?200000?????0??10????????????????????????????????????????00?0????0???22000???????10000??1000?100010000??1??010000?000000??12??00?????201111023001121000?01????0?0??????0?0?11??0?0??????00??????????00?????1???0?1??0???00?01?010?0?0??00????

Citipati_osmolskae ??1000000010100101?0?110100?00020111000????0000100101?0010100001000?1101100101?10?1111000100?0?001200?000?000000??010?011011111101?0??001?01010?1010112000000?0?001??11111110?0000021?0201101111100?110?10?0?1010001100?0111??????1??????????2??????????????10??110??????210010??1??????1?0?10?????????1??100??0??0?1?52????0???10?????1???0??2?01?1????0??0010011111200????????00?0??1100?10?1?????1?000?0???000??0?1?10?00????0?????????0?22???0????0010000?????0?100????001?1???????0?01000?1010??0110???20?11102300102?000?01??????2110010?0?10?????0????????????????0000??000????001??0???00?00?0?0?0?0??000???

Conchoraptor_gracilis ???0????0011100?0?00??1?????0?020?????????????01????1????110??010?0????????101?10??1000001???0??01??????0?000000??0?????????1??1?????????????0??????0?2???1?????00???110????????0??21?0?01101??1100?110?1??0??0?000??0???111??????1??????????2?????????????????????????????0????????????1???????????????????0??1??0????1?1??0???????1??????????????????????0????1???12?020000???00????0100??0?1????????00?0???0?????????????????0?????????0?????0?????00100100????0?100?11?001?1???????0?10000?1010??00?1???20???1????010??000?0???????11?001??0?10?????0???????????????????0??????????01??0???00?00?0???0?0??0?????

Gigantoraptor_erlianensis ???????????????????????????????????????????????????????????????????????????????????????????????????????????????????????????????????????????????????????????????????????????????????20?0001111011100?110?1??0?00?00?0?????????????????????????2??????????????????????????????????????????????????????????????????????????????????101012?0000?0??0??????????1???????????????????1?00??0100???110?110002?0??00?00000000?????00010?10?000??????????????????????????????????????????????????????????????????????????????????????????????111110????10001??????????????????????????????????????1?????????????????????0?0???

Incisivosaurus_gauthieri ??1000000101110100000100110?000111110001???0000100001100010000?1000?1001200001000?101000010000?00100100000000010010102011012101101?000001?0110???11?012001000??0010?11??1100???0?0?2011?01111111000?0?0000?0?00?0?0110??0?101200?00?00??1?2001101?10??10???????????????????????????????????????????????????????????????????????????????????????????????????????????????????????????????????????????????????????????????????????????????????????????????????????????????????0?????????????????0??????????????????????????????????????????????????????????????????????????????????????????????????????????1???????????

Microvenator_celer ???????????????????????????????????????????????????????????????????????????????????????????????????????????????????????????????????????????????????????????????????????????????????{1 2}0????11?1101100?????00?0?0????????????????????0???????2??2??????????????1101?110???????0010?01?1?11102??10100000?00100100010??0?10?0????????????1?11??00??2??????????????????????2002???????00?0??0?0??010110??02?00010000?000?0???????0????0?????????????????2???0010?00001000?1001101001???1010000?00?00100100?0101???20????????0?????????1??11?10121010000100??000???11?001??001000000120001101??????????????????????????0???

Oviraptor_philoceratops ???100??0?11?????1????10????0?0?0??10??????0??01????1????01000???00?????1?0101?????1?0?0??0??0?001?0????0??0??00???10???1?1?1??1?1?0?1?????????????????????0????00???????????????0?21???011?1111100?110?10?0??0?00???0????11??????1??????????2??????????????????????????????????????????????1??????????????0????????????????0???????????????????????10???????????1111??????????????0???1???1??0????????00?0???0????0???1????????0?????????0??????0????00?0000?????0?1???????0??1???????????????????????????????????????????????????????????????0?10?????0??????????????????????????????01???????????????????????????

Rinchenia_mongoliensis ???1?????011100?0?00??1?????????????0?????????01????1????0100?010?0?10?????101?0????0?00?????0??0??????00?000?00????????1???1??1???????????0???????????0???????????????????????????21?0?011?11111?0???0?10?0??0?0?0??0???111??????1??????????2??????????????????????????????????????????????????????????????????????????????0??????????????????????1?????????????1??1??????????????0???1?0??0?0???????????0???0????????1??????????????????????????????00?000??????102???111?01?1???????????????????????????????????????????????????????????????0????????0??????????????????????????????0???????????????????0????????

Buitreraptor_gonzalezorum ?????????0000??1?10???0?1???0??????????1???????0???????????00????0??????????????????1?10010??0?001?0????0???0?10?001???????????????????????????????????????0?????????????????????????1?0?0??0????01010????????????????????????????0??01?0020???00?00??00?0??110?010???201210110??12????010??????????0??1????0?????1?1??0?1??0???????1??01??10?2?01??????1??2???????102?1???????1100???101101001???????????0??1??????1?110???????????????????22????????????????0100??????????01?10?0????111?111?10????010????20112122??0112?0?1001???????1??1??????????????????????1????????11?20001????01?0011?10??1?01001?0???1????

Deinonychus_antirrhopus ??000000200001?111101000?00?000000010001000000010000110??1000001010?0??1???100?110??10???10??0?00??0???1001000?0??11031?110100?0???????????1?????????00??0????0?0??????????10??????0011?00??000??00?0?0001010?1001???110101011000002100?001000000?000100?0001111?10?00101200011?01011010020100000000000100101011101100??????0?????00120010010?1001?1????1?12?????1???21120000??110?0??1010?10?1?0???1000010001000?00?11100001101001001100111220000212?0010000001?00?1??1100001?1??0?0000?1110012010??0101???001111222001021010?0101011111111100001000001011111?00101??10?000002000110100110011?10?100011101001010???

Dromaeosaurus_albertensis ????????2?0??1??1?1??100????0???0?010??10100??0????011???100??0101??????1???????0?1210111???????0??0?????????000??01031???0?0??0001000001?00000100?0?0011?01010000?1110?10?100000000011000?00000?00?0?000100?111010111101010?000110?000?00000000000?0100?0???????????0???????????????????????????????????????????0?????????????????????????????????????????????????????????????????????????????????????????????????????????????????????????????????????????????????????????????1??????????????????????????????????????????????????????????????????????????????????????????????????????????????????????????????010???

Linheraptor_exquisitus ???000002000?1?1101?1?00?10?00000001000????000?0100???0001000101010????1110100?10?1?1000?100?0?00?200?00001010?0?0?10????????0000?????????????????1?10101????????????????????????0??0??000?000???00?0?0?010101????0??111???01?????02101?0?1000000??00100????1111010?1??00?0??11?11??1??????????????????????00???????10??????????10?0???000??001000?1101???1?1110??????????????1?????01?????10?11???01?0??1?????10??????????????????0???00?????????2????????????1?0?????????????????????????????20????010????01??????????????????1???????????????????00?101??111??10???10???0??2000110??011001??10?10?0?10???01010???

Mahakala_omnogovae ???????????????????????????????????????????????????????????????????????????????????100?00???????????????????????????????????????0???????????????????1001??1???????????10?0?10?????????????????????????????????????????????????????????????????????????????????????????????????????????????????????????????????????????40?1??0???????1??01??10?2?????????1????????????????????????????????????????????????1???10??????1110?0????????????????????????????????????1000?0???0???11?1???????0?11100???????????????????????????????????????1?11?111?0?0???0?0????????00??????0???00??0001????01?00??010?10??1??0?0??010???

Microraptor_gui 100????01000???1??????00??0?0??????????????00000???0?????1?0?0??????????1?0??????????0????0??0?00??????????????????????????????0?????????????????????????????????????????????????????10?0???000??00?0?0?????????01???1????10???01?02?0??0?10000???00??10??????????????????0?111??1??????00??0?1???????????100???0?1?004????1????10??1?001??1??1?01?1??????120100?1110?0?????????100???1111010?11???0????110??????000?110000000?10?10?0????0?22???02??1001000000100101?0?111??1?1???????0?011?1?200???010?1?0011121?2??011011010?1??????11?111????10???0?0??????00??????0?0?0???0001???10110011?10?11?0??01?101010???

Saurornitholestes_langstoni ???????????????????????????????????????1??????0??????????1??????????????????????????1?111?????????????????????????????1?????????????????????????????????????????0???????????????0????1??00??0?????????0????0????????????1????1???0???0??0?1000000?000100????1111010?1?1??200011?11?1011?12??000???00???1??10100110110?41?1010100????1??0???1??2?01?1????1??1?????????20??????????????????????0?????1???00?0?00?1?000??1?0?00?0?100???110011022??002???00100000?10?10210110100??1?0010011100100???????????????0???1????0?0??0??????????????????????????01011111???101??1?01100110001100101?0011?10?10?001?0?001?10???

Sinornithosaurus_millenii ?000??001?000??1111?1000??0?00001?11??01???0?00010????0000000?010?0?0??1??0100??101110100100?0?001201101?0?0??0??0010??????????0??????????????0??????????????????????????????????0?101?000?00000?00?0?0?010100??0?0??1????101010000?10010?1?00000?000100?0???????????????????????11?????1????????????????????????????04?????0???????1??0??????2?????????????01001111?20120000???10?0??1011?10?????????????0????????0????0000????1?????????1?22???02?2?00?0000?????0?1???11??01?0???????0?11??0?2011?????1???011121????011211010?1??????????1????????????????????????????????0??1001???001?00???10??1?0???1?001?10???

Tsaagan_mangas ??00000020000?011010?100110?000000010001???00000100?1?0001000101010?0??1100?????0?1110001100?0?001200?000010?000?00103????0?00000010010010010001011010101100???0000??10110?1000000?001?000?00000?00?0?0?0101011101011111?010101?0002101?0?1000000?000100???????????????00?0?????110??01?????????????????????????????????????????????????????????????????????????????????????????????????1???????????????????????????????????????????????????????????????????????????????????????????????????????????????????????????????????????????????????????????????????????????????????????????????????????????????????????????

Unenlagia_plus_Neuquenraptor ??????????????????????????????????????????????????????????????????????????????????????????????????????????????????????????????????????????????????????????????0??????????????????????1????????????????????????????????????????????????????????????0??????????????????????????????????????20?00101??????1??101??11011?0?0?1??0?0???????????????????????1???????????????????????110000?110?00100110211?????????????????????????????????????????????????????00???01000?210011100111??010000???111?20110101011?00011110??0011????11010?01?1112101000?10??00?????11??01??????????????????????1???????0????11??1??????????

Velociraptor_mongoliensis ??00000020000?0111101100110?000000110001???000001000110000?00101010?0??1120100?10?1110110100?0?000?0???00?10?000?0010?1?1?0100?00??0010??001?00??1?01001?000??0?00???11110?100000010011000?00000?00?0?000100??1?0?0111101010?110000?00110010000000000100?0??111?010????012000????10??0101???10?00????0?100101??1?01?0050?1010?0010001??010?1????01?110??1?1201000110020120000??100000?1110010?1?0??01?00010??1100??0?1?10000????00100?????0?2200002?2?0010000001000?110011100101?00?1000?01100020110?01011?0001111022001021010?0111??1?11?11?000010000010?1???100???0????0000?20001???101?0011110010001111?001010???

Epidexipteryx_hui ??????????????????????????????????????????????00???0???????0?001?00????????????????0???00?0??0?001??10????????00???0????????????????????????????????????????????????????????????10?2?0?00???1?10?00?10001??0?01?00???????010?011100?01??2120?101?0?1??00?0??????0????????????????12????000????100??????????00???0????04???0?0?0?2100???020?1??2??100????????1?0??????2?1????????00??0100?1?01?02?????0?0010?0?0??001????????0???????????0???22???0??????00001?010?0?200011??????????0???????0??0?0??????????0?010102?????110????1????????????????????0????????????0?????????1??0001???111000110????0?0???0??????????

Epidendrosaurus_ninchengensis ??????????????????????????????????????????????0????????????????????????????????????1???0????????01?????????????????????????????????????????????????????????????????????????????????????0011111?0?00?100?10?0?????????????????????????????1???1?1?0?1?????????????????????????????1???????0????1?????????????0?????0?1?????????????????????????1???????????1??????????2???????????????100?1?00??2?????????10?0??0?000????????0???1??01???0???22???0??00?000001???????????????????????????????????????????????????????????????????1????????????????????0??0?????????0???????????????????????001100??00???????0???0????

Scansoriopteryx_heilmanni 0?????????????????????????????????????????????????????0???10?001000????1???????1?????????0??????0????????????????????????????????????????????????????????????????????????????????0?20??00??01?11?00?100?10?0?0??1?0??0????1????????????????????????????????????????????????????????????0??0?0?100??????????00???0????030???00?0?10?0??001??10?0???0???1??11??????????2??2000??1??????0?0?1?01??2???1?0?0?00???0000001?11000011010?10100?0?0?220000210000100000?10?0?2001????01?1?00????????????0?0??????????{0 1}00??0?2?????1100???1??????2????1????1????????????????0????????????????1???0??0011?00?00???00??101?0????

Jinfengopteryx_elegans ????????1?01???1?10?0?????????001??????????????0???????????????????????????1??????????00????????0?0????????????2??0??????????????????????????????????100????????????????????????????0????0??0??0?0????????????1??????1?????0?000??0???????????1???????0?????????1????????????????????????????????????????????????????0??????????10?????02????????0?011?????2?????????2?1????????1?????1010????????????????????????????110?0?????????????????22?????????0?0?????????????????????1???????????????3????????????0?????????????????????????????????????????????????????0?????????????????????????????????????????????????

EK_troodontid ??????????????????????????????????????????????????????????????????????????????????????????????????????????????1????11??????????0????????0?????????????????10????1????1??0?????01?????????????????????????100????0????1???????????????????????0???????1???????0?????????????????????????????????????????????????????????????????????????????????????????????????????????????????????????????????????????????????????????1000010??0?1???????1?22?00?2???0000??????????????????????????????????????????????0?????????????????????????????????????????????????????????????????????????????????0011?00?1????0???1???10???

Byronosaurus_jaffeei ??000000110000010001?100110?000001110001????00001000??0000???0????110??11101110?0??0???????????????????????????????????????????000?11?0??1???0?00011?100111????010???11001010111?????0?000?00000?0110?0?010???????????????101?001002201?002000120000??10?0??10??????????2??0??????210???1??????????????1??1???????0????????????????????0???2????????????????????????????????????????????????????????????????????????????????????????????????????????????????????????????????????????????????????????????1???????????????????????????????1???????????0?0?01??11???????????0?????????????????????????????????????1????

Mei_long ??00000011011?00????0?00?12100000?11000?????00001000??01??11?0?11?0?0??12?01110?0?1100?00??0???001100?0???????10???11??????????0?????????????????????110??????0???????????????0??0??????00?0000??0110?0???????100????1????10?0?0?001201?0020???0???0???0?0??110000????101210110??1010??01?0?1?1000???00????00???00111040???1??0?1000?0001??2??0?0?0?1?0???12?????111120120000?1100000110100000010?111000110?0?????0???????????????10???00???2??000?1210010000001????????0??00??1?0011000?01100000????01001?0101??10???0??20??10?10?01??1110110?0?11?000?????????011?0?100??0112000110?00100011???1?1??10112101010???

Saurornithoides_mongoliensis ??1000??1100000??10?01?0????0???0?01???01000???0?????????1??????????????110??????????0???????0?00??0????????????????????1?01????00?11?????????1?????????????0?0??0?????????0??11?0?000?0?100000??011??0?0????????????1?????0?000??02101?0010001000001010?0???????????????????????1?????0?00??????0??000?00??0???000??0?0?10?000???001????00???????????????????????????????????????????????????????????????????????????????????????????????????????????0??????????????????????????????????????????????01?1???0??111023001020000?0???1111111011??????????????????????????????????????????01??????????1???0???1???1????

Sinornithoides_youngi ????????1?000???????????1???0?00??????????????00??????????????01????????????????0???????????????0??????10?????????0???????????????????????????????????????????0???????????????1????000??00??000??0110?0????????????????????0?000??0?101???1??0100?10?010?????????10????0??101???????????1????????????????????????????0??????0???10??1??01??1??0??1?0????1?12?????1???20120000???00?0??1??0?01???????2??0?10????????????10?0010??0?1?????????22???02????0?0000??????????????001?1???????0?1???0?00??????01???00?1210??00?0210?0??1???????1?011???????00?????????00??????0???0???????????0100011?0?10????011?101010???

Sinovenator_changii ??1???0001010??0010?1100111000?11???000??????000???011????????0?????????????????0????0?00???????01????????00??12??011??????????00010110001??010??01101011110110010??1110010101001??1?0??0???000??011100?0??0??1?0?0??????0101?00??0?2?1?0?10?010??0?01?0??????????10???0?210?10??01?????00???????0000?01??10000???0?1?40?1?00???10??1??01??2??0????????????1?????????20120000???10000?101?0????????????????????????0???10?001????????????????????????????0000???????????111????1???????0?11?00?2011??010????0011210??0011210010?10?01?11110110?0?11?00??11?111?0011???1000?10??0001???001??????00?10???1?1?1???1????

Troodon_formosus ??0???001?0?0?010?011100?10?0000?1??0??1??????00???0????????00????110??????100??0??00?0001??00??01??0?0???0?0?1???01030??1?11??0?00110000???001??010?1000001???010?1110?10?10?111??1?1??010?100??0110?00?????????0????????101?00?00?201?0?1000220?001010?????111?10?111??210110?01?10???1001001????????1??101??1??0?1050?1?10?0????011?0?0?20?0??1?????????2??????????????????1?00000110??0?0?1?0?10???01?0????????0????000010??0??0???00???220???????001?????????0????????????????????????????0010??0001???0??11??23??1?21000?0?0001?11120110?0?1???0?00110101??1????1?0100012000110?001?????100111?1?011?1??010???

Zanabazar_junior ??10000011000001010101001110000001110001????00001000??1???1????????????????111?????00?000100?0?00120100?0000?0???????????????????001100?0????01??0??110001010?0010???10????0??1110???0?001000000?0110?000????????????1100?1010001102101?0010001000001010?0????????????????????????????????????????????????????????????40?1010?00???0??00???2??0?????????1?12?????????????????????????????????????????????????????????????????????????????????????????????????????????????????????????????????????????????????????1??????0?????????????????????????????????????????????????????20001?????1?????????????1?????????????

Apsaravis_ukhaana ???????0????????????????????????????????????????????????????????????????????????????????????????0?????????????????1?????????????????????????????????????????????????????????????????0??001010011100?0?0??????????????????????????????????????2??????????????????110?1?001?12110??1?11???00??0?????????????????????0???50?1210?0?210?10?0200???2?0110????????1?1001???3000?000111000001101001100?00011001110001010000111110001????110?11?0?1???????????????????11000?01010??0???1?1?1?000?10????3012??1???1?00000?00000?1?22000??11?11112?2001?????1100???????????11????????11?????110?111?00??000110?11010?001000???

Archaeopteryx_lithographica 1100001010010001010?1?00?00?000100010001???0000010001100111100021?0?0??1100111110?1101000??0?0?000000?010?00?000???0?210??12?010?0?0??????????0??0?01110001???0???????1001011?00??10010000??0000?00?0?0?00?0??1?0?00?0??001010001002001?212010000?00??10?0??1???0?0???????10??????0?????????0??????????????00?????0??04?????0???20?01??01??1??2?00?0????1?111?00?1?102012?00001100?0001111?110020??1?000010????000?011?10?00?0??0??01???0?1?22?0?0??2??0000000???00?010???1001?1??0????0?01100?20?1????11???0011210000010?2101001??????11?0110?0??0?00??0??????0010?????00?0002000110?11110011?00?00?0?010?0??010???

Confuciusornis_sanctus 11?000?01?000000????0?01?0??00110011000????00000?0?01?11?111001?1?0?0???1?0????10???00?0?10??0?000?0????0?00?000??1?????????0??0?????????????????????1?????0?????????????????????0?10??000??0011000?100?0100??1?000??0???011??????1??????????2??????????????????0??????????????????????0????0???0??????0??100?????1??05?????0???20?01??1??0?????010110??????111001110?0????????1???00?11110110020??11001110001?1000011?11?00?0??01?0????0?1?22?0?0?????000000?1?000?010????0?1?1???????0???100?300?????1????0010?1?????1?211011?11?????2?20?1?????0??0??1??????00???????01??1??2110???111100??001?10?01??0?0??000???

Rahonavis_ostromi ??????????0???????????0????????????????????????????????????????????????????????????????????????????????????????????????????????????????????????????????????????????????????????????????????????????????????????????????????????????????????????????????????????????????????????????????012011110000000?1001000010010?041?001?000??10120010010020??????1?1112??????????????????1100000110??0????2?????????10001010000??????????????????????????????????00??????01000?21011110011100010000?0111002011??00111??0011210000011121011010001112?20110000100000101111110011100100100012000110100110011011110101110?001010???

Sapeornis_chaoyangensis ??0000000?010??1?1??0???????0??????1?00?????00001000??????11?002?00????12?0????10?11???0?100?0?0000?0?0????????????????????????????????????????????????????????????????????????????10??001001010?00?0?0?00?0??1?101??0??0010100?100?011?2120?2?????????0?0??????0??????????2??????211?????0??????0???????????????????05???01??0?210?0??1?0000??????0??????????????1112010?000?1100?0011011?11012?001?0011100?10000001111100010?10?10111?0?1022?0?020010010000001000?11010????1?1??010000?0?100?30110101101?01010?10001011010011010?11112?2?11??00100?0????????????1????????11??1001???11100010??0?10??10?0?0??000???

Jeholornis_prima ????????????0??????????????????????1???????????0???0????????????????????1?0???????????????????????????????????1????0??????????????????????????????????????????????????100????????0?????000??0?????????????????????????????????????1??????????1?0???????0?0???????????????????????????????2??????????????????0?????????3????1????20?02??02?0?1??????0??????????????11?2??????????0???0?1011?1{0 1}?0??????????10??001?0??1?111?0?????????????0?1?22?0???????000000????00?110?10??0??1?????????????????????????????????????????????1??1???????????????????????????????????????????????????????1???????????????????????????

Yixianornis_grabaui ???100??2?0?0???????????1??????????????????????0?????????????????????????????????????????????0??0?????????????????1?????????????????????0????????????????????????????????????????????0?0?0?10????00?????0?????1??0????????10100?101???1??12??100?0????10?011????110????????0????????????02?????00???????????0?????0??07???00????21?????1??000?2???11????????1100001003000?00011100?0?110110110???000100??1000100000010111?0?????????????0???22?????????01000??110?0?000?0??????1???????0?1?1???3012?????????1?01210000???20?01?010?????2?201???????0??0???????????11????????1?1???????11??0010?????0???????0???0????
